# Advancements in SERS: Revolutionizing Biomedical Analysis and Applications

**DOI:** 10.7150/ntno.106396

**Published:** 2025-07-09

**Authors:** Panangattukara Prabhakaran Praveen Kumar, Shivanjali Saxena, Rakesh Joshi

**Affiliations:** 1 Department of Biomedical Engineering, Institute for Quantitative Health Science and Engineering, Michigan State University, East Lansing, MI 48824, USA.; 2 NYU Grossman School of Medicine, New York, NY 10016, USA.; 3 DARVUN, Punjab 147001, India.

**Keywords:** Raman spectroscopy, microfabrication, top-down method, bottom-up method, bioanalytical applications, machine learning.

## Abstract

Surface-enhanced Raman scattering (SERS) has emerged as a powerful technique for bioanalysis, offering ultrasensitive molecular detection and identification capabilities. The signal intensity and reproducibility of Raman responses from analytes are primarily influenced by the surface roughness and nanogap architecture of plasmonic materials. Numerous designs, plasmonic nanostructures, and fabrication methods have been explored to optimize these factors. The precise nanogap ranging from 0.5 to 1.0 nm between the metallic nanoparticles and analytes offers significantly higher Raman enhancement, enabling single-molecule detection through SERS. With advancements in nano- and microfabrication techniques, the development of highly efficient SERS substrates has significantly enhanced the analytical performance in various biomedical applications. This review comprehensively examines the latest innovations in nano- and microfabricated SERS sensors, emphasizing their design, fabrication techniques, and functionalization strategies for biomolecular detection, bioimaging, and theranostic applications. Furthermore, we explore the growing role of artificial intelligence (AI) in optimizing SERS-based bioanalysis, from enhancing spectral data processing to developing machine learning models for pattern recognition and diagnostic applications. The integration of AI with SERS technologies holds great promise for revolutionizing point-of-care diagnostics, real-time biomarker monitoring, and personalized medicine.

## 1. Introduction

**Figure 1:**
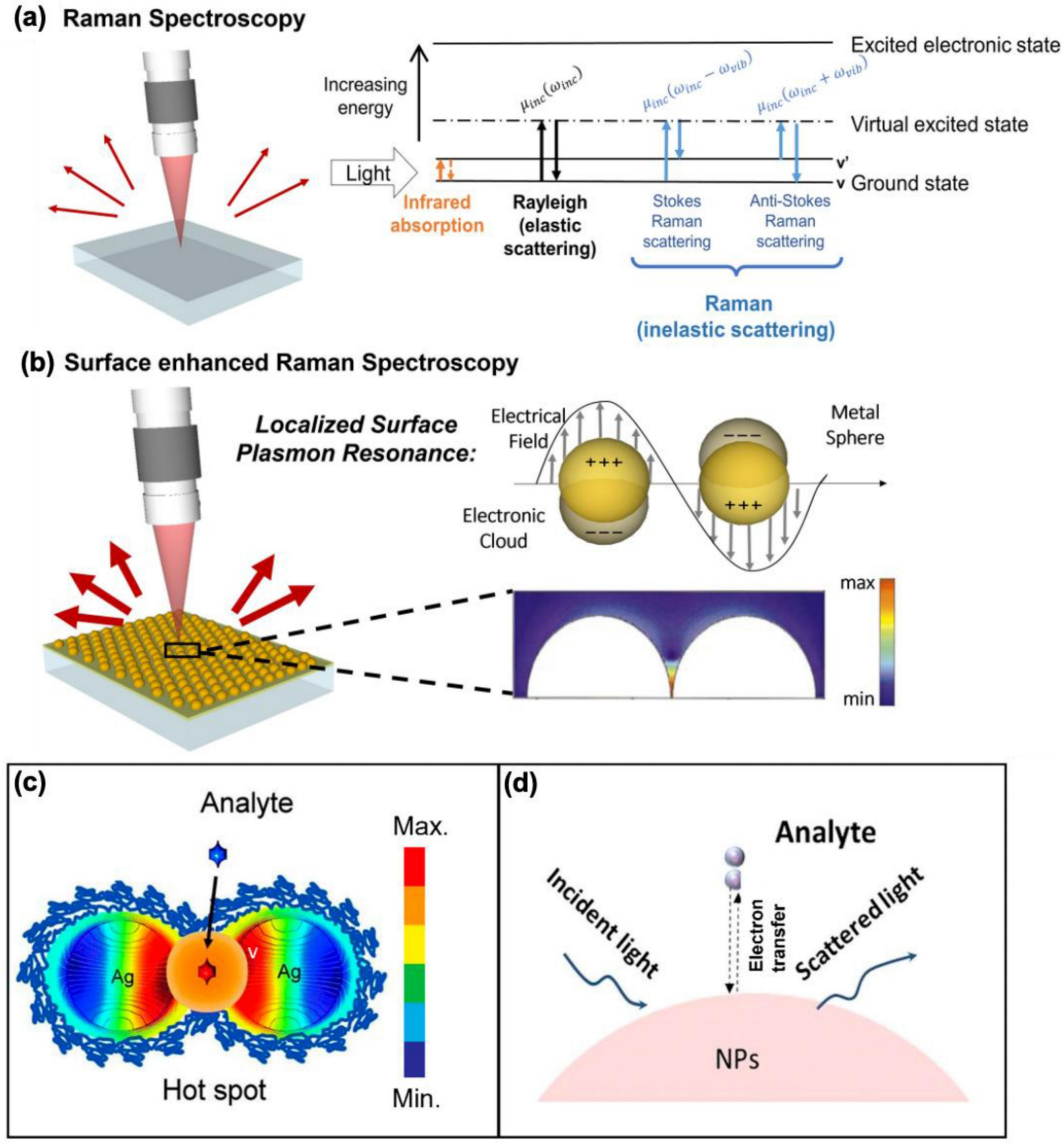
Comparison of Raman and SERS Phenomena. (a) Schematic representation of Raman Spectroscopy and its corresponding energy diagram, illustrating (from left to right) infrared absorption, elastic Rayleigh scattering, and inelastic Raman scattering, including anti-Stokes (left) and Stokes (right) processes. (b) An illustration of SERS and the LSPR effect, which arises from the collective oscillation of conduction electrons in a metal nanoparticle, resonating with the frequency of incident light. The color plot below depicts the electric field intensity profile in the gap between a dimer of two gold nanospheres, separated by 1 nm. The color scale is presented logarithmically. (c-d) The two mechanisms involved in signal amplification for Raman analysis. (c) electromagnetic field-based Raman signal enhancement using Ag nanoparticles. (d) chemical enhancement for Raman signal amplification due to the electron transfer between analyte and nanoparticles. Reproduced with permission from ref 8,10,11 Copyright 2022 Springer; 2015 RSC and 2017 Elsevier.

Surface-enhanced Raman scattering (SERS) has emerged as an effective spectroscopic technique, widely used in material science and bioscience research 1-5. Given the weak intensity of Raman scattering, conventional Raman-based methods are not ideal for detecting low concentrations of target molecules. However, in 1974, Fleischmann et al. discovered that using a rough metal electrode, the Raman signal for pyridine molecules is enhanced, a phenomenon now known as SERS 6. About two decades later, S. Nie and colleagues reported an enormous enhancement of the Raman signal when molecules were in the nanogaps between nanoparticle aggregates, achieving an enhancement factor (EF) of 10^¹⁴^, capable of detecting single molecules 7. Since then, extensive research has been conducted to elucidate the mechanisms behind these enhancements. Unlike conventional Raman spectroscopy, SERS provides significantly amplified signals **(Figure 1a-b)** 8. The signal amplification arises due to two key mechanisms: electromagnetic and chemical enhancement, with the former being the dominant factor 9. Electromagnetic enhancement arises from localized surface plasmon resonance (LSPR) near the nanostructured surfaces of noble metals like silver (Ag) and gold (Au). This generates "hot spots," which are regions of intensely amplified electromagnetic fields, typically found in the gaps, crevices, or sharp points of the plasmonic materials **(Figure 1c)** 10. On the other hand, chemical enhancement occurs when electron transfer happens between the analyte molecule and the nanostructure surface, facilitated by the matching of incident light energy with the electron transfer energy **(Figure 1d)** 11. This interaction alters the molecular polarization, enhancing the Raman signal by approximately 100-fold. Depending on the nanomaterials employed, the combined SERS enhancement factors can theoretically reach up to ~10^¹⁴^.

Despite numerous studies, developing SERS-active substrates that are uniform, reproducible, and cost-effective remains a challenge. Over the past few years, efforts have been made to fabricate SERS-active materials for various sensing and biomedical applications. Some studies have shown that clusters of metal nanoparticles with interparticle distances below 1 nm can produce intense “hot spots” and amplify SERS signals through inter-plasmonic coupling 12,13. However, single-molecule detection often faces issues with SERS signal fluctuations, limiting their broader applications. Recent developments indicate that micro-nano structures can significantly enhance SERS performance across various analytes 2,14. The degree of enhancement depends on factors such as the size, shape, and spacing of these structures. As a result, creating micro-nano structures has become a focal challenge for researchers, leading to the development of both top-down and bottom-up fabrication methods. Top-down approaches, such as lithography, offer precise control and reproducibility but face drawbacks like high costs, low throughput, and difficulty in scaling up 15,16. These methods typically achieve moderate EFs of 10^⁴^-10^⁷^ due to the challenges in creating sub-nanogap structures. In contrast, bottom-up approaches like colloidal nanoparticle synthesis are simpler and more cost-effective, with the potential for large-scale production 17. However, they face challenges with reproducibility and positioning analytes in hot spots 18. Nonetheless, when analytes are positioned within sub-nanogaps, the EF can reach values as high as 10^⁸^-10^¹²^. These colloidal substrates are suitable for biological applications such as cell, tissue, and *in vivo* imaging. To avoid some of the drawbacks for top-down and bottom-up approaches, hybrid approaches have also recently been used for fabricating SERS substrates 19. This review highlights key fabrication methods for micro-nano structures and their applications in SERS-based bioanalysis.

With well-defined nanostructures the EF for single molecule detection is greatly enhanced, but the complexity of the spectrum from samples makes the analysis difficult. The slight variations in the SERS spectrum are influenced by the molecular orientation on the substrate, and time-dependent fluctuations make it challenging to precisely extract specific information from the target analyte. To address this, traditional chemometric techniques have been employed for data processing and classifications. More recently, the growing use of artificial intelligence (AI) in data analysis has significantly enhanced the accuracy of these analytical outcomes. As AI continues to advance across fields like materials science, nanotechnology, and computer science, the potential for practical applications of SERS-based bioanalysis has expanded considerably.

## 2. Methods for fabrication of SERS substrates

In the fabrication of SERS substrates, top-down and bottom-up approaches are two primary methods employed.

### 2.1. Top-Down approaches

The most recent and ongoing method for the design of micro-nano fabrication is the top-down method, in which bulk materials break down into small nanostructures and commonly include lithography, imprinting, and micro-molding. The fabrication technique controls the homogeneity and roughness of the metal surface, which is responsible for the SERS activity and reproducibility.

#### 2.1.1. Lithography

**Figure 2:**
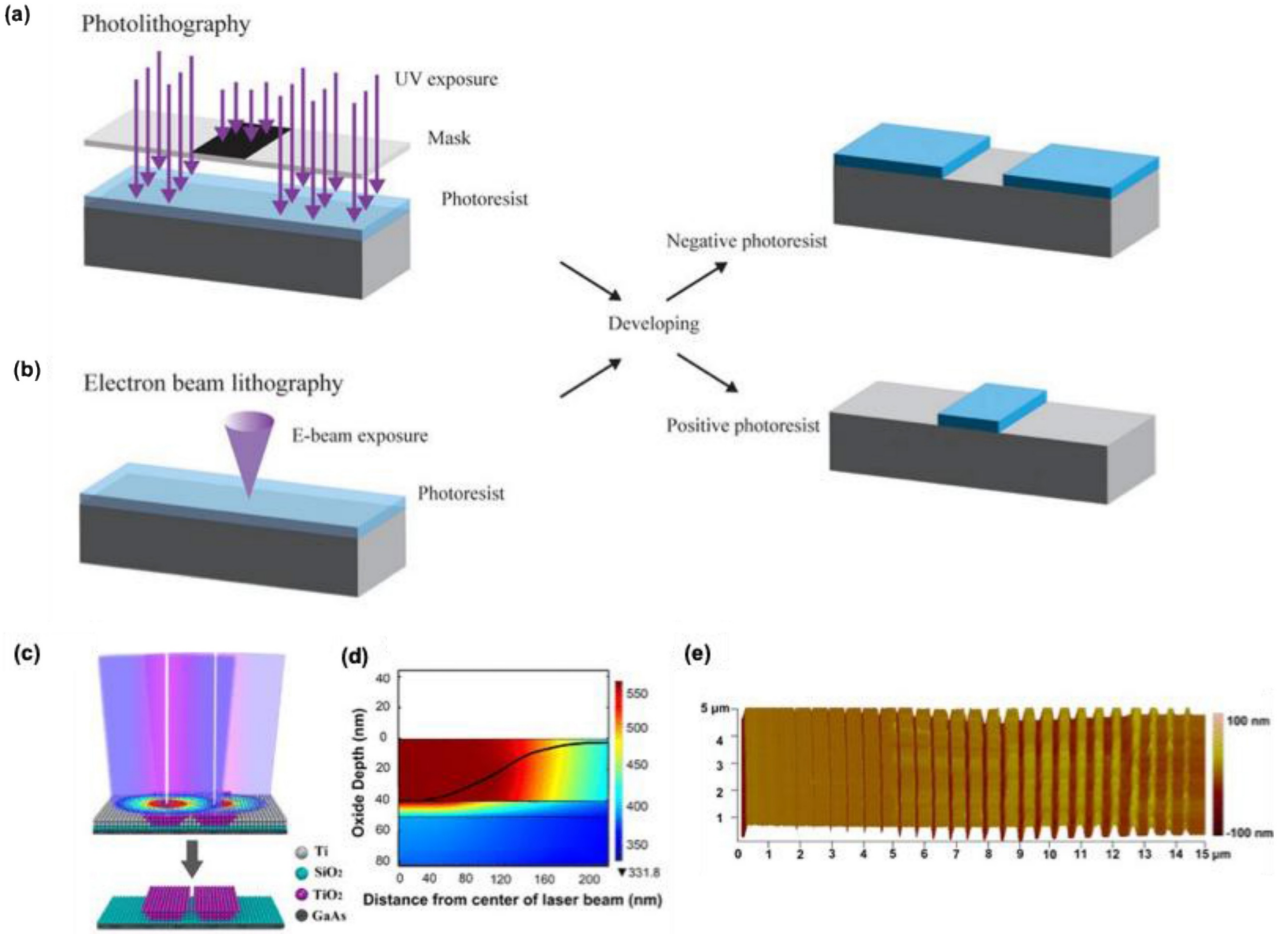
A pictorial representation for (a) Photolithography and (b) Electron beam lithography technique. (c) Schematic illustration of the nanogap fabrication using the two-laser-beam overlapping technique. (d) Simulated heat distribution from laser irradiation on the Ti/SiO_2_ bilayer structure. (e) AFM image showing the variation in slit widths as a function of laser power. Reproduced with permission from 21,22. Copyright 2024, Frontiers and 2020 American Chemical Society.

In the Lithography technique, various nano and microarchitectures can be developed with the aid of light (photolithography) **(Figure 2a)** or electrons (electron beam lithography, EBL) **(Figure 2b)** using a template called masked lithography technique or without a template known as maskless lithography 20,21. By using either of these techniques' various micro-nano structures for SERS applications can be prepared with the exact geometry and shape, needed for the enhanced Raman scattering.

##### 2.1.1.1. Photolithography

The visible light-based lithography techniques showed difficulties for the fabrication of nanostructures with nanogaps less than 10 nm. L. Qin et al. recently developed a cost-efficient sub-10-nm nanofabrication technique using laser direct-writing lithography with visible wavelengths 5 nm nanogap electrodes and arrays by Super-resolution Laser Lithography **(Figure 2c)** 22. They adjusted the size difference between the illumination source and the patterned feature by using a negative inorganic resist, which activates only at a specific thermal threshold. A low-intensity 405 nm laser beam heated only its central region, shrinking the activation area to about 60 nm, much smaller than the diffraction-limited spot size of over 200 nm **(Figure 2d).** Increasing the beam intensity expanded the activation area, allowing for easy control over the conversion zone. After scanning the beam, the sample was developed, removing the unexposed resist and creating a ridge with a width set by the beam intensity. Using two offset beams produced parallel ridges with a small gap between them, showcasing precise control **(Figure 2e)**.

##### 2.1.1.2. Electron beam Lithography (EBL)

**Figure 3:**
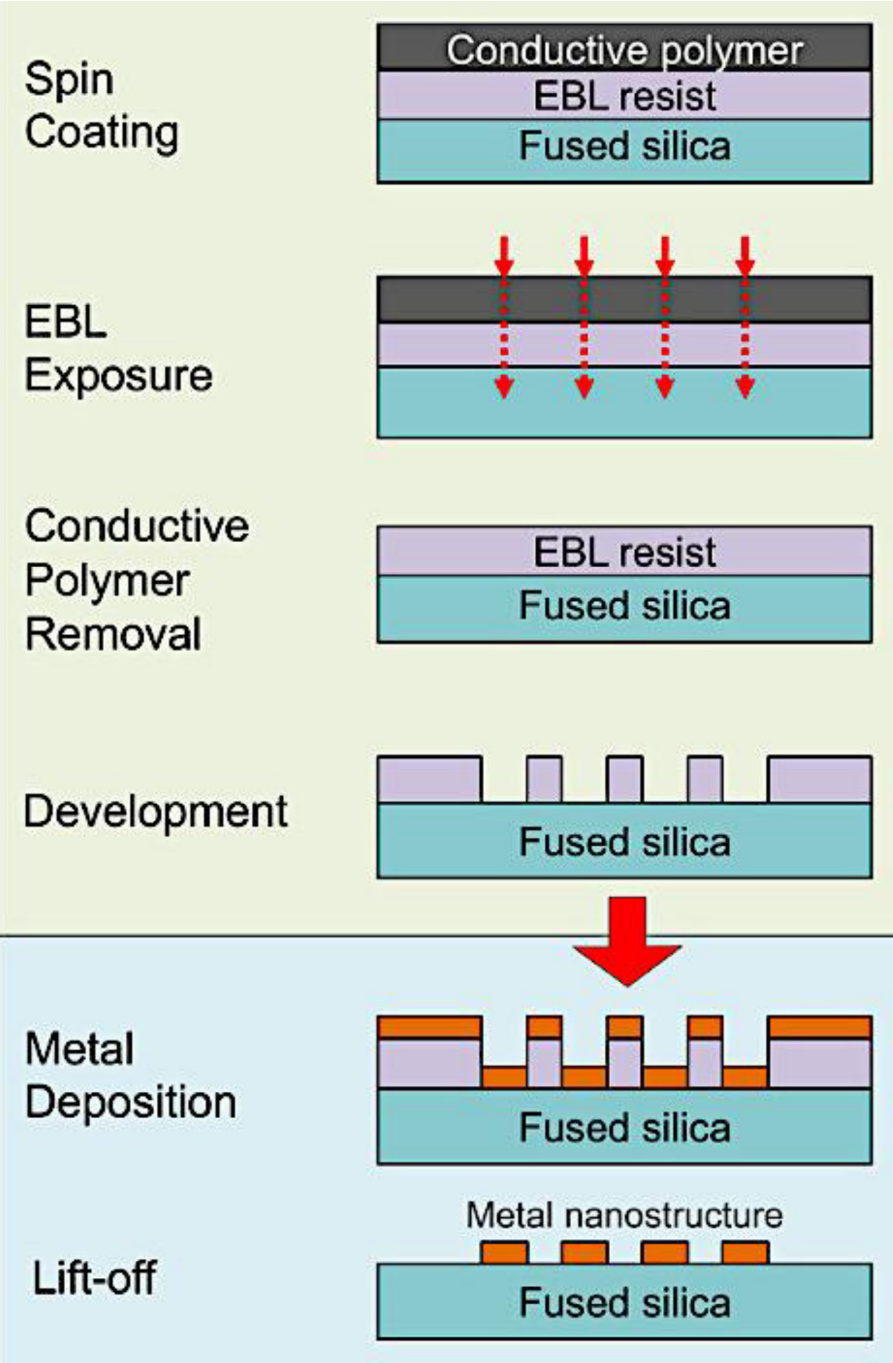
Illustration of the fabrication process for metallic nanostructures on dielectric substrates, showcasing the steps of electron beam lithography, metal deposition, and the subsequent liftoff procedure. Reproduced with permission from 23. Copyright 2015 MyJove Corp.

In EBL, a focused beam of electrons is used to write patterns on an electron-sensitive resist material (typically a polymer like PMMA) that is coated on the substrate (e.g., silicon or glass). Since the electron beam can be controlled with very high precision (down to a few nanometers), it allows the creation of extremely small features, including nanostructures with gaps in the range of 1-20 nm, which are ideal for SERS. After exposure to the electron beam, the resist undergoes a chemical change. In positive resist, the exposed areas are removed when developed, while in negative resist, the unexposed areas are removed. Once the pattern is developed, metal (typically gold (Au) or silver (Ag), which are plasmonically active) is deposited onto the patterned substrate via evaporation or sputtering. After metal deposition, the resist is removed, leaving behind the metal nanostructures with precisely defined gaps **(Figure 3)** 23. EBL plays a key role in creating ultra-thin metallic structures on flat substrates, a technique that has shown promise in enhancing SERS applications. These structures, typically arranged in periodic patterns, have been highlighted as effective SERS platforms.

Kahl et al. introduced EBL as a novel approach for engineering well-ordered SERS surfaces using Ag nanoparticles 24. Two primary fabrication methods were demonstrated: the lift-off process and the etching method. In the lift-off technique, a layer of Ag is deposited onto a patterned resist, which is then removed to leave behind organized nanoparticle arrays. Alternatively, the etching method uses reactive ion etching to create Ag gratings on a silicon wafer, followed by Ag evaporation. Beermann and colleagues successfully fabricated rectangular nanoparticles on smooth Au films, arranged either individually or in periodic arrays, and demonstrated a strong correlation between their reflection spectra and SERS signal enhancements 25. Yue et al. further advanced the field by precisely controlling the geometry of Au nanostructures, such as nanogratings, nanodiscs, and split rings, using both lift-off and plasma etching processes 26. Their findings revealed that nano gratings (60 nm line width) significantly outperformed other structures in Raman signal enhancement, with the lift-off process yielding better results than plasma etching. EBL has also been employed to fabricate more intricate designs, including coupled rings 27, bowties 28, and fractal bowties 29, the latter of which offers a tunable broadband spectral response. Cakmakyapan et al. demonstrated experimentally and with theoretical calculations, that each iteration of fractalization in a bowtie structure shifted the response to longer wavelengths, creating multiple hot spots that could be harnessed for improved SERS performance 29.

**Figure 4:**
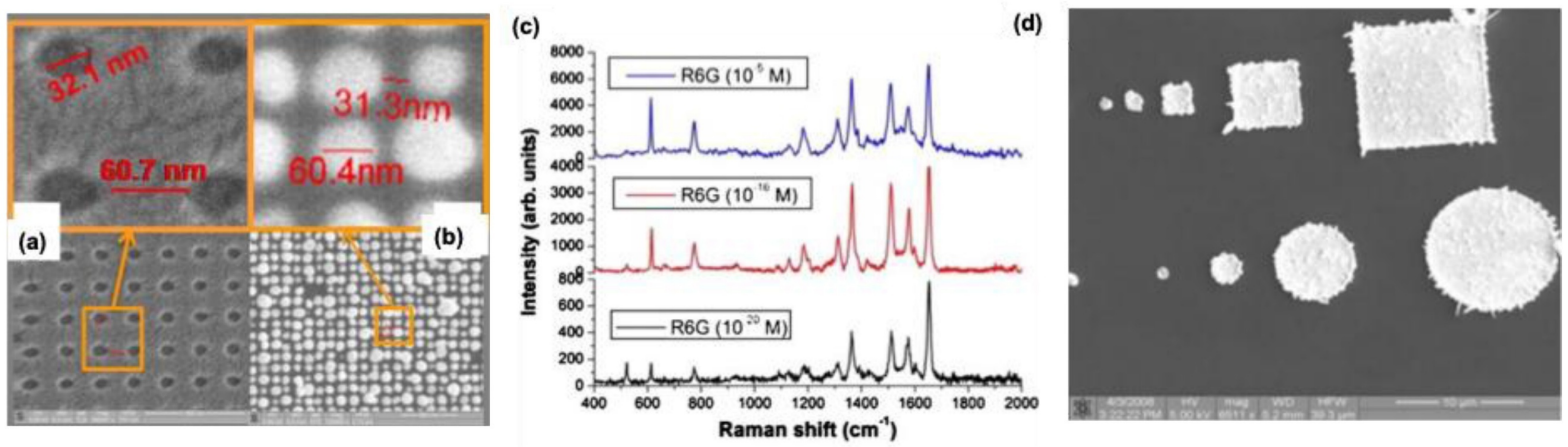
(a-b) SEM images of the lithography performed on Ag substrate (b) Ag deposition on the silicone surface. (c) SESR spectra obtained for different concentrations of R6G on the Ag surface. (d) SEM images for the AgNP deposition on Si wafer comprising micro and nanostructures. Reproduced with permission from ref 30. Copyright 2008 American Chemical Society.

Exploring multiple techniques for fabricating 3D structures offers significant potential, particularly through hybrid approaches that create substrates with well-ordered patterns and high-intensity hotspots for SERS. For instance, Hatab et al. recently used EBL with nano-transfer printing to develop highly sensitive Ag-based SERS materials 30. They fabricated various periodic arrays of Ag shapes such as square, triangular, and elliptical pillars—on a polydimethylsiloxane (PDMS) stamp using EBL. To remove the adhesive properties of the EBL resist (ma-N 2403), they applied a 50 nm thick modified cyclodextrin layer, allowing controlled deposition of Ag vapors onto the PDMS layer. This resulted in metallic nanostructures with precisely optimized nanogaps for enhanced SERS performance. Among the different patterns, Ag nanodisks exhibited the best SERS response, enabling the detection of crystal violet and mitoxantrone with detection limits of approximately 10⁻⁸ M and 10⁻⁹ M, respectively. Coluccio et al. employed EBL and a site-selective electroless deposition method to develop highly active substrates for SERS, specifically for enhancing the Raman signal of Rhodamine 6G 31. By adjusting the metal composition over time, they synthesized nanomaterials made of Ag, Au, or a combination of both, with varied nanoparticle properties **(Figure 4a-b)**. Rhodamine 6G served as the probe molecule in their SERS experiments, demonstrating that these new substrates exhibited remarkable sensitivity, detecting solutions with concentrations as low as 10^-20^ M **(Figure 4c)**. In this approach, the silicon surface utilized for deposition facilitates the reduction of silver ions to atoms without needing an external reducing agent, resulting in reproducible and highly active SERS materials **(Figure 4d)**. SERS in a 3D Au-triangular prism was studied by Petti et al.^32^. This substrate showed a significant enhancement in the SERS signal for detecting p-mercaptoaniline. Human prostate cancer cells have been tested, and it was observed that this substrate showed excellent sensitivity, particularly in the 1500-1700 cm^-1^ for the peptide functional group regions. These results are promising for the design of various biosensors for bio-applications. Even though the EBL technique possesses excellent reproducibility and fidelity, its high cost hinders the fabrication over a large area for practical application in SERS 33,34.

##### 2.1.1.3. Focused Ion Beam Lithography (FIB)

**Figure 5:**
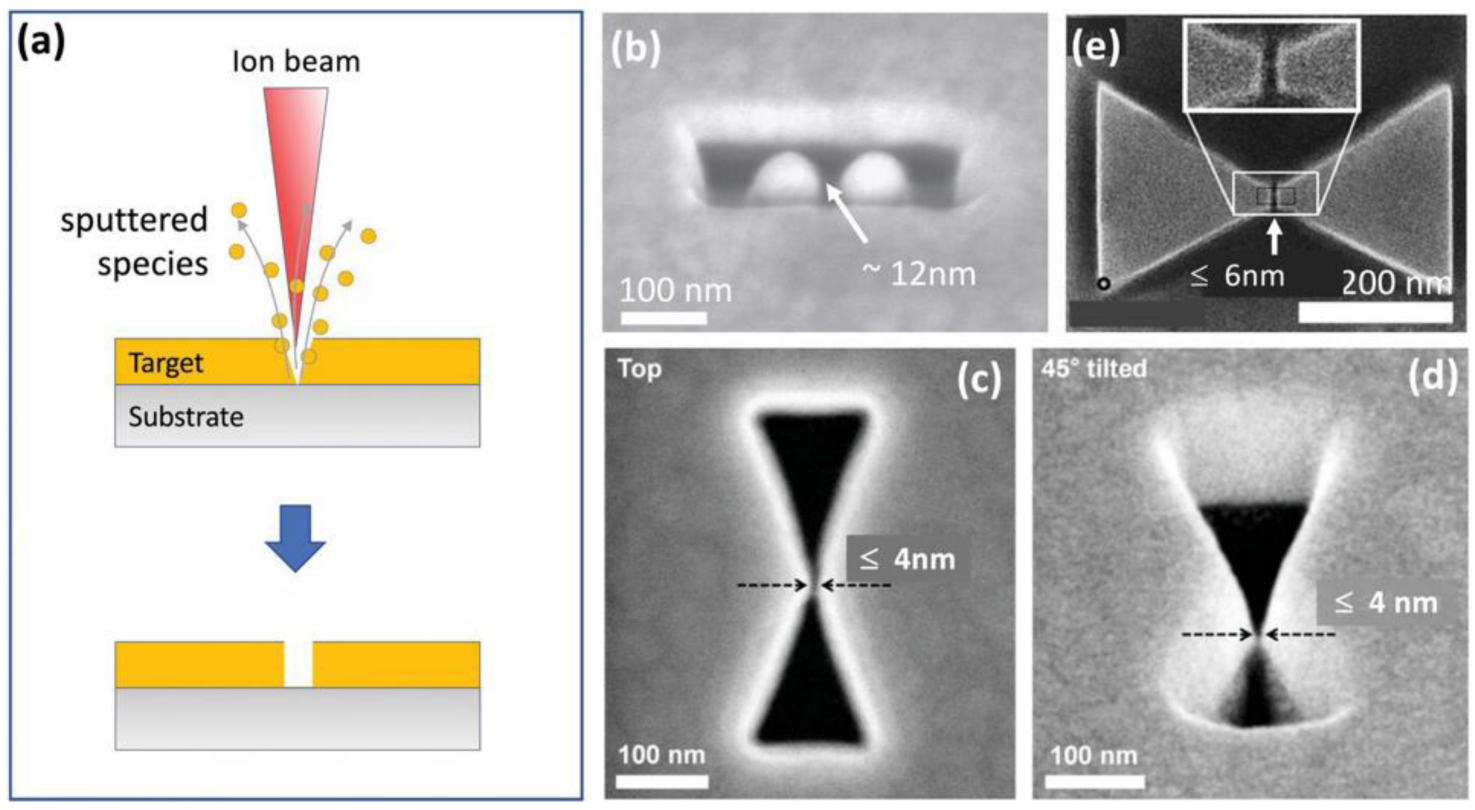
(a) Schematic representation of the focused ion beam (FIB) milling technique used to create nanogaps by sputtering atoms from the target material. (b) SEM image of a gold dimer antenna fabricated through Ga FIB milling, showcasing two gold islands separated by approximately 12 nm. (c,d) Top-view and tilted-view SEM images of bowtie-shaped air gaps in gold, featuring a minimum separation of approximately 4 nm, achieved via Ga FIB milling. (e) Example of a bowtie-shaped gold dimer with a gap width of approximately 6 nm, produced through a combination of Ga and He ion FIB milling for coarse- and fine-resolution patterning, respectively. Reproduced with permission from ref 36-38. Copyright 2013 Nature Publishing, 2014 and 2015 American Chemical Society.

In FIB lithography a finely focused beam of ions, typically gallium (Ga^+^), FIB can directly pattern or sculpt surfaces at the nanoscale with high resolution and accuracy (**Figure 5a**) 2,35. FIB lithography enables rapid, mask-free prototyping of SERS substrates with design flexibility and real-time optimization. It is compatible with various materials, including plasmonic metals like Au and Ag, and can be integrated with deposition or etching to create advanced hybrid structures for enhanced SERS performance.

In a typical setup, liquid gallium coats a positively charged tungsten needle, emitting a Ga^+^ ion beam. These ions strike a target, sputtering surface atoms. Ga FIB milling achieves a resolution of about 10 nm, limited by the 5 nm ion beam and ion-target interactions. Although faster than electron-beam milling, it alters the target's chemical composition by embedding ions. **Figure 5b** shows gold dimer antennae with a 12 nm gap, close to the standard Ga FIB milling limit 36. However, adjusting the scan path improves the resolution, achieving bowtie-shaped air gaps with 4 nm separations, as seen in **Figure 5c** and **5d** 37. To enhance patterning efficiency, a combination of Ga and He milling can be advantageous. Ga ions are employed for the initial coarse patterning, followed by He ions for precise fine structuring. Using this approach, Kollmann et al. successfully fabricated bowtie-shaped gold patterns with nanogap separations as small as 6 nm at the center **(Figure 5e)** 38.

K. Sivashanmugan et al. report the fabrication of Au/Ag nanorods, achieving an enhancement factor of 10^7^ 39. Their study focuses on how a multi-layer Au/Ag system influences SERS response. A silicon substrate undergoes sequential Au and Ag deposition to create a multilayer stack, which is structured using a focused Ga^+^ ion beam. A 30 nm spacing between nanostructures is maintained by adjusting beam current and etching time. The results demonstrate that multi-layered Au/Ag nanorods provide greater SERS enhancement compared to single-layer Au nanorods, achieving an enhancement factor around 10^6^ and successfully detecting low concentrations of influenza virus (10^6^ PFU/ml).

##### 2.1.1.4. Nanoimprint lithography (NIL)

**Figure 6:**
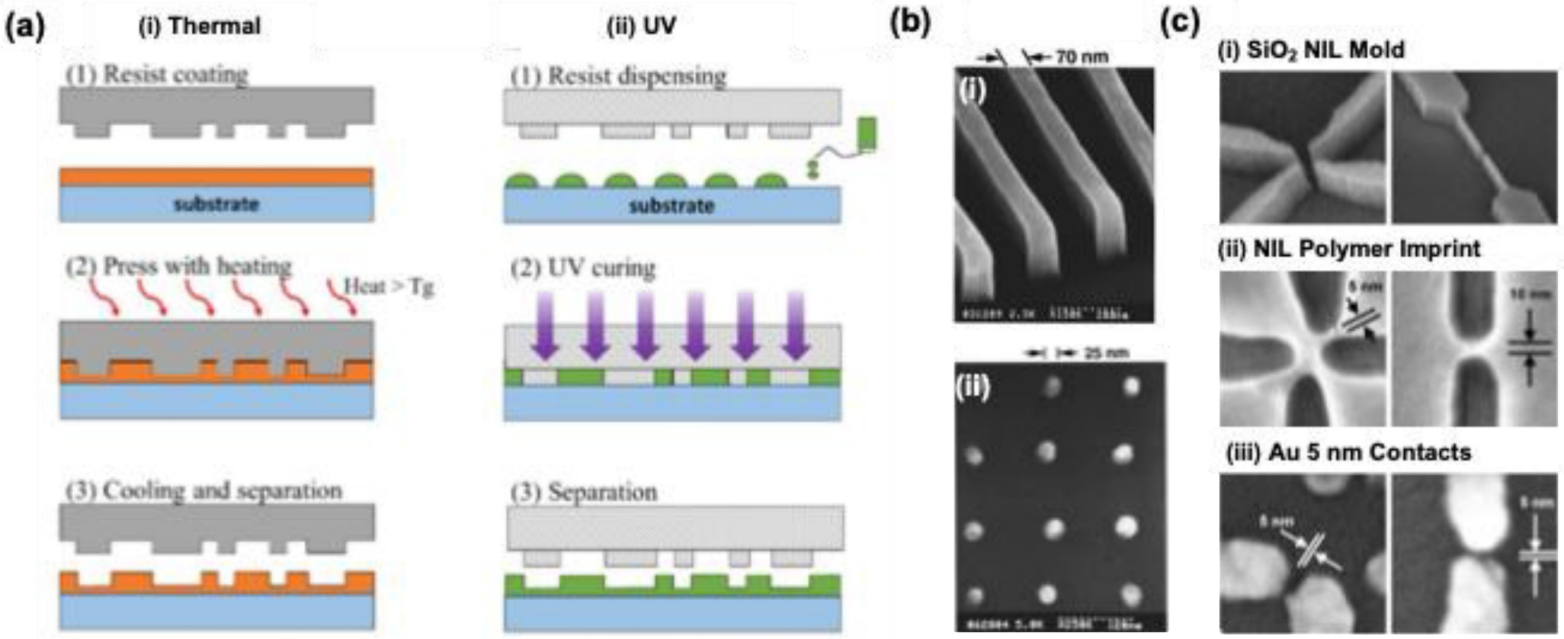
(a) A schematic for the conventional NIL method (a) thermal assisted (b) UV assisted NIL technique. (b) Scanning electron microscope (SEM) images showing: (i) strips with a width of 70 nm and a height of 200 nm, and (ii) metal dots with a diameter of 25 nm and a periodicity of 120 nm, both fabricated using thermal nanoimprint lithography (NIL). (c) SEM images display (i) a silicon oxide mold, (ii) the imprinted resin following UV NIL, and (iii) gold contacts after metal evaporation and resist lift-off, demonstrating 5 nm resolution in UV NIL for single-molecule contacts. Reproduced with permission from ref 40-42. Copyright 2020 MDPI publishers; 1996 and 2004 American Institute of Physics.

Nanostructure fabrication is significantly streamlined in nanoimprint lithography (NIL) compared to traditional optical nanopatterning techniques, as NIL employs straightforward mechanical processes.​ At the core of this method is a hard mold that is pressed into a polymer resin, allowing for the transfer of intricate nanopatterns **(Figure 6a)** 40. Two primary techniques in NIL include thermal and ultraviolet (UV) NIL. Thermal NIL relies on heat to cure a thermoplastic polymer resin, achieving impressive resolution levels down to 25 nm **(Figure 6b)** 41. Conversely, UV NIL utilizes ultraviolet light, resulting in rapid production speeds due to its lack of dependence on heating elements, as illustrated by its capability to create 5 nm nanopatterns with finely tuned gaps through the lift-off approach **(Figure 6c)** 42. Additionally, NIL can employ flexible molds made from materials like polydimethylsiloxane a technique known as soft NIL, which facilitates the creation of uniform nanopatterns across various surfaces.

**Figure 7:**
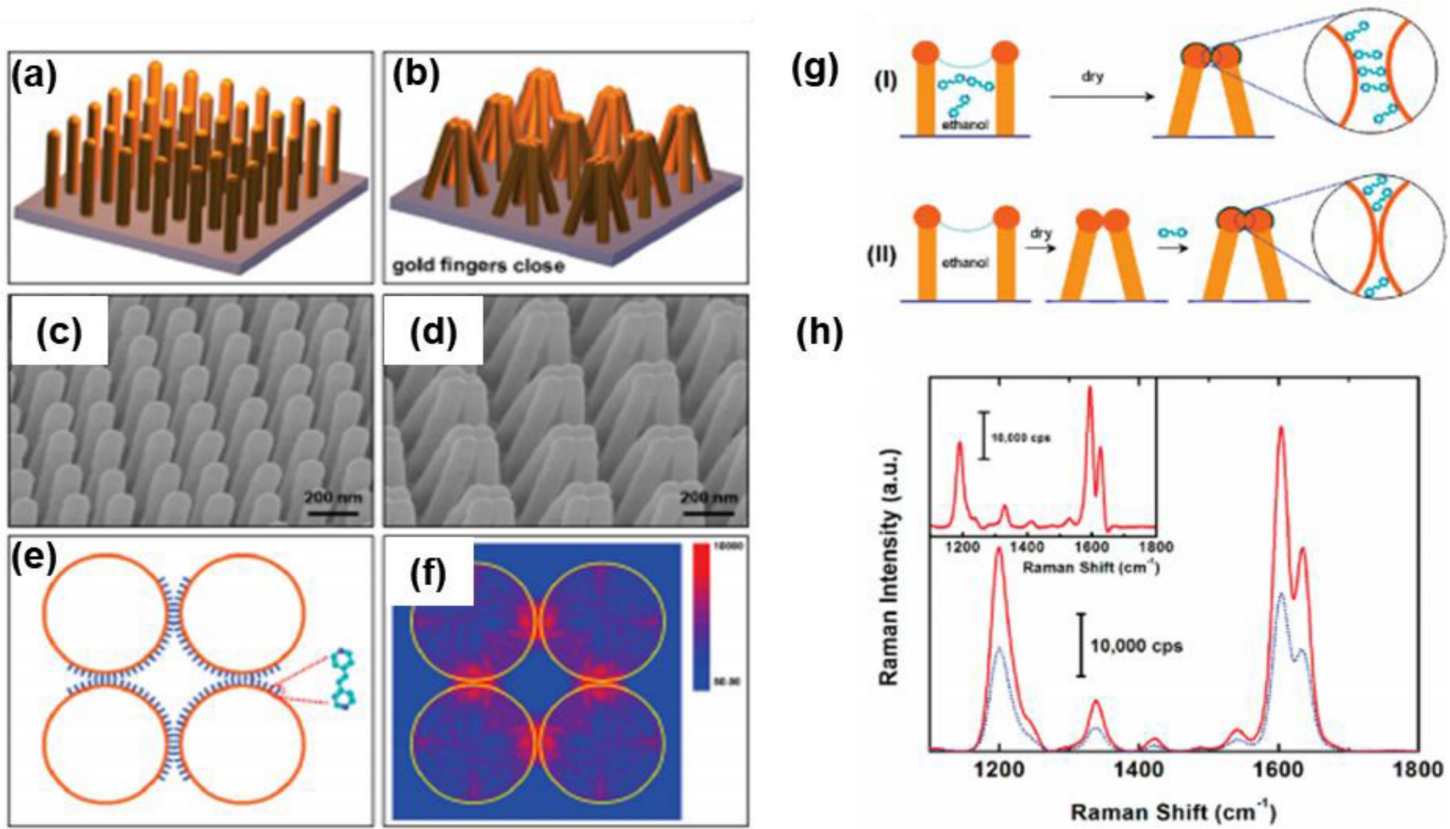
Schematic representation of the gold nanofinger (a) open and (b) closed-form, (c-d) SEM image for the open and closed Au nanofinger, (e) schematic of molecules trapped in the nanogaps, and (f) distribution of electric field intensity, (g) schematic illustrations: (I) fingers immersed in analyte solution and dried to close the fingers and (II) fingers immersed in pure ethanol to close the fingers before exposure to analyte solution, (h) comparison of Raman spectra of the analyte molecules from the case I (red spectrum) and case II (blue spectrum). Reproduced with permission from ref 43. Copyright 2010 American Chemical Society.

In 2010, Hu et al. presented a groundbreaking approach to creating a molecular trap structure in solution, leveraging the ability to capture analyte molecules 43. This innovative structure is composed of nanoscale polymer fingers, crafted from gold with a diameter of 100 nm, using NIL techniques **(Figure 7a-e)**. These nano fingers are meticulously engineered to ensnare analyte molecules, effectively closing themselves to form hot spots for SERS. The closing mechanism results from capillary forces during the drying phase of the liquid **(Figure 7e-g)**. Moreover, the precise control over the self-limiting gap size between the fingertips allows for significant SERS enhancement, facilitating the detection of sensitive molecules. Notably, the enhancements observed for trans-1,2-bis(4-pyridyl) ethylene and 4-mercaptophenol were ten times greater in the closed finger configuration, while Rhodamine 6G exhibited an impressive thirty-fold increase in intensity **(Figure 7h)**. ​This work underscores the significance of employing a top-down fabrication strategy for diverse SERS substrates, which offers advantages unattainable through traditional 2D or 3D material assembly.​ Furthermore, these techniques hold the potential for broader applications, including in the fields of plasmonic, metamaterials, and other advanced nanophotonic systems.

Ferchichi et al. developed a flexible, disposable SERS substrate using NIL technique 44. This involved two nanoimprint steps, creating a double-layer flexible plastic foil with gold nanostructures imprinted from a silicone mold. The SERS characterization of both the master devices and the polymer replicas showed significant Raman signal enhancement for Rhodamine 6G and Ibuprofen, allowing the use of a reduced gold layer thickness of 50 nm, making it cost-effective for SERS applications. In a separate study, Suresh et al. created a highly transparent and flexible SERS substrate using polycarbonate sheets and the NIL technique to deposit Au nanocones 45. The study examined two gold coating thicknesses (35 and 70 nm) for detection of crystal violet. Studies showed a limit of detection of 1 μM for crystal violet with an enhancement factor of 1 x 10^5^. This nanosheet provides a reproducible, economical, transparent, and flexible option for future nanoplasmonic sensing applications.

##### 2.1.1.5. Micromolding

Molding is a process that entails transferring a topographic pattern from one material to another through the use of a liquid precursor, which solidifies during the molding process; this technique is referred to as replica molding (REM) 46. Additionally, various methods exist for the fabrication of two-dimensional (2D) and three-dimensional (3D) materials, such as micro-transfer molding (μTM) 47. In μTM, a polymer is deposited onto the surface of a PDMS mold, while excess material is eliminated through reactive ion etching (RIE). Another notable technique is micro-molding capillaries (MIMIC), where the PDMS mold is positioned on the substrate to create a network of microchannels. A liquid drop is then introduced at one end of the channel, allowing it to spread throughout the microchannels, ultimately generating specific patterns 48. This technique has been utilized to fabricate various optical components, including fibers, waveguides, and polarizers 49.

Furthermore, microfluidic and nanofluidic methods are integral to the MIMIC technique, being primarily employed in the synthesis of biochips for the creation of nano-microstructures. Wu et al. reported the development of SERS-modified microfluidic system featuring Ag-coated Au nanorod-decorated PDMS microchannels for the detection of thiocyanate (SCN) in human serum and saliva 50. The findings indicated that thiocyanate detection in human serum is effective across a concentration range of 4-256 μM. Significantly, the analysis of human saliva was shown to differentiate smokers from non-smokers due to elevated thiocyanate levels in smokers. Additionally, research conducted by Pallaoro and colleagues demonstrated that it is possible to detect low concentrations of cancer cells to distinguish between cancerous and non-cancerous cells 51. In their study, a combination of cancerous and non-cancerous prostate cells was incubated with surface-enhanced Raman spectroscopy biotags (SBTs), which were synthesized from citrate-protected Ag monomers (45 nm) and aggregated into small clusters using phosphate and hexamethylenediamine. These clusters were then coated with polyvinylpyrrolidone to ensure stability and minimize nonspecific cellular interactions, employing modified bovine serum albumin. Subsequently, the small Ag clusters were injected into a flow-focused microfluidic channel. The specificity of this detection method was validated using Neuropilin-1 (NRP-1) overexpressing prostate cancer cells compared to normal RWPE-1 cells. ​Results indicated that this method could successfully detect cancer cells even at a low concentration of 2%, amidst a mixture of normal cells, utilizing SERS.

**Advantage of top-down methods**

- Precisely controlled nanostructures
- Well defined and evenly distributed hot spots
- Stable SERS hot spots
- Highly uniformity and reproducibility
- 3D nanostructures with enhanced performance

**Disadvantage of top-down methods**

- Complexity
- Time consuming
- Need expensive equipment
- Laborious and complicated process
- Chemical pollutions are a threat in the substrate

### 2.2. Bottom-Up approaches

Bottom-up approaches for the fabrication of SERS materials involve assembling structures from the atomic or molecular level to create nanoscale features that enhance the Raman signal. These methods, such as template methods and layer-by-layer assembly, allow for the creation of uniform and functional SERS substrates that include specific properties beneficial for detecting analytes at low concentrations.

#### 2.2.1 Synthesis of Plasmonic Metal Nanoparticles or Colloids

The synthesis of colloidal noble metal nanoparticles, such as gold (Au) and silver (Ag), via the reduction of precursors using reducing agents in the presence of stabilizers, represents a cost-effective and efficient bottom-up approach 52-54.​ This technique has garnered significant attention for its application in SERS substrates due to its shorter preparation time compared to alternative methods. The ability to control the size, shape, and composition of plasmonic nanoparticles in colloidal solutions is crucial, as these parameters directly influence the localized surface plasmon resonance (LSPR) properties. Modifying the LSPR characteristics enhances the electromagnetic field around the nanostructures, leading to an improved enhancement factor in SERS applications. Nevertheless, challenges remain, including the precise positioning of analytes within the "hot spots" of these colloidal nanoparticles, which can adversely affect signal reproducibility and intensity in Raman spectroscopy.

Benz et al. investigated the use of citrate-stabilized AuNPs with various sizes (40, 47, 56, 68, 70, 78, 86, and 98 nm), employing ascorbic acid and trisodium citrate as a reductant alongside chloroauric acid as a precursor for SERS substrates 55. ​They observed size-dependent enhancements in the SERS intensities of biphenyl-4-thiol, recording increased Raman intensities at 1583 cm⁻¹ with larger AuNPs. In a separate study, Mulvihill et al. fabricated polyvinylpyrrolidone capped AgNPs in 1,5-pentanediol to examine how particle shape influenced Raman intensities of benzenethiol, relative to the degree of octahedral etching 56. The SERS intensities for various particle shapes octahedrons, mildly etched particles, and octopods displayed comparable results under 514 nm excitation. However, the mildly etched particles and octopods produced notably higher SERS signals at 633 nm and 785 nm, respectively, achieving enhancement factors of 3×10^4^ for a single octahedron and 5×10^5^ for both the mildly etched octahedron and the octopod at their respective optimal excitation wavelengths. Additionally, Tian et al. synthesized approximately 150 nm AuNPs using the seed-mediated growth method to create gold nanospheres, nanotriangles, and nanostars 57. They found that the SERS intensity of rhodamine 6G (R6G) at 785 nm was maximized for gold nanostars, was intermediate for gold nanotriangles, and was minimal for gold nanospheres. These findings align with the notion that enhanced electromagnetic fields at hotspots are necessary for SERS enhancement.

The efficiency of SERS varies significantly depending on the morphology and aggregation state of nanoparticles. Numerous reliable techniques are available for producing spherical metallic nanoparticles, which are frequently adopted as SERS substrates due to their durability and straightforward manufacturing processes. ​Interestingly, increased SERS efficiency is often observed when these spherical nanoparticles experience partial aggregation 58,59.​ This phenomenon occurs because the junctions formed at the intersections of the nanoparticles create "hot spots," where substantial field enhancements happen, enabling detection capabilities down to individual molecules. Despite this advantage, achieving precise control over nanoparticle aggregation remains a challenge, which in turn complicates the reproducibility of SERS signals. For clarity, we define "hot spots" as specific regions where intense local field enhancement occurs, resulting from surface plasmon resonances, typically found in the gaps within metal architectures. Research has highlighted that these hot spots can dramatically amplify SERS signals, achieving enhancements of up to 15 orders of magnitude. Additionally, the study of anisotropic metal nanoparticles has emerged as a promising alternative for generating these hot spots.

**Figure 8:**
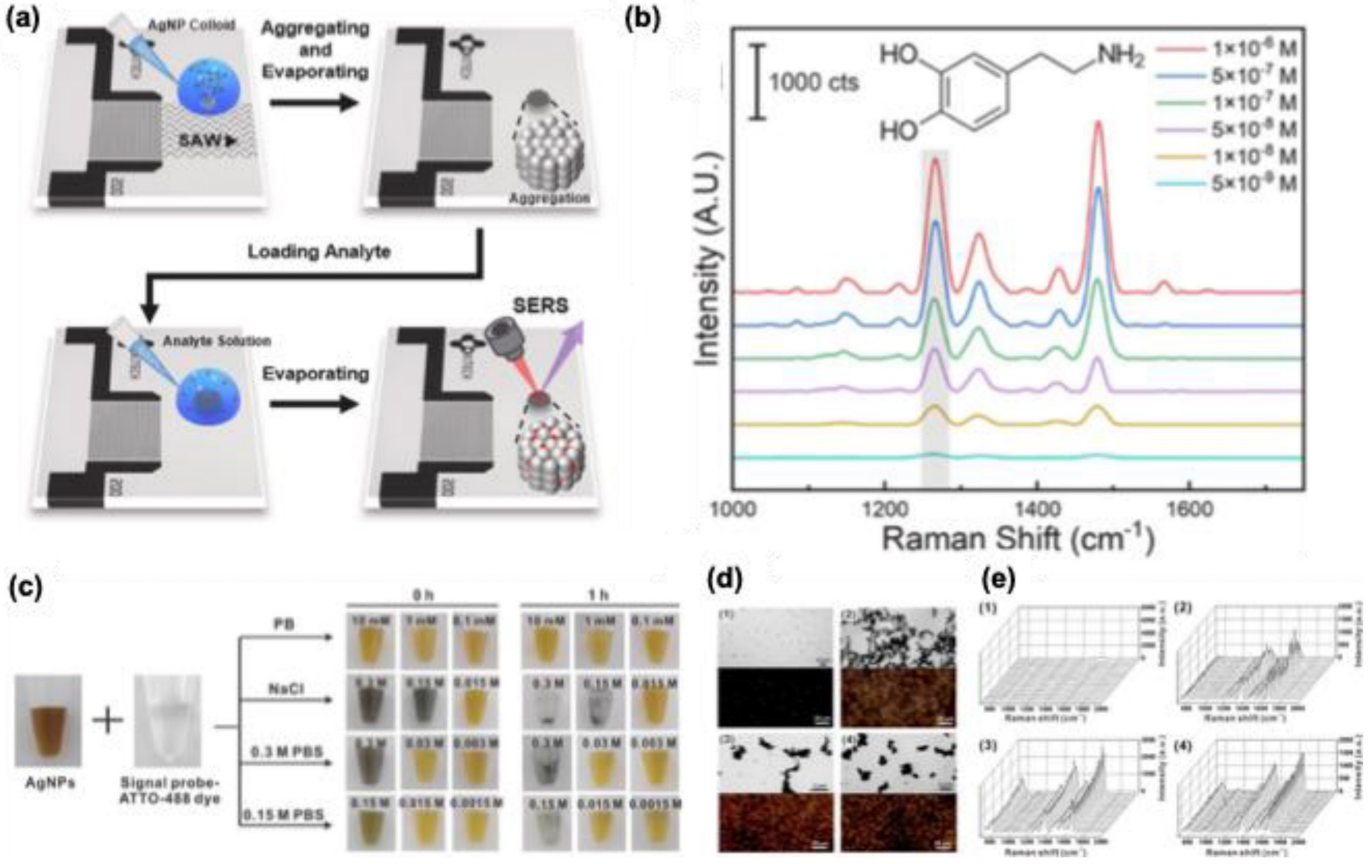
(a) A schematic representation of the SAW technique for SERS-based dopamine detection. (b) SERS spectra for dopamine with varying concentrations from 1 × 10^-6^ to 5 × 10^-9^ M. (c) A photograph showing the salt-induced color change from AgNPs using PBS and NaCl with different concentrations and time. (d) Transmission electron microscope and dark field microscopic images for the Ag nanoclusters formed using various aggregating agents. (e) SERS spectra for the signal probe ATTO-488 dye under different salt-induced aggregation conditions using AgNPs. The results showed that PBS had 0.15 M concentration and showed enhanced Raman signals for the dye at 1348 cm^-1^. Where (1) PB (10 mM, 0 h), (2) NaCl (0.3 M, 0 h), (3) 0.3 M PBS (0.3 M, 0 h), and (4) 0.15 M PBS (0.15 M, 0 h). Reproduced with permission from ref 58,59. Copyright 2024 Elsevier and 2022 American Chemical Society.

Par et al. introduced a new technique called surface acoustic wave (SAW) for the clustering of AgNPs with precise nanogaps for the detection of dopamine. As shown in **Figure 8a**, a solution of 50 nm size AgNPs was aggregated at the center of a droplet with the aid of SAW. After evaporation of the solution, nanopatterns of AgNPs were developed with precise nanogap ∼1.5 nm. The developed nanopattern is used for the detection of dopamine with a sensitivity of 4.28 × 10^-9^ M **(Figure 8b)**. In one of the studies by Jang et al., the use of AgNPs with different shapes (sphere, triangular plate, nanostar, and cube), and controlled aggregation of these NPs using PBS showed enhanced signal intensity for DNA targets such as RdRp, E, and N genes in SARS-CoV-2 **(Figure 8c)**. Studies showed that using 0.15 M PBS as an aggregating agent, an enhanced Raman signal was observed for the signal probe ATTO-488 dye **(Figure 8d-e)**. Moreover, studies showed that nanoplates and nanostar-based structures showed a sensitivity of 10 attomolar levels for the RdRp gene target.

### 2.2.2 Self-assembly-based approach

Self-assembly is a process whereby disordered particles or materials spontaneously organize into structured arrangements due to specific interactions. This technique is among the most employed bottom-up methodologies for Raman substrates. It is facilitated by mechanisms such as capillary force, dipole-dipole attraction, electrostatic interaction, and chemical bonding 60,61. ​The self-assembly approach offers several advantages, including the ability to create complex structures at a low cost and with ease of manipulation.

#### 2.2.2.1. Self-assembly in solution

Among the various strategies, chemical immobilization using various linkers on solid supports is widely used for the fabrication of self-assembled NPs for SERS. The surface of solid support can be functionalized by amine, thiol, or silane groups and immerse the solid substrate in nanoparticle solutions. One of the earliest works done by Nathan et al. used gold nanostars, which were then deposited on a non-conductive polydimethylsiloxane surface for various sensing applications such as pesticides from apple skin using SERS 62.

**Figure 9:**
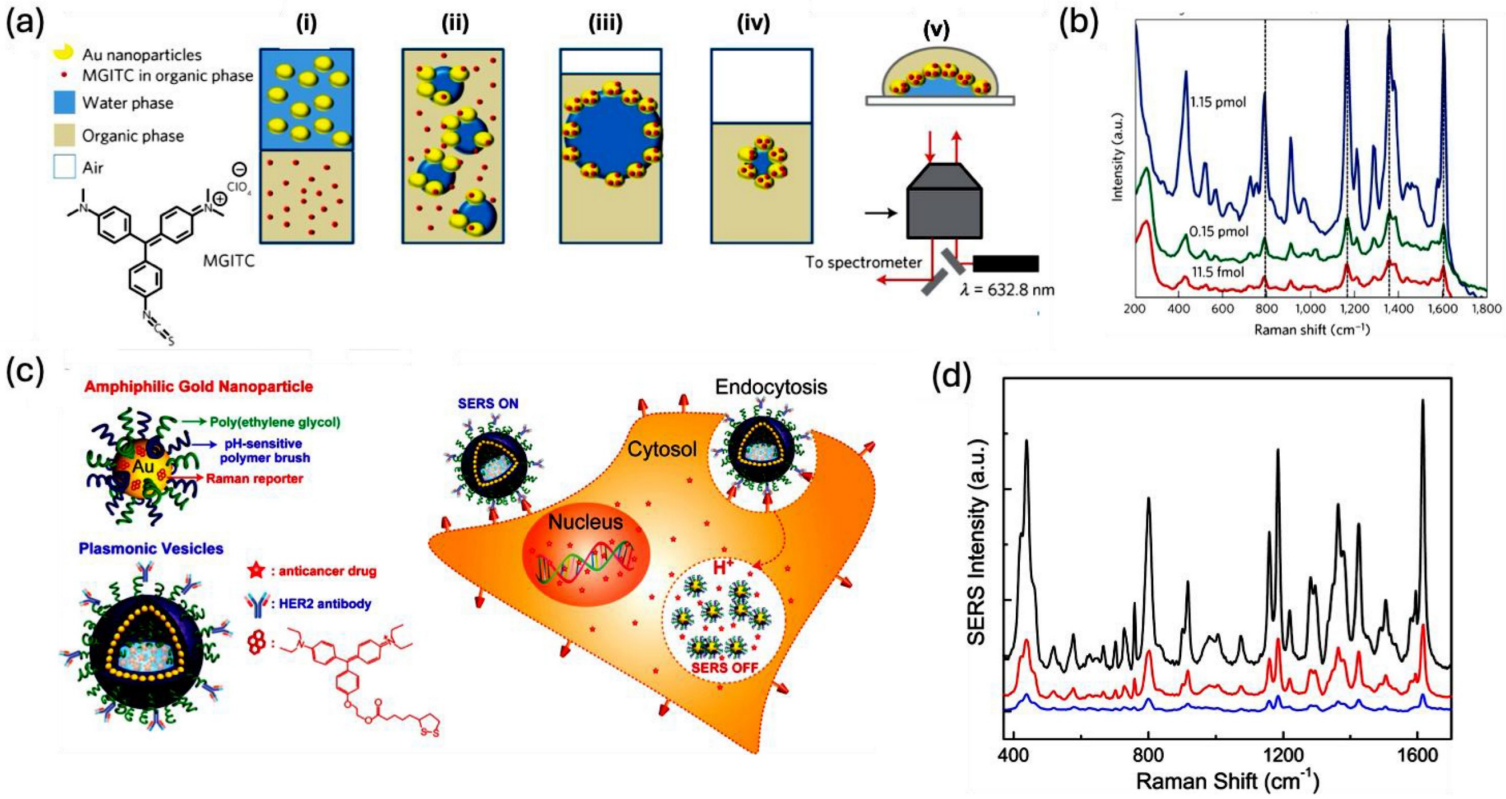
(a) Schematic representation for the self-assembly pattern of AuNPs at the liquid-liquid interface. (i) The analyte is introduced into the organic phase, while nanoparticles are dispersed in the aqueous phase before the LLI formation. (ii) Using gentle agitation an emulsion is formed at the interface. (iii) The smaller droplets of the emulsion reorganize to establish a layered liquid interface composed of nanoparticles and malachite green isothiocyanate. ​(iv) Water is extracted from the droplet, resulting in the nanoparticles being brought closer together. (v) The droplet is subsequently placed onto a coverslip. (b) Concentration-dependent SERS spectra for malachite green isothiocyanate on the nanoparticle. (c) A schematic representation of the plasmonic nanovesicles and their cellular binding, and uptake. Under acidic pH, the vesicles show a decreased SERS signal intensity due to the pH-responsive nature of nanovesicles. (d) Time-dependent SERS spectra of SKBR-3 cells using targeted nanovesicles. The black line for 30 min of incubation, the red line for 60 min of incubation, and the blue line for 90 min of incubation of nanovesicles with SKBR-3 cells. Reproduced with permission from ref 64,65. Copyright 2013 Nature Publishing Group, and 2019 American Chemical Society.

Cecchini and colleagues explored self-assembly methods to fabricate SERS sensors at a liquid/liquid interface (LLI), which led to the creation of a high density of hotspots throughout the structure **(Figure 9a)**. They mixed AuNP and the fluorophore malachite green isothiocyanate dissolved in water with the organic solvent 1,2-dichloroethane to produce an emulsion. This process resulted in the organization of nanoparticles at the LLI. ​Subsequent Raman spectroscopy using a 632.8 nm laser revealed a limit of detection for malachite green isothiocyanate at around 1 fmol, highlighting the method's practical effectiveness **(Figure 9b)**.​ Furthermore, the technique's adaptability was confirmed by applying it to non-resonant dyes like mercapto-5-nitrobenzimidazole and 4-methoxy-α-toluenethiol, along with various other analytes, achieving detection limits in the picomole to nanomole range.

Lin et al. developed a self-assembly technique for LLI to synthesize various types of faceted gold nanocrystals, including cubes, concave cubes, octahedra, and rhombic dodecahedra. Notably, the rhombic dodecahedra exhibited a remarkable SERS response, achieving a high enhancement factor of 1.2×10^6^ for crystal violet 63. The study found that the SERS response of crystal violet correlated positively with the size of the nanocrystals, provided they maintained the same morphology. Finite-difference time-domain simulations indicated that the greatest electric field enhancement occurred at the corners of the rhombic dodecahedra, highlighting that the degree of interaction between the analyte molecule and the different nanocrystals also significantly influence the SERS response. In another study, Song et al. synthesized self-assembled amphiphilic plasmonic hollow Au nanovesicles for cancer target and drug delivery applications **(Figure 9c)** 64. The nanovesicle possesses a PEG layer, a pH-responsive polymer coating, a Raman reporting molecule, and DOX as an anticancer drug **(Figure 9c)**. The self-assembled nanovesicle possesses an enhanced SERS response than the single AuNP due to the proximity of the hot spots. But under the physiological acidic conditions inside the cancer cells, the SERS response of the dye will be different due to the disassembly of the pH-sensitive Au nanovesicle, and this property has been taken as a measure for the cancer studies using SERS **(Figure 9d)**.

#### 2.2.2.2 DNA origami-based assembly

DNA-based assembly leverages the programmable and specific binding properties of DNA to organize nanoparticles into desired configurations. This method is particularly advantageous for the controlled formation of nanogaps. DNA origami, for instance, allows for the creation of complex three-dimensional structures by folding long strands of DNA into predetermined shapes 66-68. These configurations can be used as scaffolds for arranging metallic nanoparticles with precise interparticle spacing, forming stable nanogaps suitable for SERS applications.

**Figure 10:**
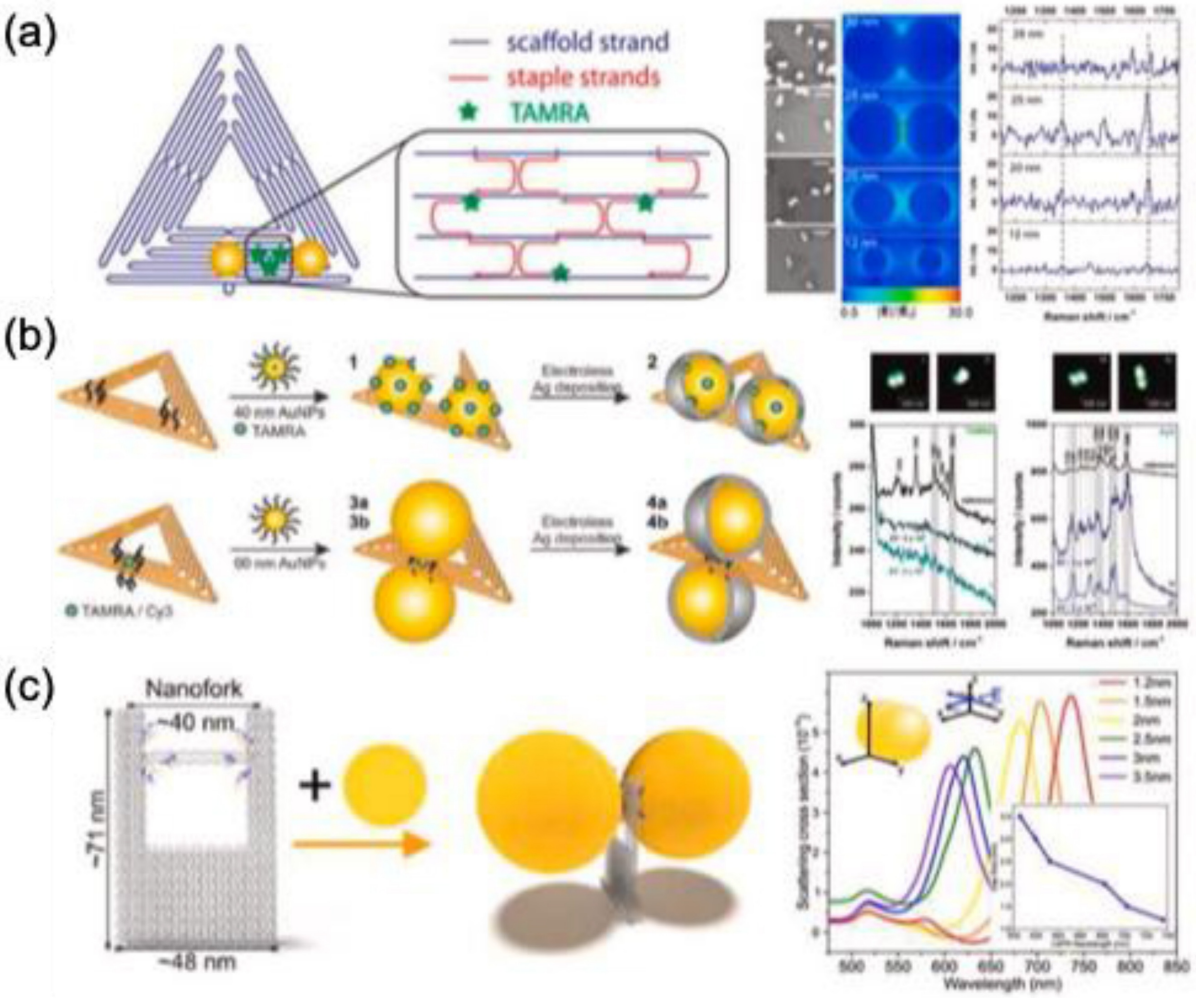
(a) Positioning of DNA-functionalized AuNPs on triangular DNA origami structures, (b) assembly of dimers on a triangular DNA origami platform using 40- or 60-nm AuNPs, followed by silver deposition (green dots represent fluorescent dye), and (c) nanofork-shaped DNA origami structure with Au dimer formation, where the green fluorescence in the nanogap signifies the presence of analytes. Reproduced with permission from ref 69,70,75. Copyright 2013, 2021, American Chemical Society and 2016, Royal Society of Chemistry.

J. Prinz et al. used triangular DNA origami to assemble AuNP dimers with tunable interparticle distances (5-28 nm), enabling fluorescence detection in the nanogap. The origami formed via hybridization of M13mp18 viral DNA with selected staple strands for analyte binding or DNA-modified AuNPs. Using 25-nm AuNPs yielded the highest SERS intensity for TAMRA **(Figure 10a)** 69. Further enhancement (up to 10¹⁰) was achieved by depositing an Ag layer, reducing interparticle distance to 14 nm for 40-nm AuNPs **(Figure 10b)** 70. Thacker et al. achieved a precise 3.3 ± 1 nm AuNP gap on 40 × 45 nm² DNA origami for detecting dye and short DNA molecules 71. Depending on dimer orientation, Raman enhancement ranged from five to seven orders of magnitude. This dimer strategy facilitates label-free detection of DNA binding sites and short DNA composition.

DNA origami's programmable properties make it a valuable tool for engineering plasmonic materials in SERS-based sensing. Heck et al. demonstrated that a silver nanolens assembly on triangular DNA origami enables streptavidin detection at hotspot regions 72. The structure utilized silver nanoparticles (AgNPs) of 10, 20, and 60 nm, with 10-nm AgNPs positioned at the center and a 3-nm intergap. A biotin-modified DNA strand facilitated strong streptavidin binding, while SERS monitoring was achieved through alkynyl-functionalized lysine, detecting Raman signals at 2100-2150 cm⁻¹. In another study, Tanwar et al. explored the self-assembly of Au nanostars on DNA origami for single-molecule detection of Texas Red 73. The nanostars (~70 nm, ~10-15 sharp tips) were arranged on rectangular DNA origami (90 × 60 nm) to form monomeric and dimeric nanoantennas with interparticle gaps of 7 and 13 nm. The complementary binding of thiolated oligonucleotides to the origami structure enabled Au nanostar assembly, generating an enhanced electromagnetic field for biomolecule detection with an enhancement factor (EF) of 2 × 10¹⁰. Additionally, bimetallic Ag-coated Au nanostar dimers (~70 nm, 2.5-nm shell) were organized on DNA origami with a 10 ± 1 nm interparticle gap for SERS-based pyocyanin detection 74. This setup achieved a detection limit of 335 pM, surpassing clinical requirements.

Tapio et al. developed a DNA origami nanofork antenna incorporating 60 nm AuNPs or AgNPs **(Figure 10c)** 75. The nanoparticles, coated with thiolated single-stranded DNA, hybridized with complementary DNA strands on the origami structure. The nanofork, measuring approximately 48 nm × 71 nm, featured two arms spaced ~31 nm apart, connected by DNA helices. With Ag:Au ratios of 3.5:1 and 2.3:1, the bridge facilitated precise analyte positioning for SERS analysis. The design enabled single-molecule detection of TAMRA, Cy3.5, and Cy5 with an enhancement factor of 10¹¹ and demonstrated excellent photostability in SERS studies of cytochrome c and horseradish peroxidase under nonresonance conditions.

#### 2.2.2.3. Nanosphere lithography-based assembly

Nanosphere lithography (NSL) leverages the self-assembly of colloidal nanospheres, commonly composed of polystyrene, to generate well-ordered structures that facilitate the formation of nanogaps 76. These nanogaps play a crucial role in SERS applications by significantly amplifying electromagnetic fields in confined regions. The core principle of NSL involves the precise deposition of metal onto the assembled nanospheres, followed by their removal, resulting in an array of nanoholes or nanogaps. These features effectively trap and concentrate light, thereby enhancing local electromagnetic fields. When molecules are positioned within these nanogaps, their Raman signals experience substantial amplification, primarily due to the "hot spots" formed in the narrow spaces between metallic nanostructures.

**Figure 11:**
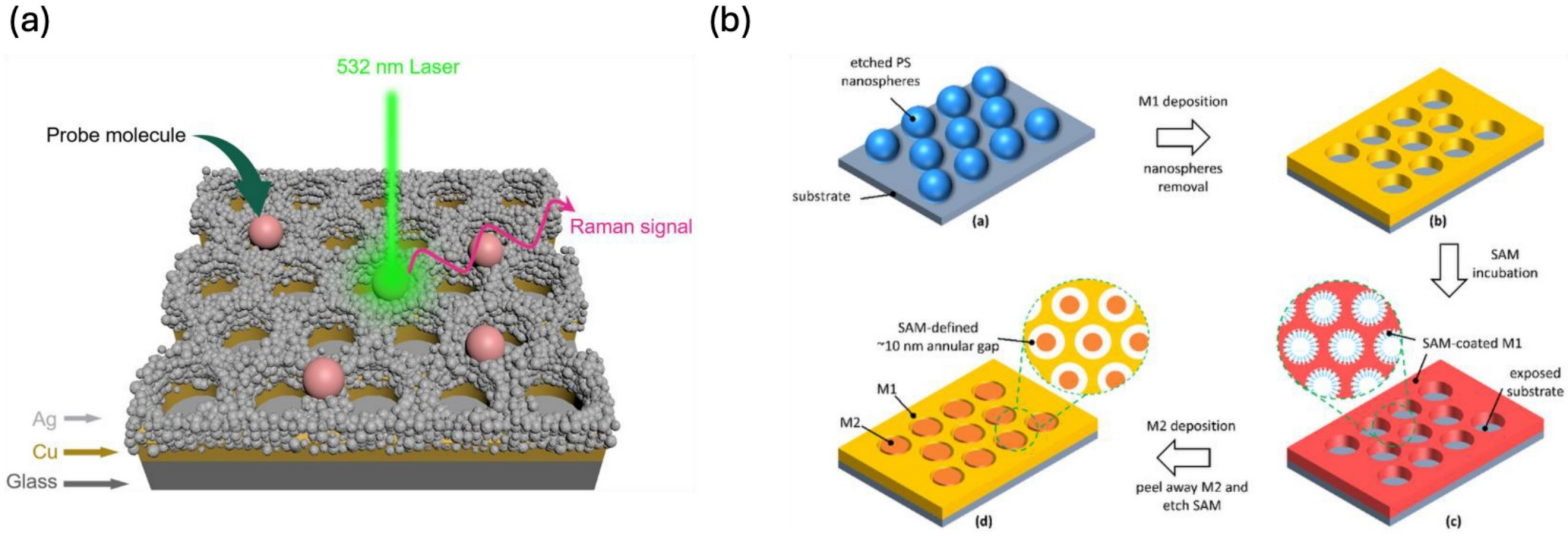
(a) Schematic representation for the hybrid Ag-Cu substarte prepared by NSL technique. (b) NSL technique for the fabrication of 10-nm Annular Gap Arrays for SERS applications. Reproduced with permission from 78,79. Copyright 2023, 2022, MDPI publishers.

S. Luo et al. recently developed large-scale parallel arrays with precisely tunable metallic nanogaps ranging from 3 to 30 nm 77. Their fabrication process involved first generating polystyrene nanoparticle arrays on substrates, followed by controlled size reduction through oxygen plasma etching. Subsequently, they introduced a self-assembled monolayer to facilitate the selective deposition of an additional metal layer. The final step involved peeling off the second metal layer, effectively revealing the well-defined nanogap pitch array. This method offers a scalable and reproducible approach for fabricating high-resolution nanostructures, which hold significant potential for applications in plasmonics, biosensing, and nanoelectronics. Recent research has introduced hybrid Ag-Cu SERS platforms, where silver nanoparticles are deposited onto a structured copper nanoarray, generating precisely engineered nanogaps with remarkable SERS enhancement **(Figure 11a)** 78. Additionally, an innovative approach integrating NSL with adhesion lithography has enabled the development of annular gap arrays with 10 nm spacing **(Figure 11b)** 79. These structures demonstrate exceptional reproducibility and SERS efficiency, achieving enhancement factors surpassing 10⁷ for the sensitive detection of Rhodamine 6G dye.

#### 2.2.2.4. Block polymer-based assembly

Block copolymer (BCP) based assembly allows fabrication of SERS substrates with uniform nanogaps. Block copolymers consist of at least two chemically distinct polymer segments that are covalently linked. Due to the inherent repulsion between these segments, they undergo spontaneous self-assembly into well-defined nanostructures under specific conditions such as solvent evaporation or temperature changes. This phase separation gives rise to ordered microdomains, forming structures like micelles, vesicles, or lamellar phases. In the context of SERS, the adaptability of these nanostructures enables the development of substrates with densely packed "hot spots," where localized electromagnetic fields are intensified, leading to enhanced Raman signal amplification from analytes.

**Figure 12:**
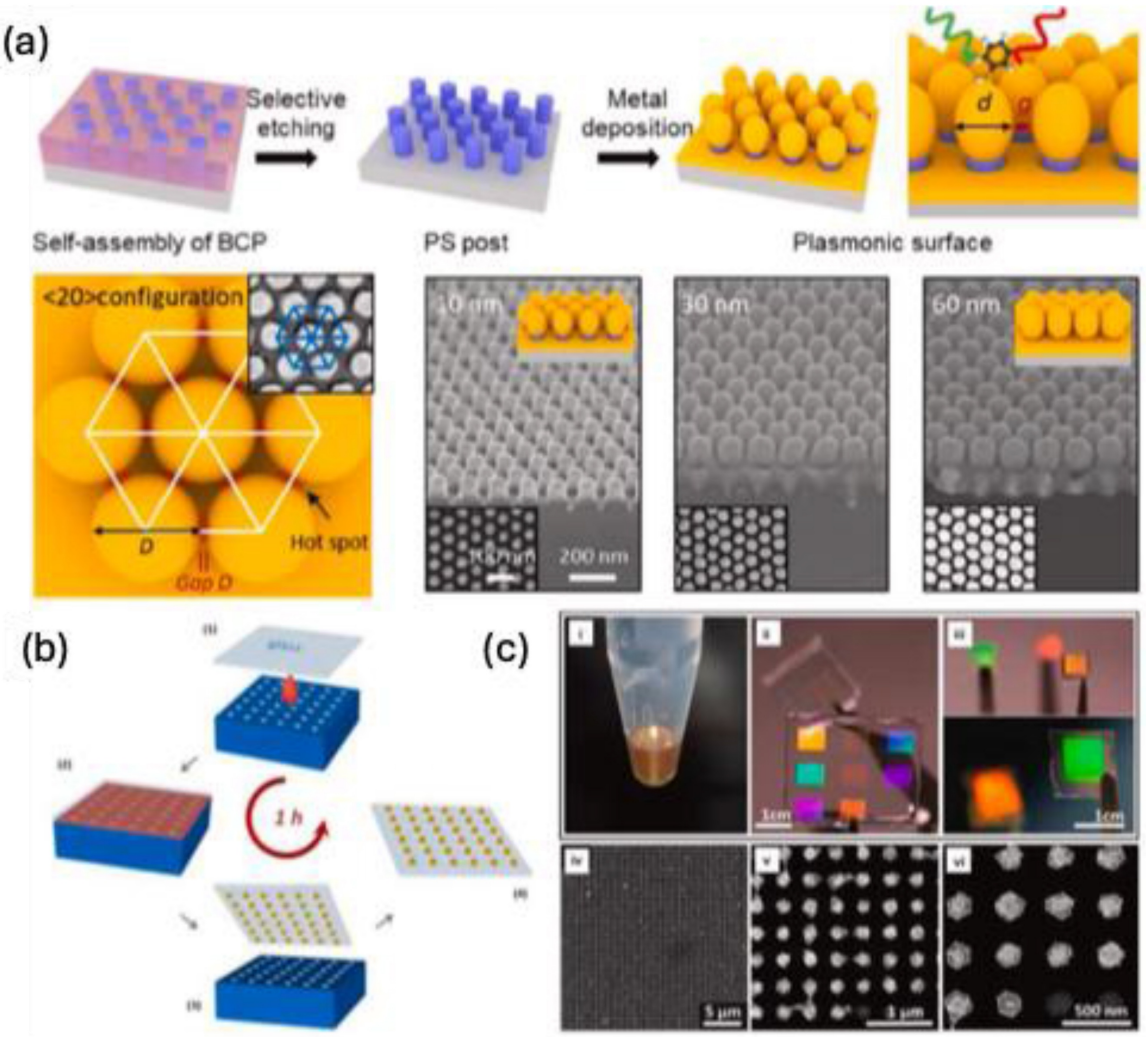
(A) A schematic representation and SEM images showcasing the formation of a hexagonal ultradense plasmonic array via PSNP assembly. (B) Diagram illustrating the sequential process of assembling Au nanospheres using a polydimethylsiloxane mold. (C) Top panel: images displaying (i) the dispersion of Au nanospheres, (ii) polymethyldioxane molds utilized for assembly, and (iii) the resulting Au nanosphere assemblies on a glass substrate. Bottom panel: SEM images depicting representative square lattice Au nanosphere clusters at varying magnifications (iv-vi). Reproduced with permission from 80,81. Copyright 2018 American Chemical Society.

Jin et al. demonstrated the use of BCPs combined with metal deposition to achieve a periodic arrangement of AuNPs with a 9.2 nm inter-nanogap, resulting in enhanced SERS properties 80. As illustrated in **Figure 12a**, a thin film of polystyrene-block-poly(methyl methacrylate) is spin-coated onto a silicon wafer functionalized with hydroxyl-terminated polystyrene-r-poly(methyl methacrylate). Exposure to tetrahydrofuran vapor induces the self-assembly of the block copolymer into a hexagonal array of polystyrene nanocylinders embedded in a polymethyl methacrylate matrix. Following plasma etching, gold deposition is carried out to create a plasmonic nanogap array. This well-ordered SERS platform exhibits high reproducibility and sensitivity in detecting biomolecules such as adenine. Matricardi et al. demonstrated that a hexagonally packed Au nanosphere superlattice can be created using a block polymer assembly technique with a polydimethylsiloxane mold 81. In their approach, a solution containing Au nanospheres is drop-cast onto a PDMS mold featuring a patterned square array of holes **(Figure 12b)**. The assembly process begins when a glass coverslip is placed on the polymer mold, allowing the colloidal solution to spread uniformly. The resulting two-dimensional superlattice exhibits strong optical properties in the near-infrared region, which can be adjusted from the near-infrared to the visible spectrum by modifying the lattice parameters **(Figure 12c)**. This superlattice achieves an interparticle spacing of less than 2 nm and demonstrates a threefold enhancement in SERS response for 4-acetamidothiophenol compared to noresonant random assemblies.

#### 2.2.3 Oblique Angle Deposition (OAD)

Oblique angle deposition (OAD) is a traditional physical vapor deposition technique, developed by Zhao and Dluhy et al., known for its effectiveness in creating high-quality SERS substrates through direct formation 82-84. In the OAD method, metal vapor atoms are directed onto a substrate at a steep angle (θ > 70 °) within a vacuum chamber, leading to the formation of metallic nanostructures, typically organized as arrays of nanorods or nanowires. Fu et al. introduced a modified OAD technique, termed localized OAD (LOAD), which incorporates a significant incidence angle to minimize the shadowing effect, allowing for the fabrication of silver nanorods on the inclined sidewalls of an etched substrate surface 85. These nanorods measured 573 ± 84 nm in length and 64 ± 12 nm in width on a silicon chip featuring distinct microcavity patterns with a pitch density of 5 μm. When the Ag nanorods created via LOAD were excited with a 785 nm laser, they demonstrated a remarkable Raman enhancement factor exceeding 3×10^5^ at 1282 and 1511 cm⁻¹ for Rhodamine B dye molecules. ​Due to their uniformity, reproducibility, and ability for large-scale production, OAD-fabricated substrates have become a cornerstone in research applications, particularly in the areas of bacterial sensing 86, virus detection 87,88, and microRNA analysis 89.

#### 2.2.4. Galvanic Displacement

**Figure 13:**
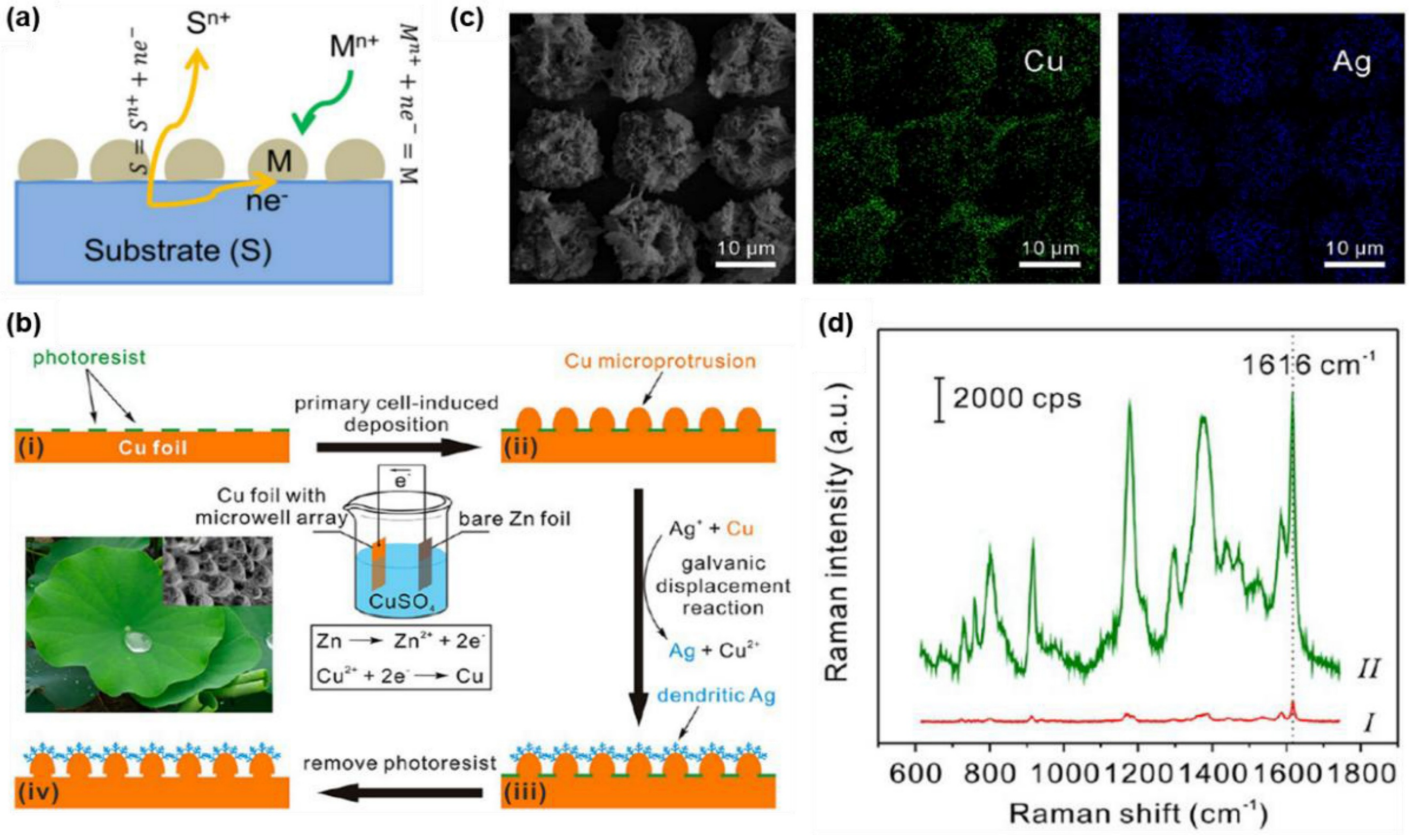
(a) A Schematic representation for galvanic displacement. (b) Scheme of hetero-hierarchical micro-nanostructure tetragonal array fabricated by galvanic displacement, (c) SEM and elemental mapping images of the fabricated structures, and (d) Raman spectra of crystal violet on a glass slide (red curve) and the fabricated surface (green curve). Reproduced with permission from ref 92,99. Copyright 2019 Creative Commons Attribution and 2013 American Chemical Society.

The galvanic displacement or replacement reaction represents a straightforward and cost-effective technique for creating controllable porous or hollow micro-nanostructures. This method exploits the redox potential of metals, enabling one metal to displace the atoms of another within a solution or on a solid surface to produce diverse nano-micro substrates **(Figure 13a)** 90-92. While it necessitates an external electrical source or electrodes, it can be executed under ambient conditions and within a short timeframe, making it a favored choice for fabricating SERS substrates 53,91,93. Numerous researchers have successfully developed plasmonic nanostructures, such as gold (Au) and silver (Ag), on various metallic or semiconducting substrates including aluminum 94, iron 95, silicon 96, germanium 93, and copper 97,98 for SERS applications.

Zhang et al. created dendritic tetragonal arrays of silver micro-nanostructures on copper foil, intended for use as superhydrophobic SERS substrates as illustrated in **Figure 13b, c** 99. The hierarchical architecture of these silver structures positively influenced their superhydrophobic properties, enabling them to function effectively as both concentrators and sensitive SERS substrates. Following the evaporation of droplets containing AuNPs and crystal violet dyes, the concentration of these components gradually rose. Ultimately, AuNPs and crystal violet coalesced at a single point on the substrates, leading to a notable enhancement in the Raman intensity of the dye compared to the negligible signal observed on a glass slide **(Figure 13d)**.

Shin et al. engineered track-engraved silver dendrites on a copper plate, followed by a self-assembled coating to create a superhydrophobic SERS platform 100. Analyte-containing droplets of Rhodamine 6G were intermittently injected through the track using a microneedle, allowing for real-time observation of SERS signals. They achieved a detection limit of Rhodamine 6G at a concentration of 10^-6^M, with an enhancement factor of 6.3×10^5^, comparable to conventional closed-channel microfluidic SERS devices. ​Nonetheless, substrates produced through galvanic displacement face challenges, such as the difficulty in precisely controlling the density and morphology of the structures, which can hinder the reproducibility of biomolecule detection 101.

**Advantages of Bottom-Up Methods**

- Controlled synthesis of NPs
- Creation of various shapes needed for hot-spot generation
- Tunable size
- Simpler fabrication methods
- Very sensitive

**Disadvantages of Bottom-Up Methods**

- Difficult to control the precise nanogaps
- Nanoparticle aggregation
- Time consuming for preparation of NPs
- Poor reproducibility
- Challenges for large scale uniform fabrication

### 3.1. Combined approaches for fabricating SERS substrates

By merging both bottom-up and top-down methodologies, it becomes feasible to produce cost-effective nanostructures with SERS capabilities. As an example, Ag nanopatterns were crafted using EBL and nano transfer printing 30. In this procedure, PDMS stamps featuring periodic arrays of square, triangular, and elliptical pillars were generated through EBL with a nanogap of 100 to 200 nm depending on the pattern. A modified cyclodextrin was thermally evaporated onto the stamp to counteract the adhesive properties of the EBL resist and serve as a release layer. Subsequently, Ag was physically deposited onto the stamp with precise control over the deposition rate and thickness and then employed directly for nanotransfer printing. The transferred Ag nanodisk-PDMS substrates were subsequently utilized for SERS investigations. Notably, post-transfer physical adjustments of the PDMS (e.g., inter-nanodisk spacing) could be employed to modify the morphology, and the stamps remained reusable after this process. The SERS response exhibited by the silver nanoparticles transferred onto the PDMS substrate underscores nanotransfer printing as a promising nanofabrication method, addressing the cost limitations associated with EBL, while enabling the creation of extended SERS substrates. In a separate study, researchers introduced a novel technique called nanotransfer edge printing. This approach combines nanoparticle self-assembly, nanotransfer printing, and edge lithography to create controllable SERS substrates. To elaborate, they meticulously arranged 20 nm Au nanoparticles by manipulating a topographically patterned PDMS stamp. Subsequently, they connected Ag nanoparticles to these patterns through thiol-metal bonding, forming SERS-active substrates. These substrates exhibited remarkable sensitivity, enabling the detection of minute quantities (10^-13^ mol/L) of biological molecules like thrombin with enhancements reaching up to 10^10^ 102. Additionally, a synergistic approach involving nanoimprint lithography, guided self-assembly, and self-alignment was employed to craft a dense 3D cavity nanoantenna array featuring closely spaced plasmonic nanodots 103. This developed substrate boasted a substantial area-average SERS enhancement of 1.2×10^9^ and demonstrated outstanding uniformity across the entire sample, with only a 22.4% variation. It is worth noting that the best uniformity achieved was a 15% variation over a 1.6 mm ×1.6 mm area, albeit at a slightly lower enhancement factor. This uniformity remained consistent regardless of the size of the excitation laser probe, ranging from approximately 1 to 10,000 μm. Lately, screen printing has found application in creating disposable SERS arrays for efficient high-throughput analysis. In this method, silver colloid was initially produced through a bottom-up wet chemical approach and blended with sodium carboxymethylcellulose to create the ink. This ink was subsequently applied to a supporting substrate using the screen-printing technique, enabling large-scale production without the need for costly equipment or complex procedures. These produced SERS arrays exhibited outstanding SERS performance, consistent reproducibility, and extended shelf life, and hold the promising potential to enhance the applicability of practical SERS analysis.

Recently, there has been a significant focus on utilizing graphene and its derivatives as substrates for metal nanoparticles to create SERS-active functional materials. These efforts involve both bottom-up and assembly-based approaches. For example, a hybrid SERS substrate composed of graphene and gold nano-pyramids was fabricated by Wang et al. 104. This innovative substrate employs a combination of techniques to enable the detection of individual molecules. They initially created a periodic structure of gold nano-pyramids with adjustable size and sharpness through a process involving colloidal lithography, etching, template adhesion, and peeling. Subsequently, they transferred a single-layer graphene sheet, produced using chemical vapor deposition onto the pyramid tips using PMMA. The graphene material naturally conformed to the underlying substrate geometry due to van der Waals forces. This resulting hybrid substrate created an abundance of hot spots with local SERS enhancement factors exceeding 10^10^ for molecules such as rhodamine 6G and lysozyme, facilitating the label-free detection of individual molecules.

## 4. Emerging Instrumentation techniques for SERS

### 4.1. Microfluidic system integrated with SERS

**Figure 14:**
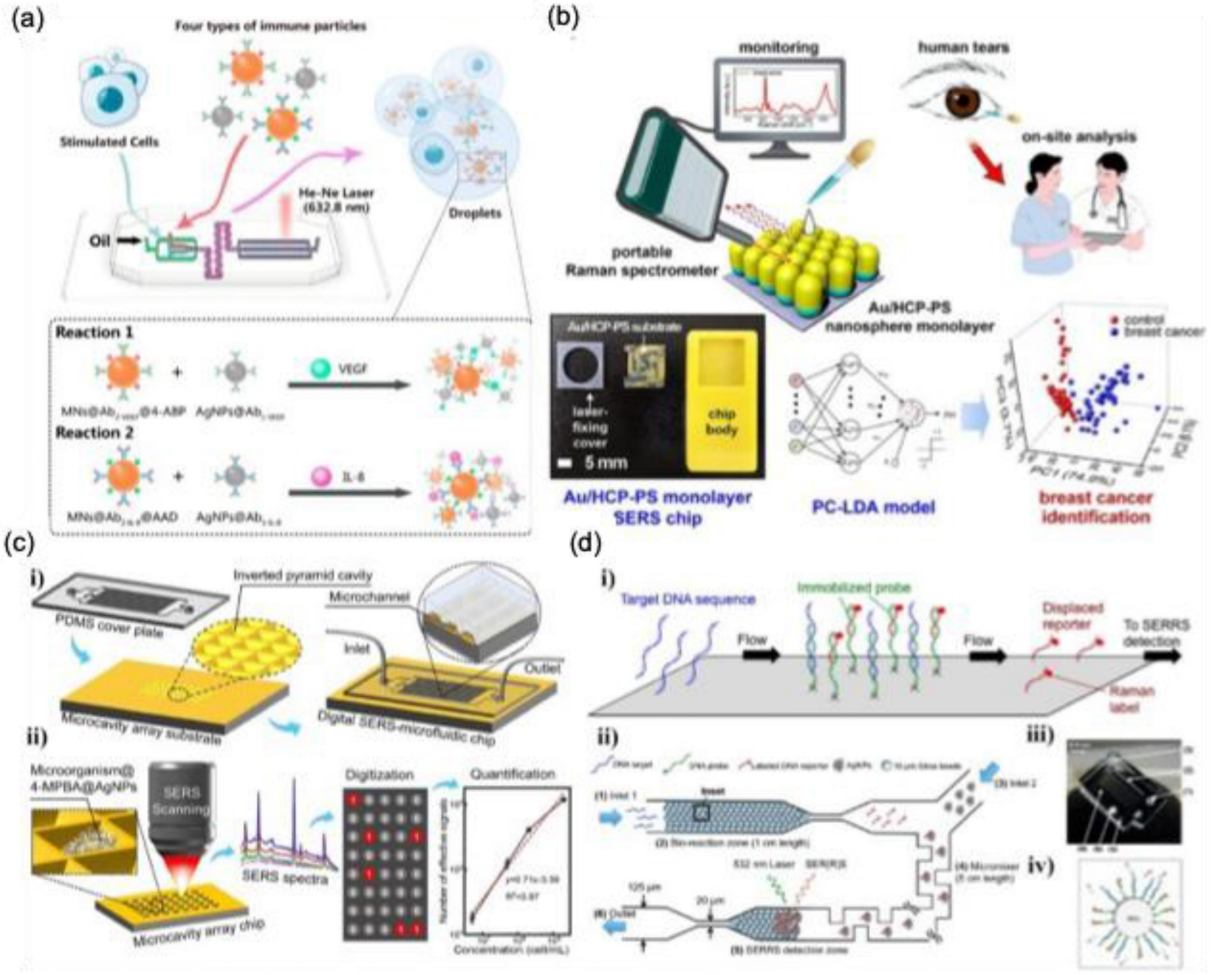
Microfluidic SERS-based biosensing platforms (a) Droplet-based microfluidics for single-cell encapsulation and SERS detection of VEGF and IL-8. (b) Working principle for Au/HCP-PS microchip for breast cancer biomarker detection. (c) (i) A representation for the digital SERS-microfluidic chip and (ii) SERS detection approach for microorganisms. (d) (i) A competitive displacement assay-based SERRS microfluidic platform for DNA sequence detection. (ii) A microfluidic SERRS microsystem with integrated competitive displacement for DNA sequence identification. Upon introducing the target sequence at the inlet, Raman-labeled reporter oligos are displaced. As they move through the channel, they interact with metal nanoclusters and are captured in the SERRS detection region of the microfluidic system. (iii) A photograph of the microfluidic SERRS microchip. iv) Silica microspheres functionalized with DNA probe-reporter pairs. Reproduced with permission from 107,111-113. Copyright 2019, 2024, 2020 and 2013, American Chemical Society.

Microfluidic technology has been widely utilized in sensor development, including diabetes and pregnancy test kits. The integration of SERS with microfluidics has further enhanced analytical precision while reducing costs and sample volume requirements 105,106. Microfluidic SERS platforms provide contamination-free environments, automated sampling, and high measurement accuracy, making them highly effective for biosensing applications. A key advantage of microfluidic SERS systems is their ability to bring SERS-active nanomaterials ("hotspots") close to analyte molecules, enabling sensitive detection even in complex biological, environmental, and food samples. This technology is particularly promising for cytokine detection, essential for understanding inflammatory disorders and autoimmune diseases. For instance, Sun et al. developed a SERS microfluidic droplet platform for the simultaneous detection of two cytokines, vascular endothelial growth factor (VEGF) and interleukin-8 (IL-8), secreted by a single cell 107. The incorporation of immune-MNs/AgNPs into the microdroplets significantly enhances detection sensitivity through metal plasmon amplification from adjacent AgNPs and magnetic field-induced aggregation as shown in **Figure 14a**. This highly sensitive platform facilitates large-scale analysis of cytokine heterogeneity at the single-cell level, providing crucial insights into their roles in tumor vascularization and aggressive growth.

Chip-based microfluidic sensors are gaining popularity due to their portability and rapid diagnostic capabilities, particularly in cancer biomarker detection 108-110.

Kim et al. introduced a label-free SERS biosensor using tear samples for breast cancer detection 111. They developed a microchip incorporating gold-decorated, hexagonal-close-packed polystyrene (Au/HCP-PS) nanosphere monolayer, leading to a remarkable giga-scale enhancement in signal intensity. **Figure 14b** illustrates the schematic representation of the SERS-based detection of cancer biomarkers from tear samples. The advancement of label-free microfluidic sensor chips utilizing SERS holds great promise for improving patient outcomes and increasing cancer survival rates. Furthermore, the synergy between microfluidics and SERS is being extensively explored for microbial detection across diverse applications, particularly in clinical diagnostics. Similarly, Wen et al. developed a digital SERS-microfluidic chip for rapid microbial detection, employing an inverted pyramid microcavity array to isolate and quantify microorganisms with high precision **(Figure 14c)** 112. These advancements underscore the potential of microfluidic SERS platforms in diagnostics, offering real-time, accurate detection for biomedical and clinical applications.

Recent research is exploring the heightened sensitivity of microfluidic SERS platforms for the ultra-sensitive detection of low-concentration DNA. These platforms offer unique advantages, such as multiplexing capability, high specificity, minimal sample volume requirements, and portability, making them highly suitable for a wide range of biological, medical, and environmental applications. A notable study by the Yazdi group demonstrated a similar approach by developing a competitive displacement assay microfluidic SERRS-based device for multiplexed DNA sequence screening 113. In this assay, the target DNA sequence displaces a Raman-labeled reporter sequence with lower binding affinity to the fixed probe, as illustrated in **Figure 14d**. This displacement mechanism enables a simplified, one-step detection process for unlabeled target DNA sequences.

### 4.2. Optical Tweezers

**Figure 15:**
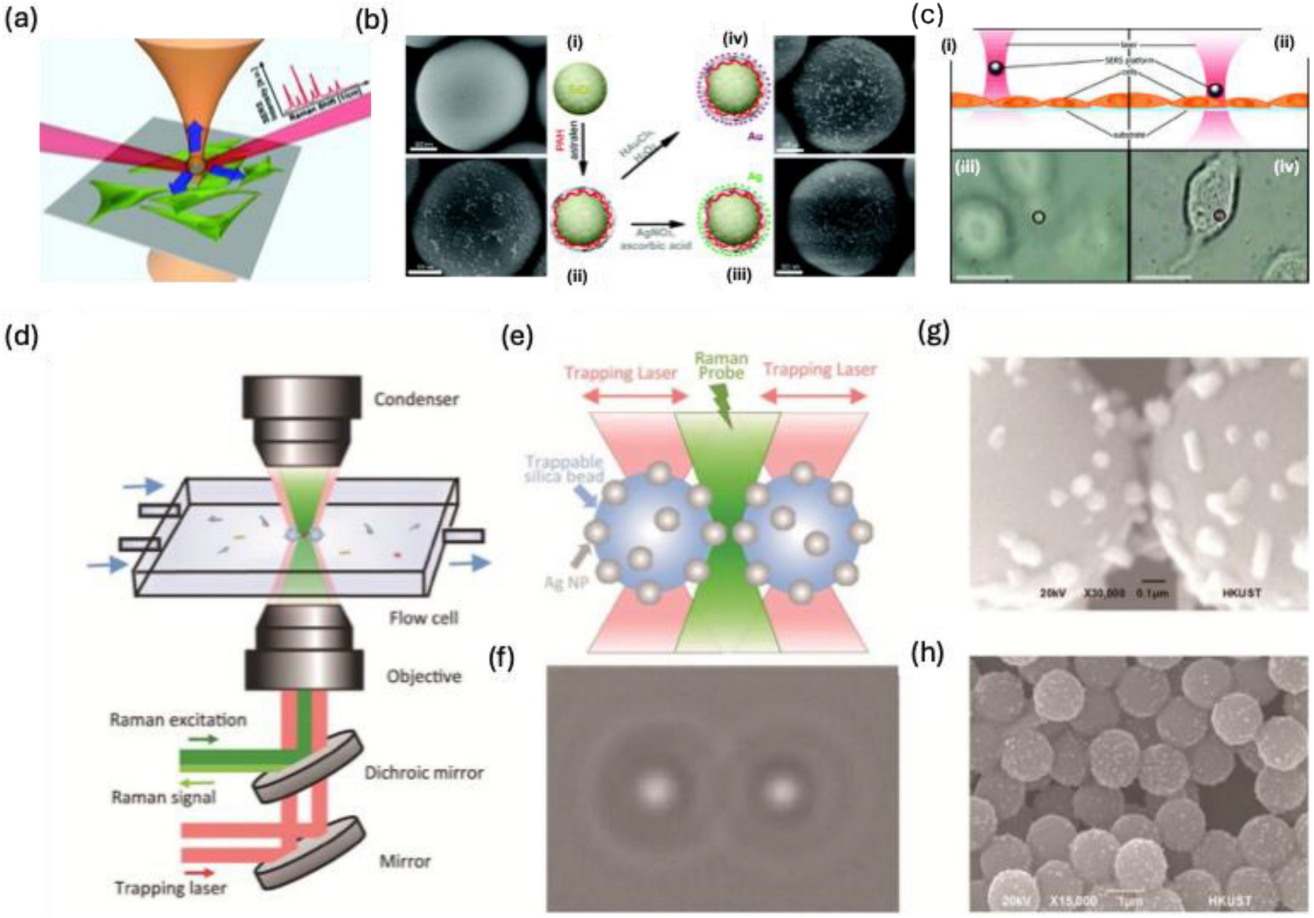
Optical tweezers for SERS application. (a) A schematic representation for the SERS-based satellites for the cellular component detection. (b) The fabrication process of SERS-active satellite structures, along with corresponding SEM images at each stage: (i) SiO₂ microspheres, (ii) SiO₂ coated with three layers of PAH/Astralen, (iii) SiO₂/(PAH/Astralen)₃ with Ag deposition, and (iv) SiO₂/(PAH/Astralen)₃ with Au deposition. (c) Schematics illustrating the laser tweezer-guided transport of SERS-active satellites to the cell surface (i, ii). Optical transmission images depict a SERS-active satellite captured by laser tweezers above an L929 mouse fibroblast cell (iii) and near the cell membrane (iv). Scale bars in the optical images represent 10 μm. (d) A schematic representation of an optical tweezer-integrated Raman spectroscopy system utilizing a microfluidic setup. (e) A diagram depicting two trapping laser beams controlling the spacing between AgNPs (red) and the Raman probe beam (green) for signal acquisition. (f) A real-time camera capture of AgNPs highlighting the interparticle gap. (g-h) SEM images of silica microbeads coated with AgNPs, demonstrating a consistent intergap and uniform nanoparticle distribution. The scale bar represents 1 μm. Reproduced with permission from 121, 2015 RSC publishing group; 114, 2021, Nature publishers.

Optical trapping techniques have been effectively combined with microfluidic systems to mitigate Brownian motion and precisely control the positioning of micro- and nanoscale objects suspended in solution 114,115. This integration enhances the efficiency and consistency of SERS measurements for various biomedical applications. Optical tweezers, also called optical traps, employ highly focused laser beams to manipulate a range of entities—including cells, microparticles, nanoparticles, molecules, and atoms—with exceptional spatial and temporal precision 116. The fundamental mechanism is based on the momentum transfer of light, wherein a tightly focused laser beam generates an intensity gradient that interacts with the object through absorption, reflection, refraction, or scattering. This interaction produces forces that draw the object toward the laser's focal point. By dynamically adjusting the beam's focus, researchers can reposition trapped objects without direct physical contact 117. This capability allows for the precise placement of target analytes near SERS-active surfaces, enhancing signal detection and acquisition. For microscale object manipulation, McNay et al. designed a partially silver-coated silica microparticle that serves as an optically trappable, microscopically visible SERS probe 115. Building on this approach, Balint et al. refined the design to enable spatial SERS scanning for cell membrane analysis 118. Stetciura et al., as depicted in **Figure 15a**, functionalized silica microparticles with metal NPs, forming SERS-active satellite structures that were directed into specific cellular compartments for single-cell analysis. To achieve this, a thin multilayer coating composed of (poly(allylamine hydrochloride)/astralen) ₃, abbreviated as (PAH/Astralen)₃, was applied onto solid silica microparticles **(Figure 15b)**. Astralen was incorporated as a detectable marker for Raman spectroscopy analysis, while PAH functioned as a binding agent, facilitating the attachment of astralen through the layer-by-layer technique. Three bilayers were selected to ensure an adequate presence of astralen molecules on each microparticle's surface. Following this, the particles were functionalized with metal nanoparticles such as Au and Ag NPs to enhance the Raman signal. The deposition of Au or Ag was achieved through established chemical reduction methods. As a result, the final core-shell microparticle structure was formulated as silica microparticles/(PAH/astralen)₃/Au(or Ag). Afterwards the ability of optical tweezers to trap engineered microparticles using a continuous-wave diode infrared laser was evaluated. Both silver- and gold-coated satellites were successfully trapped, but gold-coated satellites demonstrated greater stability, as silver-coated particles frequently escaped. This difference is likely due to the lower chemical stability of silver compared to gold. Consequently, the gold-coated silica-based platform provided more stable laser trapping, as illustrated in **Figure 15c (i)-(ii**). In a cellular experiment, laser-induced movement of the trapped satellites enabled clear visualization of the cell **(Figure 15c (iii-iv))**. For nanoscale manipulation, Svedberg et al. demonstrated the assembly of two silver NPs into a dimer, generating a dynamic hotspot that significantly amplified SERS intensity using optical tweezers 119. Similarly, Tong et al. leveraged optical aggregation of multiple metal NPs, forming interparticle hotspots that enhanced the SERS signal of adsorbed analytes 120.

Dai et al. developed an optical tweezer-Raman spectroscopy technique to analyze protein structures in hemoglobin, lysozyme, and bovine serum albumin **(Figure 15d)** 114. A 1064-nm laser is split into two beams, while a 532-nm Raman probe beam is focused inside a microfluidic sample cell **(Figure 15e)**. The probe irradiates the gap between two AgNP-coated trapped beads, manipulated in three dimensions by the laser beams **(Figure 15f)**. Real-time imaging **(Figure 15h)** and SEM analysis **(Figure 15g-h)** reveal interparticle distance and Ag coating on silica beads. This SERS platform enables precise protein detection in a controlled microfluidic environment, capturing transient alpha-synuclein structures via Raman spectroscopy.

### 4.3. Nanofingers

**Figure 16:**
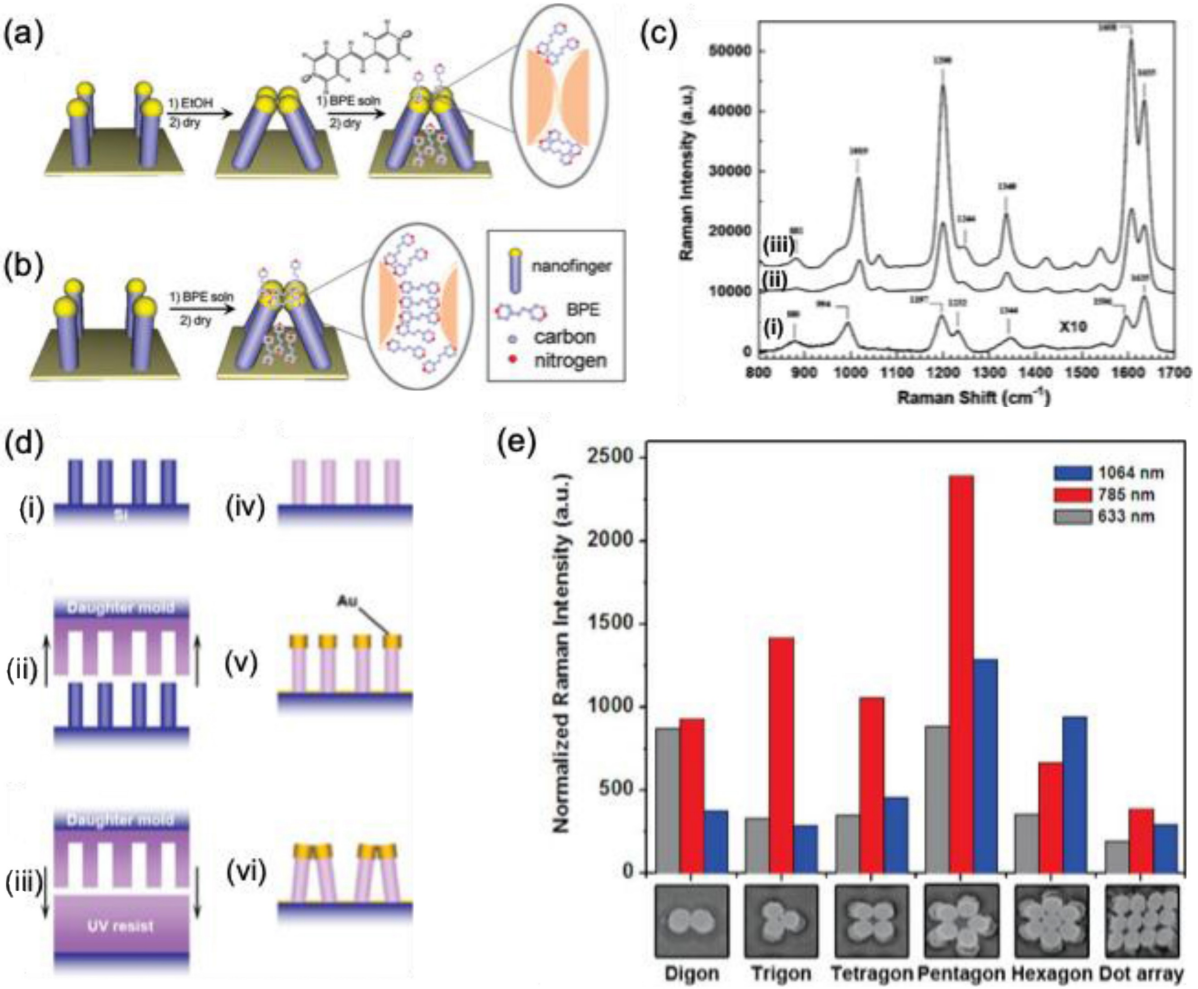
Nanofingers for SERS applications. (a) Schematic representation for the gold nanofingers and its working principle. The inset shows the magnified nanofingers which is trapping trans-1,2-Bis(4-pyridyl)-ethylene. (c) Raman spectra for (i) powder trans-1,2-Bis(4-pyridyl)-ethylene (ii) pre-closed and (iii) trapped nanofingers with trans-1,2-Bis(4-pyridyl)-ethylene. (d) Fabrication Process for Nanofingers where (i) Fabrication of the silicon nanofinger mold via e-beam lithography. (ii) Creation of the daughter mold through nanoimprinting. (iii, iv) Formation of polymer nanofingers from the polymer-based daughter mold using nanoimprinting. (v) E-beam deposition of 80 nm of gold onto the nanofingers. (vi) Solvent immersion and drying to facilitate the closure of the nanofingers. (e) Normalized Raman signal intensity for trans-1,2-Bis(4-pyridyl)-ethylene with various nanofinger structures. Reproduced with permission from 122,123. Copyright 2011, American Chemical Society.

Nanoimprinting technology enables the fabrication of gold-coated nanoscale polymer fingers on a polymer pillar array. Due to their flexibility, these nanofingers can bend, allowing the gold tips to come into proximity for effective molecular trapping 43. Since the initial study by M. Hu et al. in 2010, various target molecules have been detected, and different geometrical configurations have been developed. A comparable approach has been employed to capture trans-1,2-Bis(4-pyridyl)-ethylene within the gap formed during the closing motion of gold nanofingers 122 **(Figure 16a-b)**. The pyridine's lone electron pair interacts with tetrameric gold nanofingers, becoming confined within the "hot spot," leading to SERS signal that is three times stronger than that of fully closed nanofingers **(Figure 16c)**.

F.S. Ou et al. developed polygonal metallic nanostructures through self-assembly on flexible polymer pillars 123. These structures, including digon, trigon, tetragon, pentagon, and hexagon arrays made from AuNPs (136 nm diameter), can self-coalesce under microcapillary forces **(Figure 16d)**. SERS studies on trans-1,2-Bis(4-pyridyl)-ethylene in the hot spots of these nanostructures showed an enhancement factor of ~10^11 for the pentagon-type nanofingers, significantly outperforming the other polygons **(Figure 16e)**. The local symmetry of the nanofingers was found to influence the plasmonic properties. However, the use of polymer templates restricted the applications of these nanostructures. To overcome this, Barcelo et al. used nanoimprinting to fabricate metal nanofingers from Au or Ag by evaporating metal films on flexible polymer pillars 124. The metal caps formed on the pillars could be pulled and assembled into the desired architecture, and nanoparticle assemblies were transferred to silicon, glass, or metal-coated substrates. These nanofingers, with an average size of <2 nm, demonstrated a SERS enhancement factor of 2-5 × 10^8 for trans-1,2-Bis(4-pyridyl)-ethylene. Additionally, Kim et al. employed Au nanofingers for melamine detection in milk using portable Raman spectroscopy 125. Pentamer Au nanofinger chips, after being dipped in melamine solution and air-dried, were rinsed with ethanol to close the nanofingers. The trapped melamine was analyzed using Raman spectroscopy, offering detection limits of 120 ppt in water and 100 ppb in infant formula.

### 4.4. Dielectrophoretic Trapping strategy for SERS

**Figure 17:**
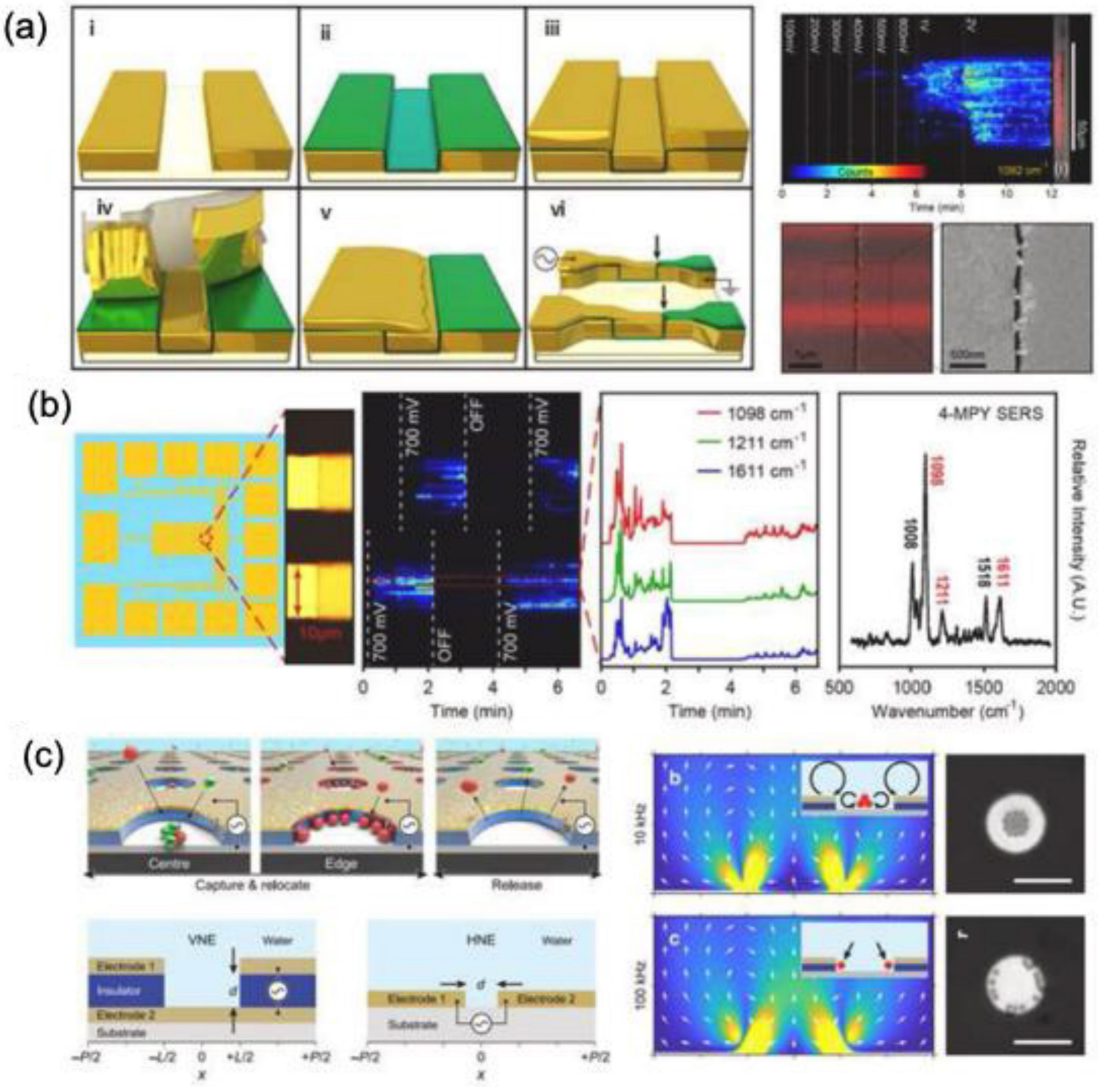
(a) Schematic illustration and demonstration of the fabrication process for the dielectrophoretic SERS platform. (b) SERS signal profile and frequency response of 4-mercaptopyridine encapsulated within liposomes. (c) Visual depiction of the capture and controlled relocation of biological nanoparticles using the dielectrophoretic approach. Reproduced with permission from 126,127, Copyright 2018 American Chemical Society, 2020 Nature publishing group.

Dielectrophoretic trapping captures analytes without chemically altering or bonding them. This method employs two electrodes in a solution with alternating electric fields, inducing dipoles in particles that move based on the field gradient. The direction of movement depends on the oscillation frequency and the dielectric properties of both the particles and the medium. Its simplicity and scalability make it widely used for manipulating cells, vesicles, and biomolecules 126,127. Ertsgaard et al. demonstrated a dielectrophoretic trapping technique using AuNPs and a lineated illumination scheme for real-time SERS imaging of liposomes 127 **(Figure 17a)**. The fabrication process begins with defining the gold electrode through photolithography (Step I), followed by the deposition of an 11-nm-thick Al₂O₃ film to establish the gap (Step II). In Step III, a nonconformal evaporated gold layer is applied, forming the second electrode edge. The excess gold is then removed using an adhesive peel (Step IV). In Step V, gold is sputtered onto one side of the trench to create an electrical connection between the electrodes. Finally, a photolithography step (Step VI) defines the trap sites and electrode pads for experimental use. Alternating electric fields induced dipole interactions, guiding dielectric particles based on frequency and medium permittivity. Liposomes, composed of 1,2-dimyristoyl-sn-glycero-3-phosphocholine and cholesterol, were trapped between AuNPs capped with 4-mercapto pyridine, producing a SERS signal. This technique differentiates vesicles by size and composition, with SERS signals detected at 1 and 10 MHz, but liposome-specific signals appearing only at 1 MHz **(Figure 17b)**.

Yu et al. conducted a study in which they developed a vertical nanogap electrode to capture and reposition lipid vesicles and peptide assemblies at the nanometer scale **(Figure 17c)** 126. The electrode pattern was strategically designed to regulate flow dynamics, facilitating the trapping of particles between the electrodes. Both experimental and theoretical analyses confirmed that the vertical nanogap electrode effectively captured bio-nanoparticles smaller than 100 nm when subjected to a low voltage **(Figure 17d)**. This approach enabled the selective capture of nanometer-scale lipid vesicles and peptide assemblies based on their size by applying an appropriate potential. In another study Szymborski et al. reported SERS sensor for the detection of circulating cancer cells using the dielectrophoretic effect 128. The choice of alternating field and its shape allowed deposition of cancer cells on microfluidic system. The SERS analysis for MCF-7 and MDA-MB-231 cells showed a detection limit of 20 cells/mL with a potential clinical application of this platform.

## 5. Nano-micro fabricated sensors for Bioanalysis using SERS

In the early stages, metal nanoparticle aggregates were the most effective materials for Surface-Enhanced Raman Spectroscopy (SERS) due to their enhanced light transmission and interactions with a wide range of analytes. However, the practical application of these colloidal nanoaggregates is limited by their poor temporal stability, lasting only hours or days. As discussed earlier, 3D nano-microstructures address this limitation by offering more stable and highly ordered "hot spots" that exhibit improved SERS activity. These 3D structures have a wide range of potential applications, but in this review, we focus on their use in bioanalysis. The selective detection of biomolecules with high sensitivity and reproducibility is crucial for disease diagnosis and treatment. While Raman spectroscopy has been widely reported as a bioanalytical tool, challenges related to substrate sensitivity have led to the development of 3D nano-microfabricated SERS structures, which provide reproducible and accurately calibrated results. Additionally, the structural design and morphology of these 3D platforms play a key role in enhancing SERS signals. Both experimental and theoretical studies have demonstrated that significant electromagnetic field enhancement can occur between adjacent nanomaterials and arrays of anisotropic metal nanoparticles on 3D surfaces 2,52,129. This review will highlight recent advances in anisotropic 3D metal structures and their application in various bioanalytical context.

### 5.1 Detection of DNA

The current detection of DNA is based on polymerase chain reaction (PCR), which requires amplification for fluorescence detection. DNA does not show fluorescence; however, fluorescence tags are required to show fluorescence. However, the major issue with such methods is the photobleaching of the fluorescent dye and the overlap of most of the emission peaks, which lacks sensitivity for multiplexed DNA detection.

Park et al. reported an alligator teeth-type polydimethylsiloxane microfluidic channel embedded with Ag colloids of 65-70 nm size for the confocal SERS measurement of DNA duplex oligomer mixtures 130. This method does not require the PCR amplification and immobilization criteria, unlike the other methods for DNA detection. Two different dyes labeled (Cy3 and TAMRA) at the 5' end of sex-determining Region Y gene (SRY) and SPGY1 were analyzed using this PDMS microfluidic channel by con-focal SERS measurement. Since PDMS is a Raman active polymer, the confocal SERS measurement removed the Raman signals of PDMS and allowed the DNA detection down to a concentration of 10^-11^ M. This lab-on-chip method can be applied to non-fluorescent dyes and can be used for sensitive bio-analysis and microenvironment analysis.

**Figure 18:**
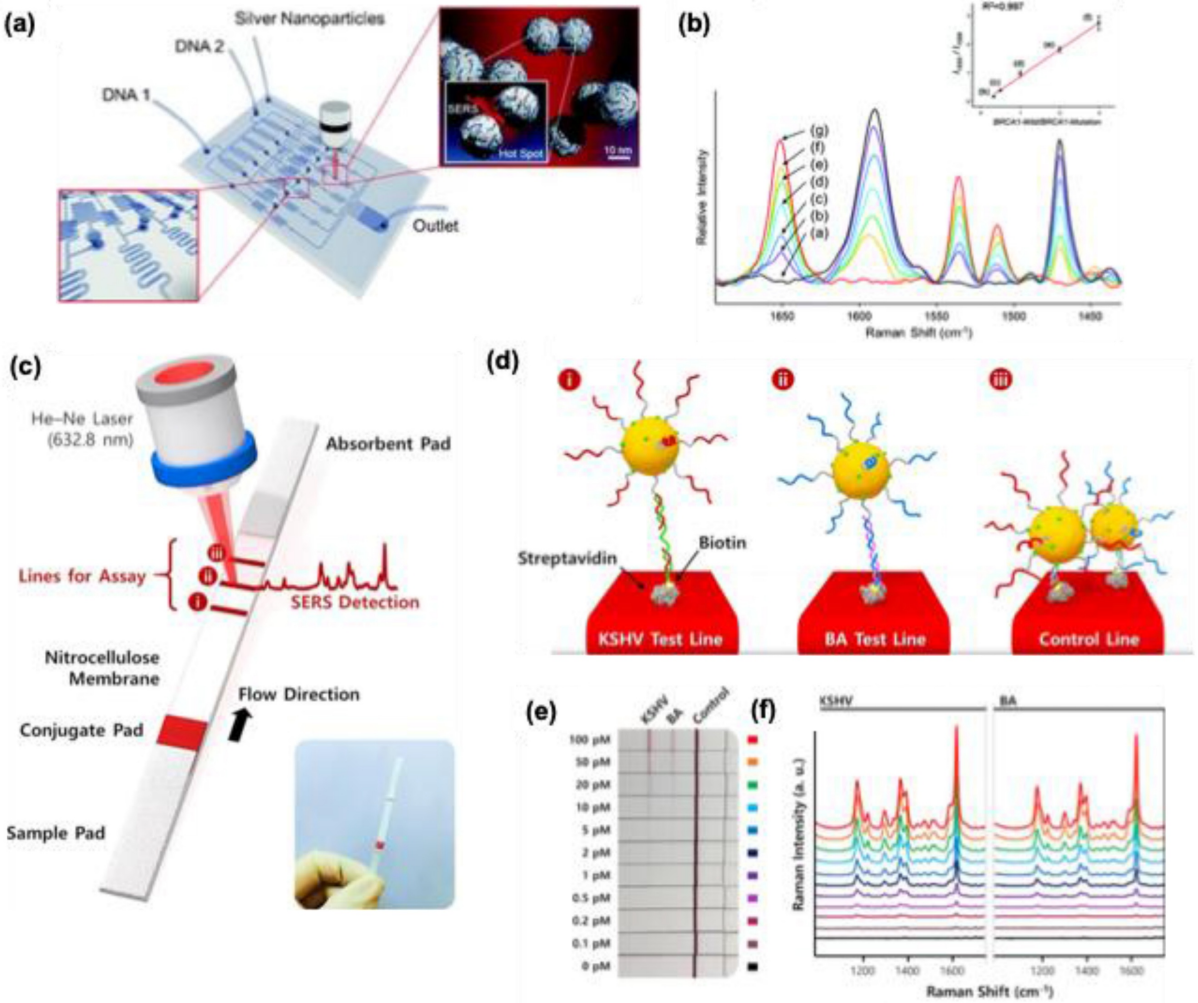
(a) A schematic of the programmable SERS-based microfluidic channel for DNA mixture detection. (b) SERS confocal spectra of duplex DNA oligomer mixtures were obtained at varying molar ratios of TAMRA-labeled BRCA1-Wild to Cy3-labeled BRCA1-Mutation: (a) 0:1, (b) 1:3, (c) 1:2, (d) 1:1, (e) 2:1, (f) 3:1, and (g) 1:0. The inset shows the change in peak area ratio (I1650/I1588) relative to the BRCA1 ratios. ​(c) A schematic of the LFA biosensor, highlighting its ability to detect two nucleic acids simultaneously, features two test lines and one control line.​ (d) (i) KSHV DNA and gold nanoparticles (Au NPs) are captured by KSHV DNA probes on the first test line; (ii) BA DNA and Au NPs are captured on the second test line; (iii) Excess KSHV and BA detection DNAs attached to Au NPs bind control DNAs on the third line via T20-A20 hybridization. (e) Digital images show the strip's visual recognition capabilities, and (f) the Raman spectra for varying concentrations of KSHV and BA DNAs (0 to 100 pM) are presented. Reproduced with permission from ref 131,136. Copyright 2012 Royal Society of Chemistry and 2017 American Chemical Society.

PCR and micro-array-based DNA chip techniques require a large hybridization time, and during this operational time, the chances for DNA contamination are higher, so the urge for new platforms for DNA detection with a shorter time than PCR is required. Recently Chio et al. developed a programmable SERS-based microfluidic channel for the detection of DNA mixtures **(Figure 18a)** 131. The fabricated microfluidic channel system possesses three compartments, in the first compartment different concentration gradients of DNA targets can be injected, and in the second compartment, the Ag nanoparticles (45-55 nm) are distributed evenly over the SERS detection channel **(Figure 18a)**. Cy3-labeled BRAC1-Mutation DNA oligomer and TAMRA-labeled BRAC1-Wild DNA oligomer were used for the studies. Due to the negative charge on both AgNPs and DNA backbone, spermine tetrahydrochloride was utilized to neutralize the charge on the Ag NPs for an effective adsorption process. The whole detection process is finished in less than 10 minutes without PCR amplification. The intensities for TAMRA at 1650 cm^-1^ and Cy3 peak at 1588 cm^-1^ are monitored to calculate the molar ratio of the DNA oligomers and studies showed a good linear relation for a wide concentration range **(Figure 18b)**. The advantage of the system is that there is no amplification steps needed to differentiate the DNA from the mixtures and the whole experiment was performed while the DNA mixtures are flowing inside the chamber. So, avoiding such immobilization steps for DNA detection using SERS can be used as a powerful tool for multiple DNA target detection.

Label-free detection of DNA is more advisable and accurate since in these kinds of SERS techniques the DNA can be detected directly without any labeling agent. Ngo et al. fabricated a molecular sentinel-type platform for the detection of the RSAD2 gene within a short time and without further purification 132. Polystyrene beads (520 nm size) were first coated with 200 nm of gold and a molecular sentinel was attached to these nano wave chips. The molecular sentinel is characterized by its hairpin loop-like structure with a Raman reporting molecule (Cy3 Raman dye), and during its normal configuration, it possesses a strong Raman signal due to its proximity to the metal nanoparticles. The nano wave chip possesses ∼6 × 104 enhancement in the SERS response due to the peculiar fabrication pattern. The present analysis is based on the configurational change of the molecular sentinel after the hybridization with the target DNA. The Raman signal intensity of the molecular sentinel at 1197 cm^-1^ is taken as a measure and the intensity will decrease due to the separation of the Raman dyes from the proximity of the metal nanoparticles after the hybridization with the target DNA. This study offers a label-free detection of DNA compared to the other techniques with a minimum of 20 aM sensitivity.

Lateral flow assay chips (LFA) are economical and user-friendly 133. Even though they have been used for several bio applications, they lack sensitivity and limited quantification capability. The incorporation of SERS substrates into the LFA chips remarkably enhanced their sensitivity and rapid detection capability 134,135. Wang et al. reported an LFA biosensor for the multiple DNA target detection for Kaposi's sar-coma-associated herpesvirus (KSHV) DNA and bacillary angiomatosis (BA) DNA genes visually and by SERS studies **(Figure 18c-f)** 136. The lateral flow strip is divided into four chambers, and the first chamber is the sample pad which is blocked using Tris-HCl (20 mM), Triton (0.25%) band 150 mM HCl for the optimum analysis of the targets. SERS nanotags were made as a conjugate pad, which contains AuNPs functionalized with Malachite green isothiocyanate as a Raman reporter and AuNPs were further attached to the thiol DNA targets. The nitrocellulose pad which is the third chamber was used to attach streptavidin-biotinylated capture probes for KSHV and BA which form two test lines and one control line. The fourth chamber which is an absorption pad helps for the continuous flow of the analyte via capillary action **(Figure 18d)**. Finally, all these pads were assembled using a plastic adhesive for practical application. Using the streptavidin-biotin interaction, both KSHV, and BA were immobilized on the cellulose membrane, and a 1:1 mixture of DNA-SERS tags was adsorbed on the conjugate pad. Due to the capillary action when the target sample solution is put on the sample pad, it will migrate to the absorption pad and reach the conjugate pad, the targets will get hybridized with the DNA-AuNPs tags, to form two different DNA hybrid complexes **(Figure 18e)**. As shown in **Figure 18e**, from the digital photograph it's difficult to identify the KSHV and BA by the colorimetric response for concentrations less than 10 pM. Combining the later flow chip with the SERS platform enabled the quantification and detection of the two DNA targets by monitoring the Raman signal intensity at 1617 cm^-1^ **(Figure 18f)**. This method offers a low detection limit for KSHV (0.043 pM) and for BA DNAs (0.074 pM), which is 10,000 times more sensitive than the other methods.

Recently, D-K Lim's research group, demonstrated that using magnetic microparticle-based assay detection of DNA can be performed accurately using fluorescence and Raman scattering optical methods 59,137,138. The detection method offers a simple and reproducible assay for the target DNA detection. The magnetic microparticles are first functionalized with the capture probe DNA which is half complementary to the target DNA and half complimentary to the signal DNA bearing a Raman reporting molecule (ATTO 448, ATTO 565, ATTO 647N). The working principle is the hybridization of target and signal DNA probes with the magnetic microparticles capture DNA probe and after incubation for 3 h, using melting temperature as a stimulus the release of signal and target DNA is achieved.

The use of magnetic microparticles is that they can easily separate using a magnet in both the hybridized and release stages of the DNA to avoid contaminations. From the amount of the released signal probes using the two-detection techniques like fluorescence and Raman spectra, we can quantify the DNA targets. The magnetic microparticles-based assay using AuNPs exhibited a high sensitivity of approximately 30 fM LOD for Raman analysis, which is superior to the fluorescence analysis (~pM) for E. coli genome DNA 137. The researchers also carried out multiplexing assays for three different bacterial target DNA (E. coli, E. faecalis, and S. aureus), showing the multiplex-ing capability by SERS-based analysis. In another study, they demonstrated a similar strategy can be used for the (RdRp), (E), and (N) genes detection in SARS-CoV-2 using the SERS platform. The study demonstrated that using AgNPs and suitable aggregating agents, viral DNA detection can be achieved with a concentration of 1.0 fM. Moreover, the study further highlighted the use of anisotropic AgNPs like triangular nanoplates and nanostars for the improved detection of RdRp gene target to attomolar level (10 aM). The present study avoids the amplification steps which are commonly used in the PCR method and showed high reproducibility in the solution as well as in the dry state analysis 59.

The similar group showed the use of gold-silver core-shell nanodumbbells for the detection of bacterial genomes in a multi-well array platform using SERS 138. AuNPs with size 20 nm (i.e. probe A) and 30 nm (i.e. probe B bearing Raman reporting molecule Dabcyl were functionalized with the thiolate DNA probes and hybridized with the target DNA to form the AuNPs dimer and for better Raman response Ag shell of 15 nm thickness were introduced to the probe. The studies showed that the multi-well array assay can achieve a high sensitivity of 1.0 aM with reproducibility for the S. aureus, and E. faecalis bacterial genes. The study further highlighted the importance of the length of the dimer formation and the need for Ag shell for the Raman response. An optimum of 15 nm Ag shell thickness is crucial for the Raman response and the method can be used for the detection of other pathogens with high accuracy and reproducibility. Even though SERS platforms can be used for accurate DNA detection, the stability of the developed platforms or the devices is a challenging field. Moreover, the developed platforms should reach up to the biological issues in a point-care detection platform. Another issue is the suitable choice of Raman reporting molecules for the bioassay and their signal-to-noise ratio value for the vibrational modes. The cost-effectiveness for the fabrication of such SERS platforms with accuracy and reproducibility is also an issue for future applications.

### 5.2. SERS as a cancer biomarker

The progress in SERS-based platforms enables the fabrication of various biosensors for cancer detection. Since the Raman spectra allow to creation of a fingerprint for a particular biomolecule, accurate and sensitive detection of cancer biomarkers can be achieved by SERS. 3D nano-micro fabricated SERS biosensors possess excellent uniformity and unique nanogaps, which enable sensitive and reproducible results for cancer biomarker detection. Li et al. prepared a 3D nanochip with Au@Raman reporter@SiO_2_ for the sensitive detection of vascular endothelial growth factor (VEGF) using SERS 139. The advantage of dimension, shape, and size of AuNPs to act as hot spots in SERS measurement is utilized in this study using malachite green isocyanate (MGITC) as a Raman reporter. The silica shell coating is performed after the conjugation of Au with MGITC so that the Raman reporting molecule is sandwiched between Au and SiO_2_ shell for better Raman response. The silica shell improved the water solubility, and it enhanced the bioconjugation ability of the biosensor. The capture antibody is immobilized on a triangular nanoarray of Au, and the target antibody is conjugated with the Au@MGITC@@SiO_2_ (Where Au = nanosphere and nanostar) using the carbodiimide chemistry. The studies showed that the Au nanostar/Au triangle nanoarray exhibited high sensitivity for the vascular endothelial growth factor with an LOD of 7 ± 5 fg/mL. The present study using the 3D nanochip showcased that the present immunosensor shows better performance than the conventional ELISA method within a short time and a robust detection efficiency in complex matrices. Recently, Bhamidipati and coworkers developed a nano biosensor containing Au nanostar for the detection of cancer biomarker epithelial cell adhesion molecule in two cancer cells MCF-7 and PC-3 using SERS with a detection limit of 10 pM 140. Thiolated EpCAM antibodies and aminothiophenol were attached to the surface of Au nanostar for the fabrication of SERS tags with an average tip-to-tip length of 123.1 ± 16.3 nm, and studies showed that these nano biosensors can be used for multiplex detection of cancer biomarkers with proper suitable SERS aptamers.

**Figure 19:**
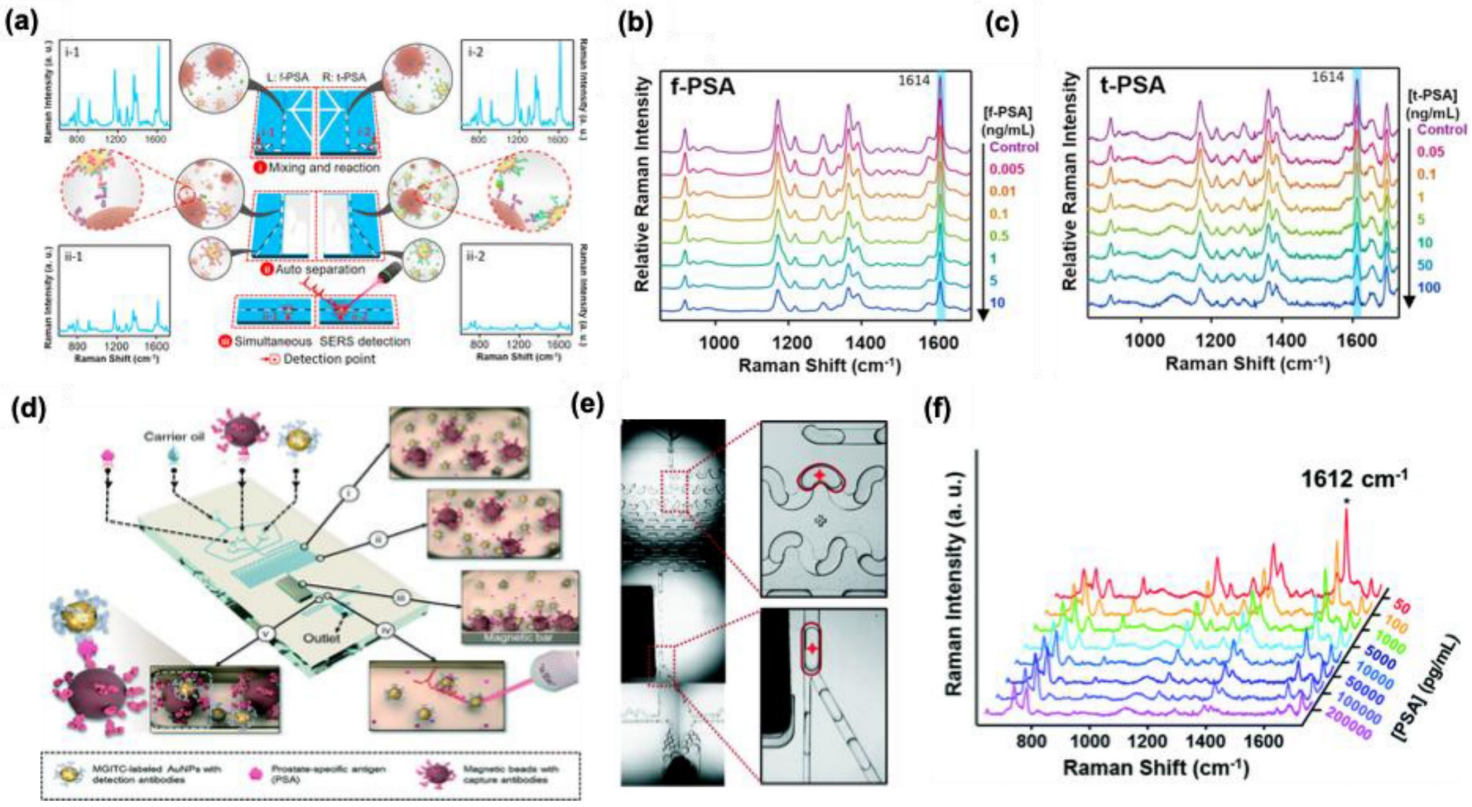
(a) Schematic design and working principle for the parallel microdroplet platform for the detection of f-PSA and t-PSA and the observed SERS spectra at four different channel positions, (b and c) SERS spectra for f-PSA and t-PSA for a concentration range of 0.005 ng mL-1 to 10 ng mL-1 and 0.05 ng mL-1 to 100 ng mL-1, respectively. (d) The schematic representation of the SERS-based microdroplet sensor designed for a wash-free magnetic immunoassay. This sensor features five distinct compartments, each serving specific purposes: (i) the creation of droplets and mixing of reagents, (ii) the assembly of magnetic immunocomplexes, (iii) the separation of immunocomplexes facilitated by a magnetic bar, (iv) the production of larger droplets that incorporate the supernatant for subsequent SERS detection, and (v) the formation of smaller droplets containing the magnetic immunocomplexes. (e) Two SERS detection locations within the microfluidic channel, displayed prior (top) and subsequent (bottom) to the separation of magnetic immunocomplexes. (f) SERS spectra of PSA in the microfluidic channel from a concentration range of 50 pg mL^-1^ to 200 ng mL^-1^. Reproduced with permission from ref 142,143. Copyright 2018 Elsevier and 2016 Royal Society of Chemistry.

Microfluidic-based SERS biosensors are common these days for cancer biomarker detection. In SERS-based assay, the major difficulties are the controlled aggregation of NPs, their optimal size demand, and inhomogeneity while mixing with the analyte renders their performance in the detection of various biomarkers. The incorporation of SERS tags in the microfluidic system can overcome these difficulties and allow a continuous flow and homogenous environment for the assay. Gao et al. developed a microfluidic device that shows enhanced multiplex detection ability for free prostate-specific antigen (f-PSA) and the total PSA (t-PSA) cancer biomarkers **(Figure 19a)** 141,142. AuNPs were tagged with MGITC Raman reporter, and -SH-PEG-COOH were utilized to couple the f-PSA and t-PSA antibodies, which were finally with AuNPs. The results showed that the microfluidic system can detect 0.1 ng mL^-1^ for both f-PSA and t-PSA **(Figure 19b-c)**. This SERS microfluidic channel offers higher accuracy and sensitivity with an instantaneous response in the Raman spectrum than the existing electrochemiluminescence methods for prostate cancer assay. The same group has developed a microfluidic system with a magnetic immune assay for the detection of PSA **(Figure 19d)** 143. This technique allows the wash-free immune assay in the serum with a detection limit of 0.1 ng mL^ -1^ **(Figure 19e)**. The PSA detection antibody was conjugated with AuNPs bearing MGITC whereas the capture probe was conjugated with carboxyl magnetic particles using carbodiimide chemistry. Due to the antigen-antibody interaction, an immunomagnetic complex will be formed, and using a magnetic bar, the uncomplexed derivatives can be removed easily allowing a homogenous mixing and enhanced SERS response in the microfluidic channel **(Figure 19f)**. Zheng et al. prepared a multichannel microfluidic SERS-based system for the simultaneous detection of A153, CA125, and CEA breast cancer biomarkers in human serum using mercaptobenzoic acid-AgNPs as SERS tags 144. The studies showed that the fabricated immunoassay platform showed performance almost like an ELISA kit, with an excellent multiplex efficiency and possessing a limit of detection 0.0001 U mL^-1^ for CA125 in buffer solutions. Cong et al. prepared a microfluidic-droplet platform using AgNPs and 4-mercaptophenylboronic acid as a Raman reporter and capture probe for the sialic acid which is a biomarker for cancer cells. The SERS studies were used for imaging the sialic acid and quantifying them in different cancer cell lines like MCF-7, HepG2, SGC, and BNL.CL2 145.

Multiplex detection of biomarkers can be achieved by the SERS technique and fabrication of cheap and easy platforms are still challenging. Research groups have utilized various fabrication techniques like thin films and paper-based assays for the detection of cancer biomarkers. Wang et al. showed that multiplexed SERS detection can be performed with prostate specific antigen (PSA) and α-fetoprotein (AFP) using a thin film SERS substrate simultaneously 146. Polystyrene nanospheres were coated with Au film to prepare the immune substrate using the electron beam evaporation method. Two different Raman reporting molecules were used whose Raman peaks show different fingerprint features in the spectrum (4-mercaptobenzoic acid and 4-nitrothiophenol acid). After conjugation of these Raman reporting molecules with Ag@SiO_2_, a sandwich immune complex is fabricated with the gold-film hemisphere array. The studies showed that these sandwich nanomaterials can detect 3.38 and 4.87 fg mL^-1^ for PSA and AFP, respectively, suggesting these SERS-based multiplex immunoassays can be used for clinical diagnosis of cancer.

### 5.3. For the detection of proteins

Detection and quantification of proteins are very crucial in biomedical engineering fields since they play a vital role in many biochemical reactions. Compared to other techniques like ELISA and fluorescence, the detection of protein by Raman spectroscopy can give more vital information since the technique is performed by the vibrational and rotational motions of the structural units in proteins. Proteins carry a net positive, negative, or zero charge, depending on the media and environment. Electrostatic interactions between proteins and aggregated metal nanoparticles serve as a platform for SERS-based detection. 2D nanomaterials can provide enhanced SERS intensity compared to the dispersed nanoparticles but the proteins that are in proximity to metallic nanoparticles can give intense SERS signals suggesting the reproducibility issue using such 2D nanomaterials. Whereas 3D metallic nanostructures provide an ordered arrangement of plasmonic hot spots with an appropriate nanogap between the analyte and hot spots and they mostly eliminate the signal backgrounds and the charge properties of the biomolecule.

Kahraman et al. fabricated well-defined plasmonic 3D metallic structures using soft lithography and nanosphere lithography for the label-free detection of proteins 147. The nanovoid 3D metallic structure was prepared by a combination of soft and nano-sphere lithography. The sulfate latex particles of diameter 1600 nm were coated with PDMS elastomer microparticles to create a bowl-shaped nanovoid structure in the PDMS. Following this, a layer of chromium (thickness 5 nm) and Ag (thickness 60 nm) was sputtered in the voids to produce 3D plasmonic structures with high stability and flexibility which allowed the hot spot centers in the voids for SERS applications. Citrate-capped AgNPs and protein complexes were prepared by mixing the two solutions and making the complex by electrostatic interactions. The SERS performance of these nanovoids was tested using six proteins Bovine serum albumin, hemoglobin, thrombin, avidin, cytochrome c, and lysozyme. The studies showed that due to the difference in surface charge of these proteins, their SERS response is also varied and allowed a label-free detection with accuracy. The negatively charged citrate-capped AgNPs showed strong interaction with cytochrome C, which is positively charged, resulting in the aggregation of AgNPs and strong SERS intensity compared to the other proteins. Since hemoglobin is negatively charged there were no SERS signals obtained for the nanovoids. Among different nanovoids prepared, the platform with 1400 nm diameter and 600 nm depth showed better SERS response using 633 and 785 nm lasers. The bowl-shaped nanovoids provide more contact points with the AgNPs allowing stronger aggregation of AgNPs depending on the charge of the protein and allowing detection even with a low concentration of 0.05 mg mL^-1^. Zhou et al fabricated PDMS microfluidic chip for the sensitive detection of Bovine Serum albumin 148. The microfluidic chip contains several pneumatic valves and nano post arrays and channels that can trap AuNPs (200 nm) as SERS substrate. The size of the nanoparticles is much higher than the size of the nanochannels, the nanoparticles were trapped at the openings and formed the SERS active cues which allow the detection of proteins even in picomolar concentration.

The Raman spectroscopy and SERS technique find application in detecting the Spike and nucleocapsid protein in the SARS-CoV-2 virus. The structural changes and the typical fingerprint pattern generated by Raman spectra enable the accurate detection of these proteins. Sanchez et al. prepared a 3D nondendritic concave nanostar platform from Au and Cu which was further deposited on MoS_2_ film to prepare SERS substrate with a highly localized plasmonic field and surface polarization in the infrared region 149. The experimental studies were performed by analyzing the various amino acid peak positions and this was further supported by the theoretical calculations. The experimental validity was confirmed by analyzing the SERRS spectra of InBios-Spike-Protein and the major amino acid components (Tryptophan and Histidine) were taken as a biomarker. The studies showed that the limit of detection for S protein is 8.89 x 10^-9^ M with high accuracy and reproducibility. Even though this study is preliminary using this technique shortly clinical tests can be performed. SERS technique alone or coupled with other platforms can be useful in the field of protein assay. Even though some reports have been coming in this field the sensitivity towards clinical trials is still challenging, so the proper optimization and fabrication conditions for SERS applications are needed.

### 5.4. Detection of Viruses and Bacteria

As evident from the new COVID-19 pandemic, rapid and sensitive detection of viruses and bacteria is crucial for diagnosis and cure. Most of the existing techniques, such as real-time polymerase reaction, enzyme-linked immunosorbent assay, and electrochemiluminescence methods, use gold standards for diagnosis, and fluorescence-based detection methods are opted 150. These methods lack sensitivity and reproducibility and recently SERS based detection methods proved they can replace most of the existing techniques for virus detection in terms of sensitivity, reproducibility, and multiplex detection accuracy. In addition, 3D nano-microstructures, due to their large spatial arrangement of hot spots, show excellent SERS enhancement in various detection and sensing applications compared to 1D/2D materials. These fabricated nano-microstructures can be used for the detection of various viral and bacterial genes. The detection method mainly depends on the ability of Raman spectroscopy to identify the different vibrational modes and to make a fingerprint region for various lipids, carbohydrates, proteins, etc. present in the pathogens. Both label-based and label-free SERS detection methods are useful for pathogen detection.

**Figure 20:**
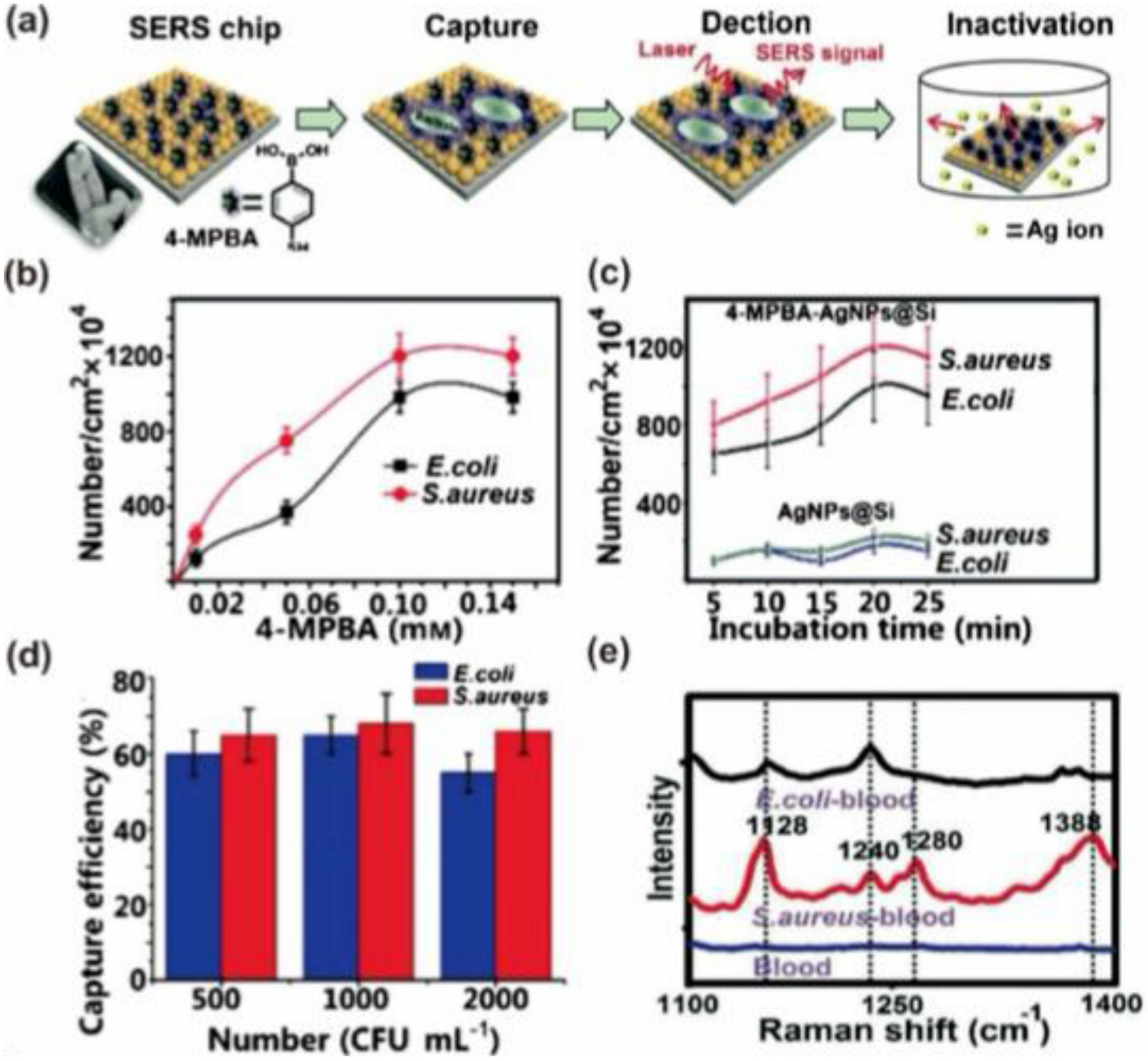
(a) Schematic for the designed SERS chip using AgNPS and 4-MPBA for the bacterial detection, (b,c) the number of bacteria strains at different concentrations and in-cubation times respectively, (d) the bacterial-capture efficiency for the fabricated SERS chip, (e) Raman spectra for the human blood which is spiked with E.Coli and S.aureus, λ_ex_=633 nm. Reproduced with permission from ref 152. Copyright 2015 Wiley VCH.

Mostly the electrostatic interaction between the negatively charged bacterial membrane and positively charged nanocomposite materials is used as a strategy for the analysis of various bacterial strains using SERS. In a previous report by Mevold et al., an Au/graphene-poly(diallyldimethylammonium chloride) nanomaterial with a positive charge on the surface was used as a SERS substrate for the detection of S. aureus 151. The Raman signal intensity at 733 cm^-1^ originated from the glycosidic ring mode from adenine and was examined from the bacterial cell wall and the studies showed that an Au/graphene-PDDA with a molar ratio 4:1 exhibited the highest Raman signal intensity. In another study, Wang et al. showed that 4-mercaptophenylboronic acid can be used to bind with the bacterial cell wall with the peptidoglycan, and the thiol group can be used to anchor with AgNPs for SERS applications 152. This SERS chip showed high specificity for the E. coli and S. aureus from human blood with a low detection limit of 1.0×10^2^ cells mL^-1^ **(Figure 20a-e)**. The fabricated nanocomposite material possesses antibacterial activity along with the simultaneous detection of bacterial strains and allows to discrimination E. coli and S. aureus from blood samples **(Figure 20b-d)**. Since the biochemical composition of these bacteria strains are different, they showed different Raman intensity enhancement for the 1128 (amide III),1240 (δ(CH_2_) amide III), and 1388 cm^-1^ (υ(COO-) and δ(CδH) proteins). Since the content of these signature proteins is higher in S. aureus, the Raman spectrum showed a higher intensity for this bacterial strain than the E. coli **(Figure 20e).** A similar strategy was used for the detection and differentiation of bacteria strains from skimmed milk using Ag dendritic nanostructures which allow a detection limit of 103 CFU mL^-1^ for Salmonella enterica which is a food pathogen 153.

Yeh et al. reported a carbon nanotube and gold nanoparticle-based label-free method for the rapid and sensitive detection of viruses from clinical samples 154. The detection platform is labeled VIRRION which was fabricated by the chemical vapor deposition technique using nitrogen-doped carbon nanotubes and which were stamped using Fe particles to form well-aligned nanotube arrays with better biocompatibility. AuNPs were coated on these nanoarrays to improve their SERS activity and allowed to create herringbones with dimensions 22 ± 5 to 720 ± 64 nm comparable with that of virus dimensions. This technology successfully allowed viral capture (rhinovirus, influenza virus, and parainfluenza viruses) and the detection took only a few minutes, with a 70-fold enrichment in signal detection.

Fabrication of SERS substrates for label-free detection and use for clinical point-of-care applications need precise control in the nanogaps for hot spot generation. Recently, Kim et al. developed a sensor chip containing AuNPs decorated on the surface of ZnO nanorods which are vertically grown on the surface of cellulose paper 155. This three-dimensional platform showed an enhanced enhancement factor of 10^7^ with high reproducibility in the Raman signal for amniotic fluid detection. Wang et al. fabricated a 3D Au nanopillar substrate using thermal evaporation methods for the detection of three mycotoxins (ochratoxin A, fumonisin B, and aflatoxin B1. The controlled fabrication technique allows the synthesis of SERS substrate with controlled nanogap between the nanopillars with high-intensity hot spots for the pg mL^-1^ detection of three mycotoxins 156.

Ko et al. reported a 3D nanopillar Au/Ag core-shell array for the detection of bacterial pathogens. Using the argon plasma etching method, polyethylene terephthalate polymer pillars were developed first, and metals were deposited on the pillars using the thermal evaporation method 157. The 3D nanopillar was further functionalized with polylysine bearing a positive charge for the adsorption of bacterial pathogens via electrostatic interactions. For the fast Raman mapping, antibody-conjugated SERS nanotags AuNPs-malachite green isothiocyanate-anti Salmonella antibody was synthesized by the carbodiimide chemistry. Raman mapping images were collected, and the studies showed that this fabricated substrate can do Raman imaging for different concentrations of bacteria from 0 to 10^6^ CFU mL^-1^ range with 3 orders of magnitude higher than the other techniques. Recently Das et al. fabricated Ag-capped aluminum nanorods for the detection of E. coli bacteria in a wide range of concentrations from 10^8^ CFU ml^-1^ up to 10^2^ CFU ml^-1^ using the glancing angle deposition method. The advantage of this method is using cheap material like aluminium reduces the cost of such synthesis and it can create a porous structure in the Ag surface for SERS applications 158.

### 5.4. Detection of exosomes

Extracellular vehicles (EVs) are small, membrane-bound structures present in various cell types. They facilitate intercellular communication by transporting biomolecules such as proteins, lipids, and nucleic acids to recipient cells. EVs are categorized based on their size, origin, and mode of formation 159-161. The two primary types are exosomes and microvesicles. Exosomes, typically 40-150 nm in diameter, originate from the endosomal pathway, whereas microvesicles, ranging from 100 to 1000 nm, form through outward budding of the plasma membrane. EVs have been implicated in cancer progression, with elevated levels often observed in individuals with advanced-stage cancer compared to healthy individuals 162,163. As a result, EV research is rapidly expanding, with exosomes and microvesicles emerging as potential biomarkers for cancer diagnosis and treatment.

**Figure 21:**
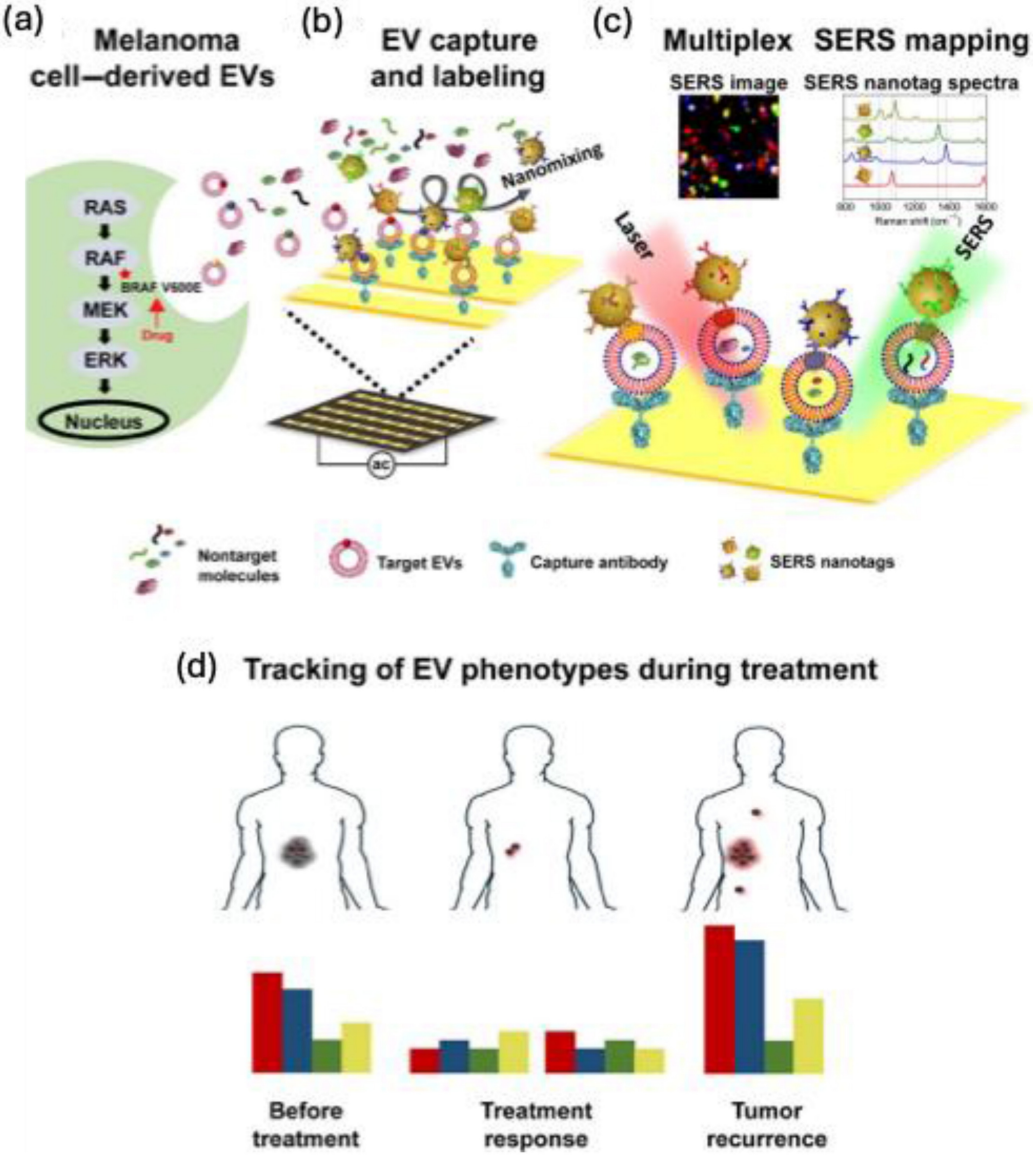
(a) Melanoma cells with a BRAF V600E mutation release EVs into circulation or culture medium. (b) The sample is injected into analyser chip, where nanomixing enhances EV-antibody interactions while removing non-target molecules. (c) EV phenotypes are characterized via SERS mapping, with false-color spectral images based on SERS nanotag peak intensities. (d) Unique EV phenotypes, defined by biomarker expression, enable tracking of phenotypic evolution during BRAF inhibitor treatment, offering insights into treatment response and early drug resistance. Reproduced with permission from 164. Copyright 2020, AAAS.

SERS is a powerful technique for detecting and analyzing EVs with high sensitivity and specificity. Various SERS-based platforms have been developed for exosome detection. Wang et al. demonstrated the potential of a multiplex EV phenotype analyzer chip, which integrates a nanomixing-enhanced microchip and a multiplex SERS nanotag system, enabling direct EV phenotyping without enrichment **(Figure 21a-b)** 164. Different types of SERS nanotags were designed to specifically target individual biomarkers: 4-mercaptobenzoic acid (MBA) for MCSP, 2,3,5,6-tetrafluoro-MBA (TFMBA) for MCAM, 5,5′-dithiobis (2-nitrobenzoic acid) (DTNB) for ErbB3, and 4-mercaptopyridine (MPY) for LNGFR. The detection process involved SERS mapping, where a false-color spectral image was generated based on the distinct Raman reporter peaks—1075 cm⁻¹ for MBA, 1375 cm⁻¹ for TFMBA, 1335 cm⁻¹ for DTNB, and 1000 cm⁻¹ for MPY. The intensity of signals within the mapped region corresponded directly to the quantity of EVs and the expression levels of their associated biomarkers **(Figure 21c)**. As shown in **Figure 21d**, the system was utilized to characterize EV phenotypes at different stages of treatment—before, during, and after. False-color SERS spectral images were analyzed to determine EV phenotypes by assessing the relative intensities of Raman reporter peaks. It was proposed that shifts in overall EV phenotypic profiles might correspond to variations in cancer cell populations throughout treatment, offering potential for monitoring patient treatment responses. Another study introduced a method to simultaneously analyze multiple protein biomarkers on pancreatic cancer-derived EVs using SERS tags, eliminating the need for complex isolation 165. A portable Raman-based SERS assay enabled rapid phenotypic profiling of small EVs by detecting surface receptors (Glypican-1, EpCAM, and CD44V6) with a limit of detection of 2.3 × 10⁶ EV/mL. This approach facilitated treatment monitoring and early drug resistance detection by analyzing EV samples before, during, and after BRAF inhibitor therapy.

Kiwizera et al. developed a cost-effective, portable Raman-based exosome assay using an antibody-based capture array on Au nanorod substrates for HER2 and EpCAM biomarker detection in breast cancer patient plasma, though it required prior exosome isolation 166. Recently, combining liquid biopsy, AI, and SERS enabled the simultaneous diagnosis of six early-stage cancers via direct plasma exosome analysis 167. Similarly, Parlatan et al. utilized machine learning-assisted SERS to trace EVs back to their cellular origins, distinguishing cancer-derived exosomes from healthy ones. Dong et al. designed an Au-coated TiO₂ macroporous inverse opal structure inspired by beehives, enhancing SERS performance via the “slow light effect 168.” This label-free method detected cancer exosomes from patient plasma, identifying a correlation between the P-O bond in phosphoproteins and the SERS peak at 1087 cm⁻¹, which was at least twice as intense in cancer patients compared to healthy individuals. This underscores the versatility and potential of SERS for cancer diagnostics.

### 5.5. Other applications in Bioanalysis

Fabrication of highly sensitive and reproducible platforms for bioanalytical assays and sensing studies is still challenging. Advances and recent studies have shown that SERS-based assays and techniques can overcome these difficulties with high reproducibility and sensitivity. Three-dimensional (3D) nano-microfabricated biosensors have been used for various biomedical and small molecule sensing applications. In this section, we will cite some relevant examples of such 3D biosensors used for sensing various biomolecules and for the catalysis process.

In the beginning part of the review, it was highlighted that in the case of low-dimensional geometries, the hot spots needed for SERS applications are localized at a single point or randomly, resulting in a low enhancement in the Raman signal intensity. However, in 3D SERS platforms, due to the large volume in the 3D space, the electric field enhancement is not only limited to a particular point, or due to the ordered nanostructure they possess finite nanogaps for SERS applications. Among the different methods for the fabrication of SERS substrates the most employed one is the fabrication of 3D morphologies using plasmon-free inorganic or organic materials using the various micro-nano fabrication techniques and then modifying its surface features with plasmonic nanoparticles for developing SERS platforms.

**Figure 22:**
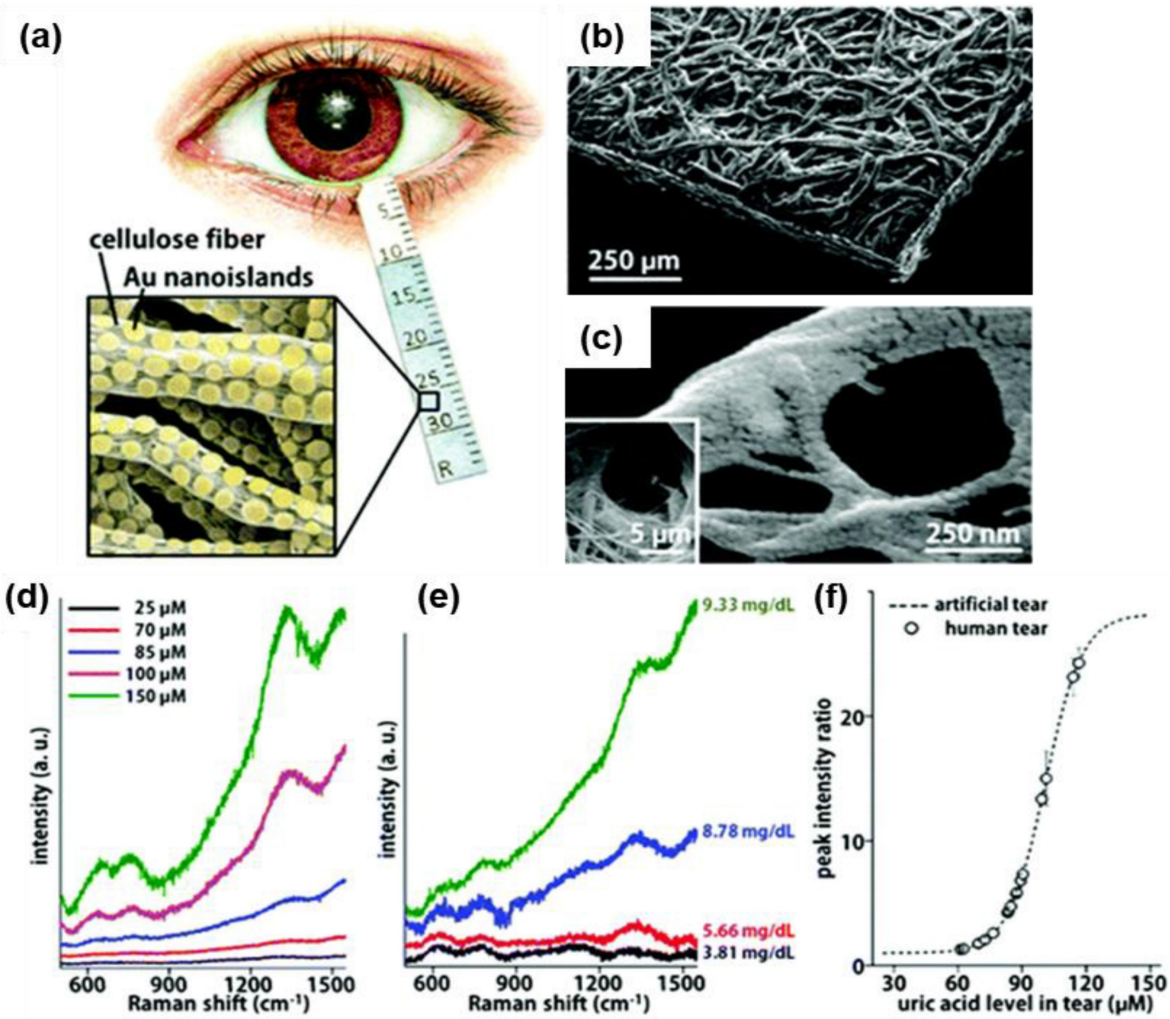
(a) Represents the SERS-based tear screening using Schirmer plasmonic strip with Au nanoislands, (b,c) The top and cross-section SEM image respectively for the strip, (d,e) SERS spectra of uric acid with varying concentrations for artificial and human tears, (f) The comparison of uric acid level in tears of human tears and artificial tear solutions. Reproduced with permission from ref 170. Copyright 2017 American Chemical Society.

3D nano-microstructures can be prepared using simple techniques such as template-wetting. Filter papers containing cellulose fiber can be used as a template. Zheng et al. demonstrated that filter paper can be converted into a 3D SERS by dipping the paper in a toluene solution of AuNP and after drying a homogenous SERS platform can be fabricated with 10-15% intensity variation for monitoring the catalysis of reduction of aromatic nitro compounds to corresponding amines using sodium borohydride 169. This platform allowed for monitoring of the characteristic Raman peaks for the products and reactants with high reproducibility and accuracy. In another work, Park et al. fabricated a Schirmer test strip paper for the detection of uric acid in human tears using SERS **(Figure 22a)**. The test paper is fabricated using cellulose paper and coated with a thin film of Au to form highly ordered nanoislands in between the cellulose fibers as confirmed by the SEM studies **(Figure 22b-c)**. The advantage of this test strip is that it possesses highly hygroscopic micro/nanopores and as a result, it can be used in the lower eyelids for the sample analysis. The amount of uric acid in the artificial tear drops was analyzed using Raman spectroscopy for the characteristic peaks at 660, 756, and 1342 cm^-1^ for uric acid within the range of 25 to 150 μM **(Figure 22d-f)**. This SERS-based detection allows the detection and quantification of uric acid in human teardrops with an amount of 68 ± 46 μM with accuracy without any labeling agent. The same platform can be used as a biomarker for the identification of gouty arthritis, in such patients the uric acid level will be greater than 95 μM 170.

3D nano-micro fabricated SERS platforms can also be generated by controlling the assembly of various templating agents to create the 3D hot spots from the nanoparticles. Various polymeric materials can be used for this purpose to create 3D gels or nanofibers to entrap the metal nanoparticles into the polymer assembly and create 3D hot spots for SERS applications. Abalde-Cela et al. showed that hydrogels from gellan gum possess a sponge-like morphology and incorporation of Au nanostar or Au/Ag nanorod to possess excellent SERS response towards lactate and thiocyanate, which are cancer cell metabolites found in the extracellular matrix 171. In another study, Malvadkar et al. demonstrated that poly(chloro-p-xylylene) PPX nanofiber embedded with a thin film of gold can be used as an excellent SERS substrate for the detection of respiratory syncytial virus genes. Incorporation of Au into the polymer nanofibers offers a uniform nanogap and electric field enhancement (EF = ∼10^6^) and offers a detection limit of ∼10^-10^ M of the virus gene 172. Using different Raman reporting molecules Hex and Cy5 they have demonstrated that this nanofiber can be used for multiple DNA target detections.

Detection of small biomolecules using SERS is quite interesting and promising. Since the imbalance of such biomolecules can correlate with many diseases, the early detection and diagnosis of such biomolecules are quite relevant for clinical applications. SERS-based detection offers mostly label-free detection and provides high detection sensitivity and specificity compared to other existing techniques. Vijayakumar et al. fabricated a 3D nanohybrid platform using nickel as a SERS hot spot generator for the detection of glutathione which is a cancer biomarker 173. The study highlights the importance of a non-noble metal-based substrate that is free from chemical oxidation and possesses high biocompatibility and MRI imaging efficiency. The nanonetwork possesses electric field enhancement with an order of 10^9^ which is difficult to attain with noble metal nanoparticle systems and offers a detection limit of 1 pM. The observed high enhancement factor here is due to the combined charge transfer between the Ni nanonetwork and glutathione, and the 3D nanonetwork offers an intense hot spot due to the self-assembly. In another study, Nam et al. reported a 3D nanolaminate plasmonic crystal platform coupled with Au for the enhanced detection of dopamine which is a neurotransmitter 174. The SERS analysis is based on the selective binding of two Raman-reporting molecules with dopamine. The 3D nanolaminate platform provides a uniform nanogap for SERS analysis. Among the two Raman reporting molecules coated on the Au surface, 3,3′-dithiodipropionic acid forms amide bonds with the amine group of dopamine using N-hydroxy succinimide, whereas the diol group forms a chelate ring with the other Raman reporting molecule 3-mercaptophenyl boronic acid. The platform offers an on-off response in SERS signal depending on the presence and absence of dopamine and allows a detection limit even lower than 1 pM.

Multiple target detection is an advantage of SERS-based platforms. Li et al. fabricated a 3D cauliflower-type SERS material from Au using the sputtering technique on a PDMS surface with anodic aluminum oxide 175. The 3D SERS platform with a cauli-flower-like structure possesses highly ordered contact areas with many grooves and bulges in the surface which can act as hot spots for the SERS application. The SERS substrate was used for the analysis of three mycotoxins in a maze-like aflatoxin B1, zearalenone, and deoxynivalenol with a detection limit of 1.8, 47.7, and 24.8 ng mL^-1^ respectively. The 3D cauliflower-type SERS platform demonstrated that the sputtering technique can be used for fabricating SERS surfaces for label-free detection with high enhancement factors (EF = 2.2 × 10^6^). As a part of the discussion, the reported works demonstrated that the SERS technique can be used for various biomedical applications. The high sensitivity and multiplexing capability of this technique offer promising applications for the detection and diagnosis of various diseases.

## 6. SERS-based bioimaging applications

Historically, fluorescence-based bioimaging has outpaced techniques utilizing Raman spectroscopy. Recently, however, advancements in Raman instrumentation and an improved understanding of SERS principles have led to the development of SERS-active nanoprobes that perform similarly to fluorescence labels. ​Notably, SERS-based nanoprobes often provide advantages over their fluorescent counterparts in bioimaging applications, including heightened sensitivity, specificity, the ability to multiplex, enhanced biocompatibility, and greater photostability.​ Consequently, SERS tags are increasingly being adopted as rival imaging agents for bioimaging in both *in vitro* and *in vivo* settings. By utilizing SERS tags that are attached to specific biological targets, researchers can create highly sensitive assays to identify disease-related molecules *in vitro* and *in vivo* 176-178. This capability enhances the accuracy of tumor diagnostics and allows for the delineation of tumor margins during surgical procedures, improving clinical outcomes. Furthermore, recent advancements have integrated artificial intelligence and deep learning algorithms with SERS techniques to analyze complex biomolecular signatures. This approach not only enhances detection accuracy but also allows for the differentiation of various biomolecular profiles, which is crucial for personalized medicine.

### 6.1. *In vitro* bioimaging applications

SERS nanotags can detect various biomarkers and imaging applications for cancer models, and tissue samples. *In vitro* bioimaging utilizing SERS offers several compelling benefits. It provides high spatial resolution and specificity, enabling the visualization of complex biological processes in real time. Furthermore, SERS can be applied to a wide range of biomolecules, including proteins, nucleic acids, and metabolites, making it a versatile tool for researchers 176,179,180.

**Figure 23:**
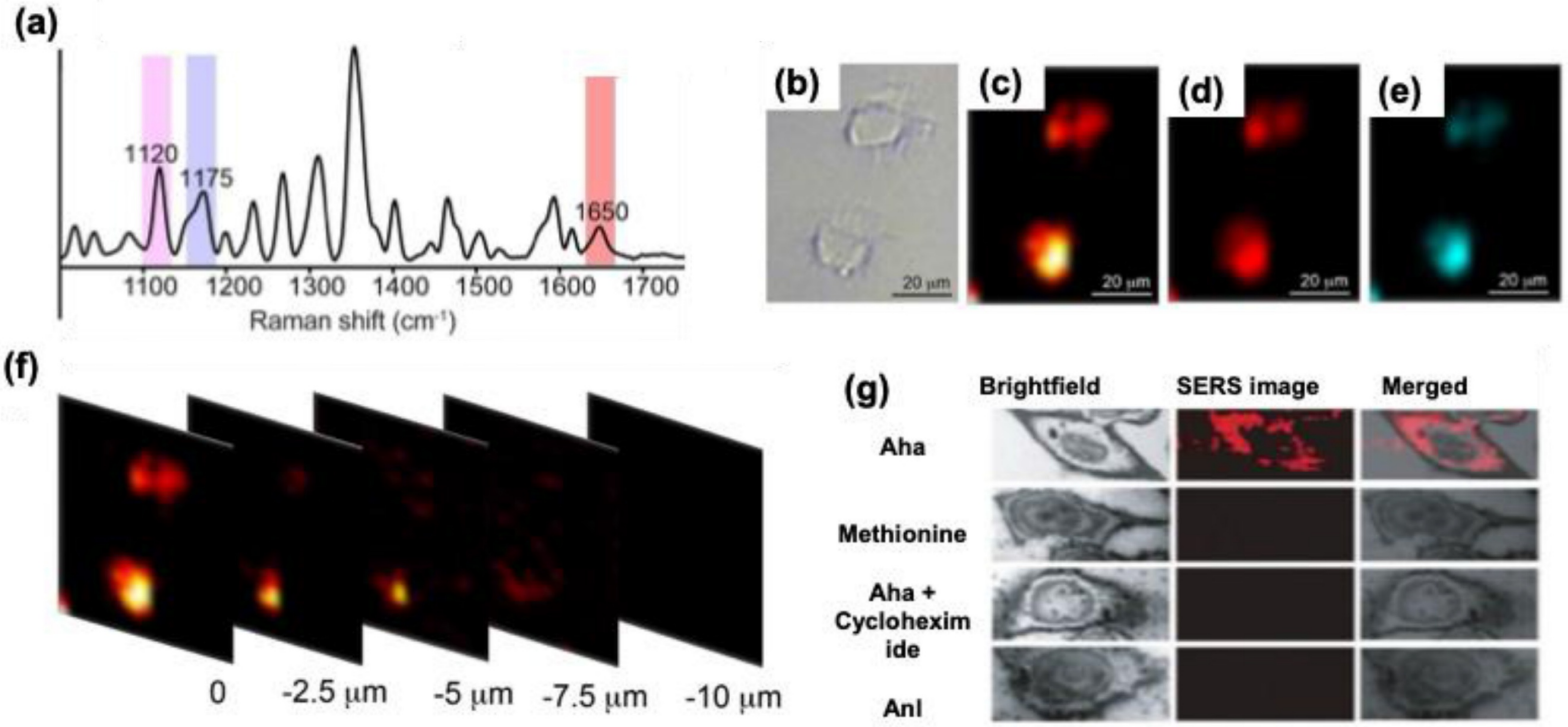
(a) SERS spectra for the fabricated SERS nanotag with Cy5, malachite green isothiocyanate, and Rhodamine 6G. (b) the bright field cell image for the MDMBA cell line with SERS tags. (c-e) The SERS intensity map image illustrates the presence and spatial distribution of biomarkers TGFβRII, CD44, and EGFR on the cell surface, which are attached to antibody-conjugated SERS nanotags: Cy5, malachite green isothiocyanate, and Rhodamine 6G, respectively.​ SERS mapping was performed using the peaks at 1120 cm^-1^ for Cy5, 1175 cm^-1^ for malachite green isothiocyanate, and 1650 cm^-1^ for Rhodamine 6G. (f) The SERS intensity mapping images of the z-series scan for the Cy5 nanotag, which is associated with the TGFβRII biomarker, were obtained at varying depths with a spacing of 2.5 μm. (g) SERS imaging using self-assembled AuNPs, cell-surface proteins were tagged metabolically with Aha, while cells treated with methionine, cycloheximide, and Anl served as negative controls in the second to fourth rows. Reproduced with permission from ref 179,182. Copyright 2014 Nature and Wiley publishers.

In one of the studies, Dinish et al. showed that multiplex detection of three intrinsic cancer biomarkers-EGFR, CD44, and TGFβRII in a breast cancer model can be achieved 179. Three different SERS tags such as malachite green isothiocyanate, Rhodamine 6G, and Cy5 were added into the center of the tumor on a subcutaneous MDA-MB-231 breast cancer xenograft mouse model. Raman analysis showed the characteristic peaks for the three SERS tags from the cell surface **(Figure 23a)**, and which was further validated by the bright field images from the cell surface for the three biomarkers **(Figure 23b)**. The intrinsic cancer biomarkers distribution on the cell membrane is determined by mapping the corresponding Raman peaks of the SERS nanotags, which verifies the specific binding and interaction of the conjugated nanotags with three biomarkers depicted in **Figure 23c-e**. To confirm the attachment of the nanotags to the cell surface, Raman mapping was performed for all three nanotags at intervals of 2.5 μm. An example of the image stack for the Cy5 nanotag linked to TGFβRII is presented in **Figure 23f**. ​Consequently, the ability to simultaneously detect multiple biomarkers holds significant promise for enhancing both the sensitivity and diagnostic precision of various cancer types.

To improve the multiplexing capability of SERS, various nanostructures embedded with SERS tags were used. Yuan et al. showed that gold nanostar conjugated with various SERS tags can be used for the multiplex detection of various biomarkers and can be used for imaging applications in both *in vitro* solutions and *ex vivo* tissue samples under NIR excitation 181. Xiao et al. conducted an experiment where they cultured cells on silicon wafers or glass slides that had been treated with Ag NP films 182. The cells were then tagged with Aha and subjected to precursors equipped with bioorthogonal reactive (Ra) molecules. Utilizing self-assembled arrays of gold nanoparticles (AuNPs), they successfully highlighted the newly synthesized proteins, glycans, and lipids present on the surfaces of the cells. ​This approach enabled SERS bioimaging of diverse membrane molecules by incorporating various Ra molecules, including azides, alkynes, and C-D bonds **(Figure 23g)**.​ Moreover, the use of reporters with distinct Raman frequencies for multi-color SERS bioimaging holds significant potential for the simultaneous visualization of multiple biomolecules, thereby providing an additional means of live-cell microscopy alongside traditional fluorescence and label-free imaging techniques.

Recently, Liu et al. introduced an innovative folate-targeted SERS nanotag aimed at the selective bioimaging and diagnosis of cancer cells that overexpress folate receptors 183. They achieved this by incorporating a monolayer of Raman-active azide derivatives onto the surface of AuNPs, which facilitated a higher density of labeling molecules. This layer was then effectively conjugated with folate cyclooctyne derivatives through a copper-free click chemistry reaction. The resulting SERS nanotags demonstrate the ability to specifically attach to folate receptor-positive cancer cells, allowing for dark-field imaging and the acquisition of SERS bioimages that reveal the distribution of these nanotags across various cancer cells exhibiting different folate receptor levels. ​This approach showcases significant promise as a robust bioimaging tool for targeted tumor therapies.

### 6.2. *In vivo* bioimaging applications

The application of SERS *in vivo* imaging holds great promise for various fields, including cancer diagnostics, drug delivery monitoring, and the study of metabolic pathways. The non-invasive nature of the technique allows for the continuous observation of physiological changes over time, offering invaluable insights into disease progression and therapeutic efficacy. Through the development of biocompatible SERS-active nanoparticles, researchers are now able to target specific tissues or cells, enhancing the specificity and sensitivity of their imaging methods 176,180. As the technology continues to evolve, the integration of SERS with advanced imaging modalities, such as microscopy and endoscopy, is likely to expand its applicability in clinical settings, ensuring more accurate diagnoses and personalized treatment strategies. One of the first reports by Nie and colleagues, demonstrated *in vivo* SERS imaging in a mouse xenograft tumor model 184.​ In their approach, they utilized spherical gold nanoparticles that were functionalized with a mixed layer consisting of a resonant SERS label, malachite green, alongside thiolated polyethylene glycol derivatives. These nanoparticles were further conjugated with antibodies that specifically target EGFR-positive tumors. After intravenous injection into the bloodstream, the nanoparticles exhibited a preferential accumulation in the tumor region within 4 to 6 hours, remaining localized for more than 24 to 48 hours. This prolonged retention facilitated effective spectroscopic detection of the tumor via SERS, evidenced by the acquisition of the distinct vibrational signature of malachite green. Additionally, there were observed lower but noteworthy nonspecific uptakes of the particles by the liver and spleen.

**Figure 24:**
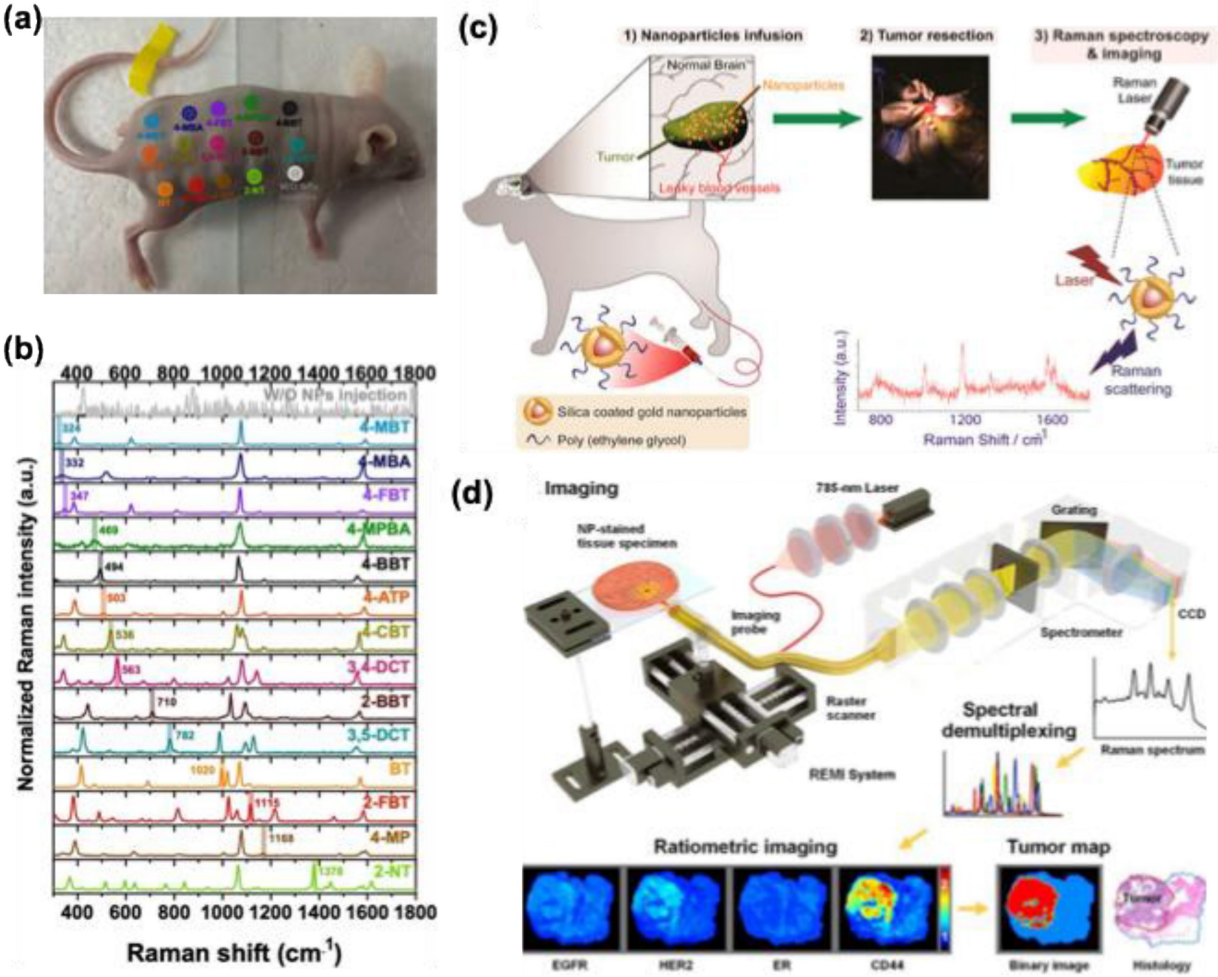
(a) Digital photograph showing the 14 different SERS tags in the tumor model. (b) Comparison of the normalized Raman spectra of SiO_2_@Au@Au-RLC injected into a nude mouse with spectra obtained from a non-injected site. Measurements were performed under 785 nm photoexcitation with a laser power of 2.1 mW and an acquisition time of 10 seconds. ​Each spectrum displayed distinct features, enabling the identification of unique Raman bands associated with the labels. (c) schematic representation for the administration and brain tumor imaging for canine tumor model using SERS. (d) A schematic representation of the entire Raman-encoded molecular imaging (REMI) process, encompassing the stages of staining, imaging, and spectral demultiplexing. Reproduced with permission from Ref 186,190,191. Copyright from 2022 Springer publishing group, 2019 American Chemical Society, 2017 American Association for cancer research.

Wang et al. showed that using SERS imaging the different stages of embryo development can be monitored in zebrafish 185. Injection of AuNPs with SERS nanotags allowed to map the intensity of carbon-carbon bond at 1078 cm^-1^, in the body musculature of the zebrafish embryo. The technique was further extended for the multiplex detection of SERS tags and used to distinguish the developed cells and tissue types *in vivo*. Bock et al. fabricated self-assembled gold nanostructures with controllable nanogaps (4.16 to 0.98 nm) for the detection of tumors in nude mice 186. 14 different nanotags were prepared using SiO_2_ coating on the AuNPs and injected into subcutaneous mice **(Figure 24a-b)**. The present study highlights the importance of NIR labels for tumor progression and diagnosis applications. Raman analysis showed 14 distinct Raman spectra with color intensity variations depending on the Raman cross-section of each tag.

Noninvasive detection of tumors is quite interesting and challenging. Nicolson et al showed that using surface-enhanced resonance Raman spectroscopy (SERRS) accurate and rapid detection of tumors is possible without any surgical procedures. In the studies, they showed that RGD conjugated gold nanostar tagged with Raman reporter molecule can be used for the imaging of glioblastoma multiforme in the skull of mice without damaging the skull 187. In another study, they showed Spatially Offset Raman spectroscopy" (SORS) to detect secondary, deeper-seated lesions through the intact skull 188. Similarly, Li et al showed that the use of Au-Ag core-shell and nanodumbell-type structures with SERS tags for noninvasive tumor imaging 189. The study showed that with varying pore size in the nanostructures exceptional near-infrared II SERS imaging can be performed.

From a clinical perspective, SERS-based multiplex imaging has advanced significantly as a diagnostic tool for multimodal imaging, providing valuable support for tumor resections during surgical procedures.​ Recent studies have demonstrated its efficacy in identifying small tumor lesions and facilitating surgical removal. For instance, Gambhir et al. utilized a canine model of spontaneous brain tumors, which closely resembles human conditions, employing an Au@SiO_2_ SERS nanoprobe to achieve sensitive and high-resolution detection of these tumors **(Figure 24c)** 190. The study revealed that the heterogeneous Au@SiO2 SERS nanoparticles were present at various levels within oligodendrogliomas and meningiomas; however, no SERS signals were detected in areas of tumor necrosis or normal brain tissue, underscoring the enhanced permeability and retention effect. A notable aspect of this research was its focus on a canine tumor model, which enabled a thorough evaluation of nanoparticle delivery during surgical removal and associated clinical complications.

A recent clinical investigation employed ratiometric imaging techniques, specifically using Raman-encoded molecular imaging (REMI), to mitigate uncertainties arising from non-specific contrast agents 191. In this study, Liu et al. analyzed 57 fresh tumor tissue samples obtained from 29 patients to measure the elevated expression of four cancer biomarkers: HER2, membrane estrogen receptor (mER), EGFR, and CD44 **(Figure 24d)**. This quantification was achieved by concurrently applying targeted surface-enhanced Raman scattering nanoparticles (SERS NPs) in the localized area. ​The findings indicated that REMI could effectively identify positive surgical margins within a brief timeframe of less than 15 minutes, yielding a specificity of 92.1% and a sensitivity of 89.3%.​ Consequently, this innovative method holds promise for assisting in the precise excision of tumors.

## 7. Artificial intelligence (AI) methods for SERS

Even though SERS provide valuable information and data sets for analysis, many times it's difficult to extract the exact information about the vibrational spectral features of complex analyte mixtures. Recently machine learning and artificial intelligence (AI) techniques help to resolve these issues and enable to extraction of the exact information from complex spectra and analysis. SERS technique overcomes many of the limitations in simple Raman spectra. However, analysis of single-molecule detection from a mixture is still challenging due to the large variation and difference in vibrational features of the molecule which depend on the orientation of molecules towards the SERS materials. However, machine learning and AI techniques allow a plethora of biosensors based on the SERS technique for complex spectrum analysis 2,192,193.

### 7.1. Application of chemometric tool for Raman spectra analysis

Chemometrics employs mathematical and statistical techniques to interpret spectroscopic data. This field encompasses linear data-analysis methods such as principal component analysis (PCA), partial least squares regression (PLSR), cluster analysis, and linear discriminant analysis (LDA), which are fundamental to chemometric studies 194. These techniques are extensively utilized for quantitative analysis and pattern recognition within sample datasets, facilitating both quantitative (e.g., analyte concentration determination) and qualitative (e.g., species differentiation) predictions from spectroscopic data 195,196.

**Figure 25:**
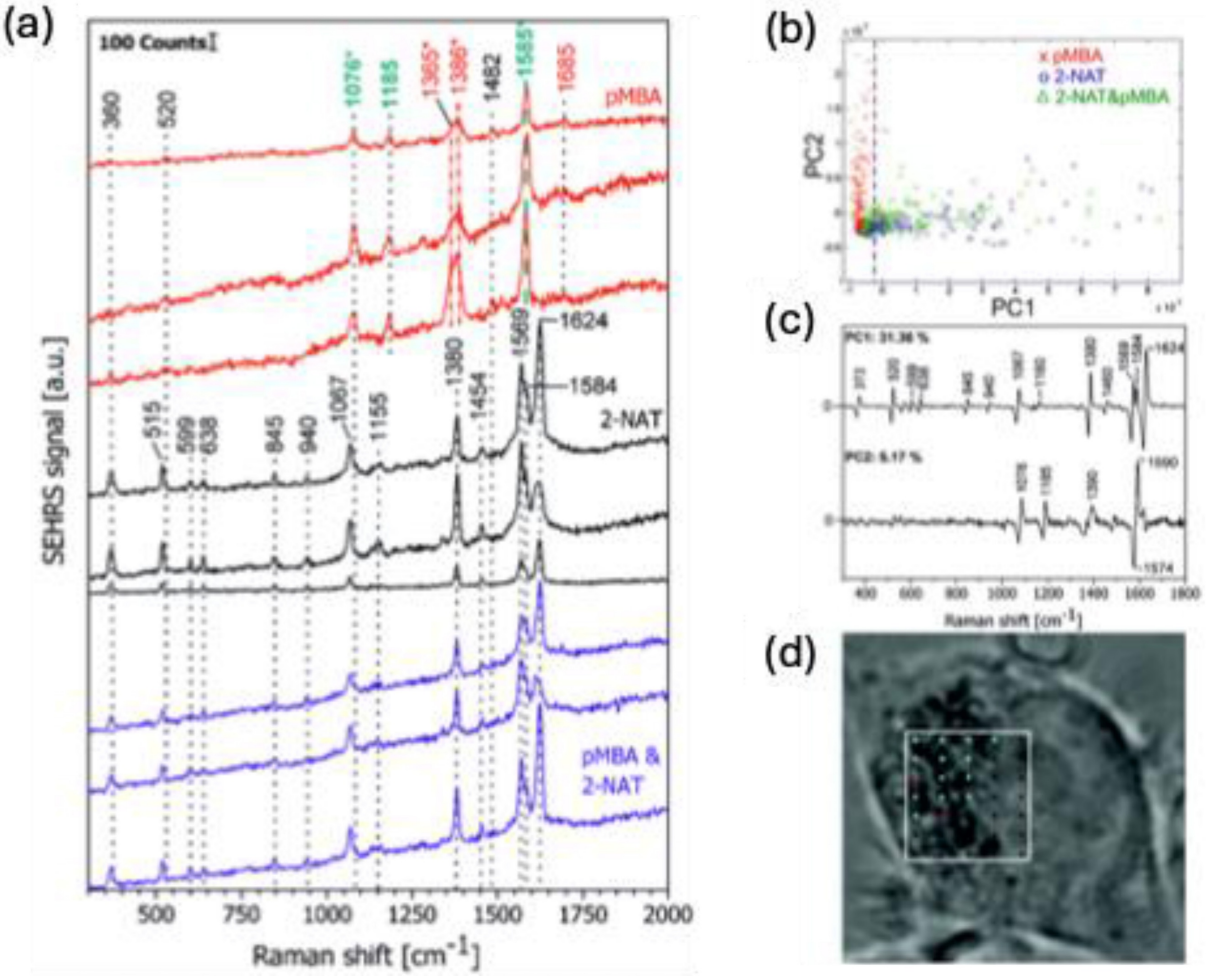
(a) Representative Raman spectra obtained from J774 macrophages for different SEHRS labels: 2-NAT (black lines), pMBA (red lines), and a combination of both labels (blue lines). These spectra were recorded using an excitation wavelength of 1064 nm, with a photon flux density of 3×10^28^ photons/cm²/s, and an acquisition time of 30 seconds. (b) Scores plot of the first two principal components (PCs). The dashed line in (b) marks the threshold for spectral differentiation with red indicating pMBA-like and blue representing 2-NAT-like spectra. (c) Corresponding loadings and explained variance. (d) False-color SEHRS maps reconstructed based on the first PC score, highlighting spectral differences in a sample with both labels. Reproduced with permission from 198 Copyright 2017, RSC publishers.

PCA is among the most widely applied unsupervised chemometric methods for pattern recognition across various scientific disciplines. It serves as a dimensionality reduction tool for SERS data while preserving critical spectral information 194,197. In SERS analyses, data matrices may consist of hundreds or thousands of Raman shifts; PCA condenses these into smaller sets of variables, termed principal components, which capture essential vibrational information. These principal components, constructed as linear combinations of original variables, reflect the highest variance in the data. Visualization of principal component scores and loadings enables the identification of dominant spectral variations and underlying patterns. Heiner et al. utilized PCA to accurately map surface-enhanced hyper Raman scattering (SERHS) probes within cells, further refining their analysis through hierarchical cluster analysis 198. Their study achieved subcellular visualization of macrophage cells using hyper Raman labels conjugated with AgNPs and molecules such as para-mercaptobenzoic acid (p-MBA) and 2-naphthalenethiol (2-NAT), activated at off-resonant wavelengths **(Figure 25a)**. PCA was leveraged for dimensionality reduction and key variable identification, while hierarchical cluster analysis played a crucial role in categorizing similar data points and revealing spectral patterns, allowing precise probe localization. As shown in **Figure 25b**, each point represents a single SEHRS spectrum from various data sets. Red symbols indicate spectra from pMBA SEHRS labels, blue from cells with 2-NAT labels, and green from cells incubated with both. PCA scores **(Figure 25b)** show clear separation of pMBA (red) from 2-NAT (blue) and dual-labeled cells (green) along PC1. **Figure 25c** show loadings, which highlight spectral bands responsible for variance. PC1's loading (upper trace) includes signals at 845, 1160, 1569, and 1624 cm⁻¹, linked to 2-NAT, while PC2 (lower trace) captures variance in pMBA spectra, with prominent peaks at 1585, 1076, and 1158 cm⁻¹. The variance in pMBA spectra aligns with SEHRS sensitivity to pH changes, reflecting data from endosomes at different pH levels. Based on the threshold score, mapping points from dual-labeled cells were assigned along PC1 as either 'pMBA-like' or '2-NAT-like' spectra. False-color SEHRS maps were generated, as shown in **Figure 25d**. The sparse 'pMBA-like' red pixels in the hyperspectral map align with the predominance of 2-NAT-like spectra, consistent with the univariate analysis **(Figure 25d)**.

In another study, Gahlaut et al. employed PCA to differentiate between dengue-positive, dengue-negative, and healthy individuals based on SERS spectra, successfully extracting essential spectral features that distinguished these groups 199. This approach enabled early dengue diagnosis within five days of symptom onset—offering a rapid and sensitive diagnostic method. Similarly, Witkowska et al. applied PCA for bacterial identification, achieving 89% accuracy in differentiating P. gingivalis from A. actinomycetemcomitans in human saliva samples 200,201.

PLSR, a multivariate regression method, establishes relationships between analyte characteristics (such as concentration) and SERS spectral features. By decomposing spectral data into latent variables, PLSR facilitates the development of regression models that correlate spectral patterns with analyte concentrations, ensuring reliable quantification even in the presence of interfering substances in calibration samples 202,203. Fornasaro et al. employed PLSR to quantify the anticancer drug imatinib in human plasma using SERS, constructing a model with three latent variables capable of detecting imatinib in the range of 123-5000 ng/mL with a standard prediction error of 510 ng/mL 202. The model's clinical potential was validated with real patient plasma samples, demonstrating its feasibility for therapeutic drug monitoring in point-of-care settings. Heiner et al. applied PLSR for SERS-based quantification of methicillin-resistant Staphylococcus aureus (MRSA) within bacterial mixtures 204. Their model accurately determined MRSA concentrations ranging from 5% to 100% within mixed samples, underscoring its clinical relevance. Hou et al. utilized PLSR for quantifying food preservatives such as potassium sorbate and sodium benzoate using SERS spectra, successfully establishing regression models correlating spectral features with analyte concentrations 205.

Cluster analysis is a statistical approach used to categorize SERS spectra, particularly when spectral differences are subtle. By identifying inherent clusters within spectral datasets, this method enhances classification accuracy. Various clustering algorithms, including hierarchical clustering, k-means clustering, and density-based clustering, have been employed to improve group differentiation 206. Hierarchical clustering is widely applied in SERS-based studies 207-209. Ma et al. proposed an antibody-independent technique for protein differentiation using SERS and hierarchical clustering, utilizing perylene tetracarboxylic acid as a linker to interact with proteins based on symmetry disruptions and frequency shifts 207. Their method successfully distinguished 10 proteins with varying molecular weights and structural properties based on distinct spectral patterns. Similarly, Zhou et al. utilized hierarchical clustering to differentiate three E. coli strains and one S. epidermidis strain by coating bacterial cell walls with AgNPs, enhancing their SERS signals 206. This technique allowed precise bacterial strain classification through spectral pattern analysis.

Discriminant analysis (DA), including LDA, is a supervised classification technique that distinguishes SERS spectra based on defining spectral features. Unlike cluster analysis, which is unsupervised and groups similar data points without predefined categories, LDA maximizes class separability using linear combinations of spectral variables 192,210. LDA is most effective when the number of variables does not exceed the sample count; otherwise, preliminary dimensionality reduction techniques such as PCA are required 211. Lee et al. demonstrated a SERS-based biochip for renal function assessment in ischemic rats using urine and blood samples. PCA and PLS-DA were employed to extract Raman spectral features, with PCA alone failing to adequately differentiate samples, whereas the combination with PLS-DA achieved 99.9% and 99.3% accuracy in urine and blood samples, respectively 212. Das et al. applied PCA and LDA to analyze SERS spectra for diabetes detection, using receiver operating characteristic (ROC) curves to evaluate model sensitivity and specificity 211. This method enabled highly accurate discrimination of diabetic individuals based on SERS spectra from blood plasma samples. Huefner et al. developed a PCA-LDA approach for characterizing intracellular endocytic compartments, generating color-coded maps that illustrated endosome and lysosome distribution in neuroblastoma cells and their interactions with nanoparticles 213. This technique facilitated the identification of unknown intracellular structures and biochemical components using a reporter-free SERS approach.

### 7.2. Machine learning and AI technique for SERS

In AI techniques using the raw data from the experiments, information will be extracted, and using decision-making the real-world problems are solved with the help of linear discriminant analysis (LDA), principal component analysis (PCA), or artificial neural network (ANN) 214. These techniques allow for simulating the spectra which appear to be noise in the normal SERS due to the complexity but possess valid information that can be enhanced for analysis. In this section, we will highlight a few examples in which machine learning and AI techniques are involved in SERS for developing biosensors for complex analyte detection.

**Figure 26:**
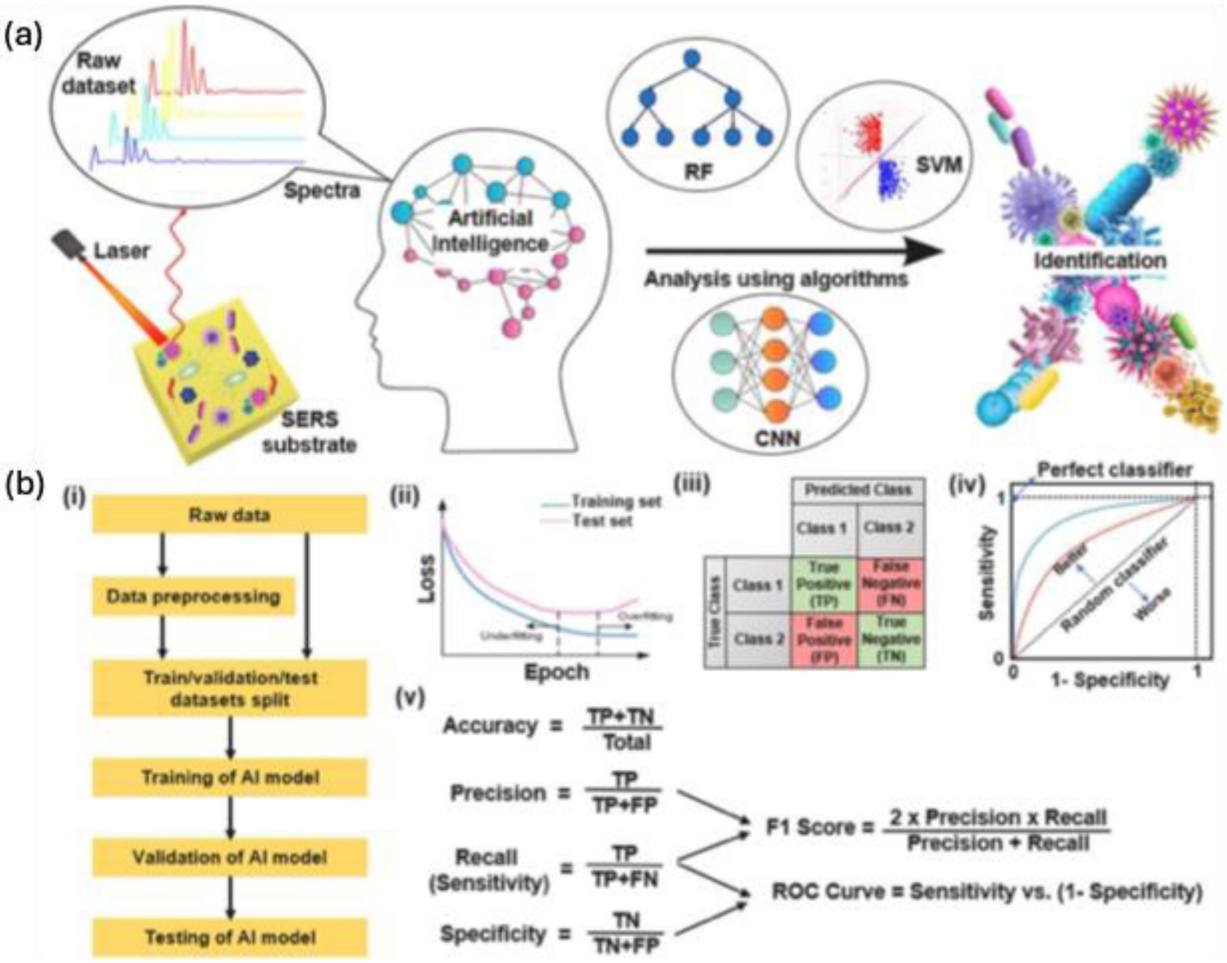
(a) Diagram illustrating the concept of utilizing AI algorithms for Raman spectroscopy analysis. (b) (i) Overview of the AI-driven data analysis workflow, (ii) loss curve observed during the model training phase, (iii) confusion matrix showing the comparison between actual and predicted outcomes, (iv) ROC curve indicating the balance between sensitivity and specificity, and (v) evaluation formulas for assessing the overall performance of the model**.** Reproduced with permission from 215,224. Copyright 2020, 2022, American Chemical Society.

AI-driven algorithms play a crucial role in SERS-based analysis, as illustrated in **Figure 26a** 215. Before applying these algorithms, spectral preprocessing steps such as noise reduction, baseline correction, and data normalization are essential to refine the raw dataset. Once preprocessed, AI models facilitate feature extraction to minimize overfitting in neural network training, ultimately aiding in the identification and differentiation of SERS spectra for unknown analytes 216,217. As depicted in **Figure 26b(i),** supervised learning models utilize labeled data to recognize patterns and correlations within the dataset 218,219. The dataset whether raw or preprocessed is typically divided into training, validation, and test sets. The training set is used to extract relevant features and optimize algorithm parameters, while the validation set assists in fine-tuning these parameters. The test set then evaluates the algorithm's overall performance. To maintain consistency, these subsets are generally drawn from a single comprehensive dataset to ensure they share a common distribution. The choice of a machine-learning model should align with the dataset's characteristics and the specific analytical objectives, whether for qualitative classification or quantitative measurement. If there is a significant drop in accuracy when evaluating the test set compared to the training set, it may indicate overfitting. During the training phase, the loss function values are visualized as a loss curve **(Figure 26b(ii)),** which helps assess training performance and detect overfitting or underfitting 192. Classification outcomes are represented using a confusion matrix as shown in **Figure 26b(iii**), from which the receiver operating characteristic (ROC) curve is derived to illustrate the trade-off between sensitivity and specificity **(Figure 26b(iv)**. Various evaluation metrics, including accuracy, precision, sensitivity, specificity, and the F1 score, are calculated using the confusion matrix. The F1 score, defined as the harmonic mean of precision and recall, ensures equal consideration of both metrics 220,221. As shown in **Figure 26b(v**), true positive, true negative, false positive, and false negative values are used to determine these performance indicators. The ROC curve provides an overall evaluation of model effectiveness by displaying the area under the curve (AUC). A higher AUC value, approaching 1, signifies better predictive accuracy, whereas an AUC of 0.5 suggests no meaningful prediction. Training models on extensive datasets enhances their ability to reliably detect the presence or absence of a specific analyte. The choice of an AI model depends on the characteristics of the dataset.

**Figure 27:**
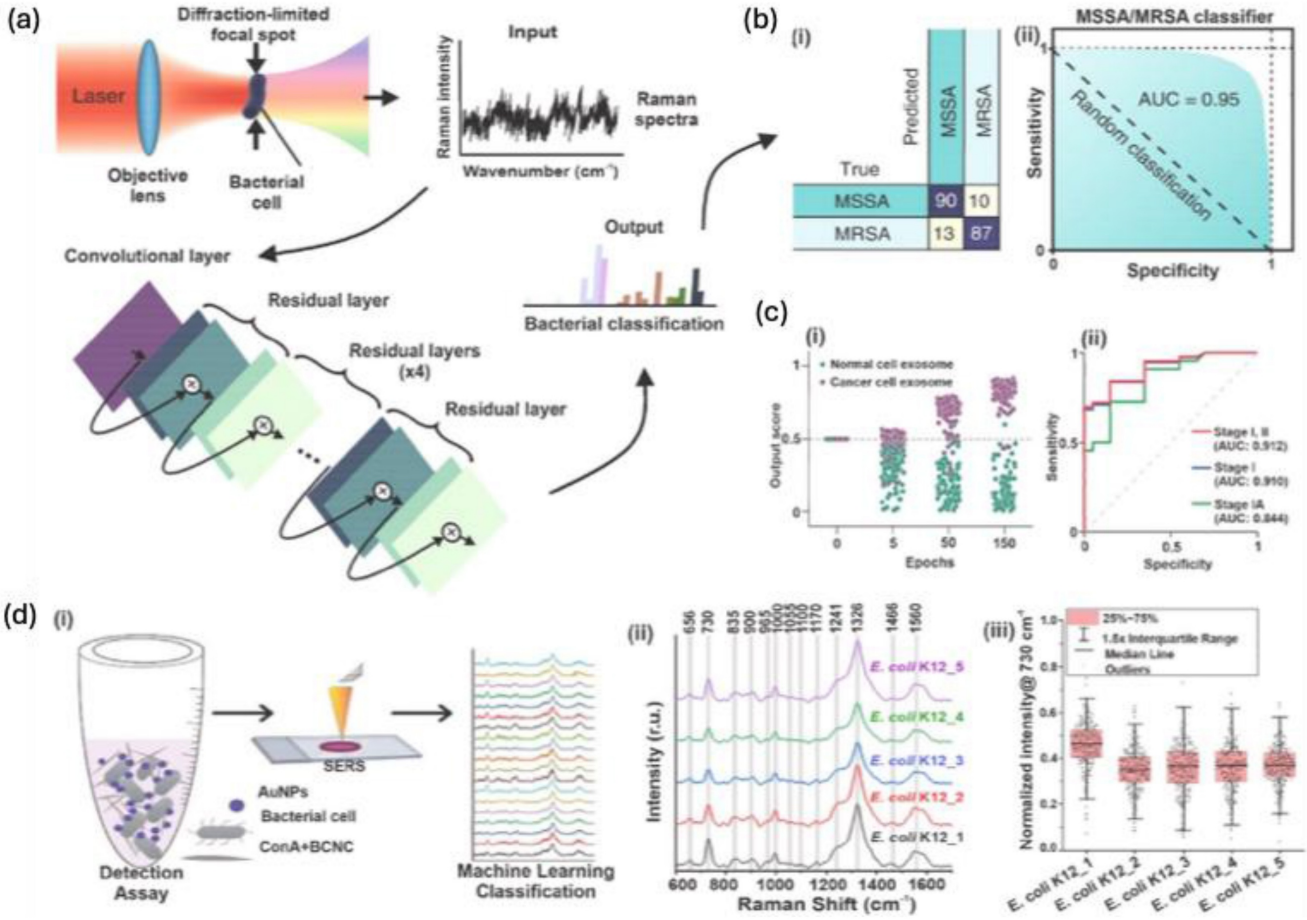
(a) Convolutional neural network (CNN) architecture for bacterial identification using Raman spectra from 30 bacterial species. (b) Binary classification of MRSA/MSSA: (i) Confusion matrix depicting classification performance and (ii) ROC curve illustrating sensitivity, specificity, and AUC value. (c) Deep learning-based exosome classification: (i) Data classification of normal vs. cancer cell-derived exosomes and (ii) ROC curves with AUC values corresponding to different cancer stages. (d) Surface-enhanced Raman spectroscopy (SERS) and machine learning analysis: (i) SERS data acquisition workflow, (ii) Representative SERS spectra from five replicates of E. coli K12, and (iii) Boxplot visualization of E. coli K12 spectral data distribution. Reproduced with permission from 225,227,233. Copyright 2019 Nature publishing group, 2020, 2022 American Chemical Society.

Deep learning, a subset of machine learning, involves training deep neural networks with multiple layers 222,223. These algorithms have enhanced SERS spectrum accuracy by automatically extracting features from raw data. In a typical convolutional neural network (CNN), there are three layers: 1) The convolutional layer, which applies preprocessing filters to signals, followed by a feature map; 2) The down-sampling (pooling) layer, which reduces the dimensions of the convolutional output to prevent overfitting; and3) The fully connected layer, which provides non-linear outputs using activation functions such as ReLU, GELU, or sigmoid. C.-S. Ho et al. used deep learning for bacterial identification based on SERS spectra, achieving 82.2% accuracy at the isolate level and 99.7% for treatment identification **(Figure 27a)** 225. They identified 30 bacterial pathogens using CNN with 25 convolutional layers, adapted from ResNet, which prevents the vanishing gradient problem through shortcut connections 226. The CNN model achieved 89% accuracy for MRSA vs. MSSA classification, with an AUC value of 0.95, indicating high sensitivity and specificity **(Figure 27b(i-ii)).** This approach has clinical potential. Shin et al. used ResNet for early lung cancer diagnosis with exosome-based SERS and deep learning, achieving high accuracy for classifying exosomes from normal and cancer cell lines, with an AUC value of 0.912 for stage I and II patients, and AUC values of 0.910 and 0.844 for stage I and IA patients, respectively **(Figure 27c(i-ii))** 227.

The attentional neural network (ANN) is a deep learning model that mimics biological neurons to process weighted inputs through nonlinear functions, generating meaningful outputs 192. ANN architectures, such as feedforward and recurrent networks, are widely used for tasks like classification, regression, and pattern recognition 228. In biomedical research, ANN has been applied to analyze metabolite gradients in cell lines using SERS, effectively distinguishing cancerous, healthy, and control cells 229. Guselnikova et al. leveraged ANN with SERS to detect UV-induced DNA damage, achieving over 98% accuracy 230. Similarly, Qin et al. developed an ANN model for identifying extracellular vesicles from pathogenic bacteria using label-free Raman spectroscopy, attaining accuracy rates exceeding 96% for bacterial classification and 93% for antibiotic resistance detection 231.

Support Vector Machine (SVM) is a binary classification model that undergoes multiple optimizations to establish an optimal hyperplane for spectral discrimination in high-dimensional spaces 192,232. It identifies supporting vectors data points near misclassified regions and iteratively adjusts their weights to maximize the separation margin between classes. For complex Raman spectral analysis, a kernel function is introduced to enhance spectral separation 233. Rahman et al. explored SVM's potential in bacteria isolation and label-free SERS detection using concanavalin A-modified bacterial cellulose nanocrystals combined with AuNPs **(Figure 27d(i))** 233. Their model achieved 87.7% accuracy in distinguishing 19 bacterial strains **(Figure 27d(ii))**. To assess spatial variability in E. coli K12, Raman spectra were averaged across five replicates, with a coefficient of variation of ~29.3%, indicating minimal variability **(Figure 27d(iii))**. Bakhtiaridoost et al. applied SVM to differentiate leukocytes from circulating tumor cells in breast cancer, using wavelet transform for noise reduction and feature extraction, achieving an accuracy of over 98.99% 234. Similarly, Sahin et al. developed Ag-CuxO nanostructures as antimicrobial, plasmonic surfaces to classify five bacterial species (E. coli, E. faecalis, S. aureus, B. subtilis, and S. mutans) using SVM, obtaining a 97% identification accuracy 235.

The studies showed that deep machine learning and AI techniques along with SERS can solve many of the complex issues existing in the bioanalytical field. Precise nanogap-engineered SERS platforms overcome many of the issues in the conventional Raman spectral analysis. However the complexity of many biological systems renders the SERS response of many systems, but AI technique can rectify these issues, and shortly, with the aid of new algorithm platforms we can fabricate many biosensors for biomedical applications and clinical trials.

## 8. Summary and Future Prospective

With the advancements in the fabrication of various nanostructures, researchers have found a gradual solution for the design and execution of SERS substrates for bio-sensing. Sophisticated methods and SERS materials can be economically produced by various techniques. The various top-down and bottom-up approaches enabled to fabrication of various SERS substrates which offer label-free or label-based detection of various bioanalytic and pathogens. Microfluidic techniques, paper-based assay, and small biochips embedded with SERS materials can be used for future clinical applications with high reproducibility. Due to the unique fingerprinting ability of the SERS technique, the detection methods can be applied *in vivo* as well. In one approach, Lv et al. showed that Au@Cu_2_-xS core-shell NPs can be used for cancer *in vivo* detection of cancer 236. The NP surface was modified with folic acid for the selective target detection in the folate receptors in cancer cells and cresol violet acetate is used as a Raman reporting molecule on the surface of Au. The plasmonic enhancement due to the coupling of Au and Cu2-xS resonance peaks offers an enhanced SERS signal and allows imaging of cancer cells, distinguishing the non-cancerous cells due to the specific folate receptor binding of the NPs. These NPs further showed nondestructive photothermal therapy using an 808 nm laser. Thus, SERS techniques can be conjugated with other methods for accurate and reproducible analysis.

Even though this is the scenario, selectivity is still a big issue, and several groups have tried to demonstrate different materials to avoid this issue. Concerning the success and results, SERS-based bioanalysis and trace detection strategies can bring up interesting new results soon. This review highlighted the importance of 3D-nano microstructure from Au or Ag as SERS substrates for bioanalysis. Although top-down and bottom-up approaches are used for 3D micro-nano structures, they possess certain limitations, and ongoing research can rectify these issues. Because top-down methods give structures with precise nanogaps, analytes that can enter the nanogap can be detected. More cost-effective and economical methods need to be developed so that soon SERS-based platforms can replace most of the analytical tools and methods for bioanalysis. Moreover, the recent AI algorithm and deep machine learning studies along with SERS platforms enabled the provision of valuable information from complex biological systems and multiple spectral data that can be easily resolved. The various algorithms like PCA, ANN, CNN, etc. can be used for other spectroscopic techniques along with Ra-man spectroscopy for more detailed clinical practices shortly.
